# Molecular Dynamics‐Guided Sterol Engineering of mRNA‐Lipid Nanoparticles Reprograms Biodistribution and Enhances Spleen‐Selective Immunity

**DOI:** 10.1002/advs.76671

**Published:** 2026-07-21

**Authors:** Sanghyuk Jeon, Seo‐Hyeon Bae, Jungyong Ji, Jisun Lee, Hosam Choi, Min‐Ho Kang, Sang‐In Park, Hyemin Kim, Nakyung Lee, Hajin Lee, Seonghoon Kim, Jungmin Kim, Subin Yoon, Seonghyun Lee, Seongje Cho, Dahyeon Ha, Ayoung Oh, Sohee Jo, Huijeong Choi, Yeeun Lee, Sowon Lee, Hyo‐Jung Park, Gitak Nam, Jisu Shin, Yujin Kang, Wonpil Im, Kiyoun Lee, Jae‐Hwan Nam

**Affiliations:** ^1^ Department of Medical and Biological Sciences The Catholic University of Korea Bucheon Republic of Korea; ^2^ BK Four Department of Biotechnology The Catholic University of Korea Bucheon Republic of Korea; ^3^ MolCube Inc. Seoul Republic of Korea; ^4^ Department of Chemistry The Catholic University of Korea Bucheon Republic of Korea; ^5^ Department of Biomedical‐Chemical Engineering The Catholic University of Korea Bucheon Republic of Korea; ^6^ Research Institute for Controlled Biomaterials of Regulated Cell Death The Catholic University of Korea Bucheon Republic of Korea; ^7^ Department of Biomedical Laboratory Science Daegu Haany University Gyeongsan Republic of Korea; ^8^ Department of Biological Sciences Lehigh University Bethlehem Pennsylvania USA; ^9^ National Institute of Health Korea Disease Control and Prevention Agency Cheongju Republic of Korea

**Keywords:** biodistribution, immune response, lipid nanoparticles, molecular dynamics, mRNA vaccines, sterol engineering

## Abstract

Multicomponent membrane organization of mRNA‐lipid nanoparticles (LNPs) critically determines their formulation behavior, organ selectivity, and immunological outcomes. However, compared to ionizable lipids, sterols remain a relatively underexplored design axis. This study discusses the engineering of a library of nine bile acid‐derived sterols with different hydroxylation patterns and alkyl tail lengths, and systematically maps how sterol structure governs formulation‐level properties and organ‐level expression profiles. After integrating physicochemical characterization with all‐atom molecular dynamics (MD) simulations, the experimentally observed formulation behaviors correlate with MD‐derived membrane structural descriptors. These descriptors provide a quantitative evaluation framework for prioritizing sterol chemotypes based on their predicted encapsulation performance and membrane organization, supporting the notion that sterol‐dependent membrane organization provides a structural basis for formulation properties, including mRNA encapsulation. Moreover, substituting cholesterol with bile acid‐derived sterols consistently attenuated hepatic expression and shifted organ‐level expression toward spleen‐dominant profiles, which is central to immune priming and adaptive immune activation. Among bile acid‐derived sterols, CA‐20 LNPs functionally enhance antigen‐specific humoral immunity and elicit antigen‐specific cellular immune responses, including improved memory‐associated immune features, while maintaining an acute safety profile. Collectively, these results establish sterol engineering as a powerful design strategy for modulating LNP formulation properties, in vivo fate, and immunological function.

## Graphical abstract

Sterol engineering enables precise control of mRNA lipid nanoparticles by tuning membrane organization. Replacing cholesterol with bile acid‐derived sterols redirects protein expression from the liver to the spleen. Notably, CA‐20 LNP enhances antigen‐specific immune responses while exhibiting a favorable safety profile, highlighting sterols as a powerful design axis for next‐generation mRNA vaccines.

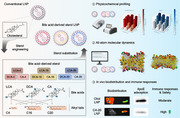

## 1 Introduction

mRNA is inherently unstable, and because of its polyanionic macromolecular nature, it cannot cross cellular membranes without delivery vehicles. Therefore, delivery systems that protect mRNA in vivo and enable efficient cellular entry with cytosolic access are key determinants of the clinical efficacy of mRNA therapeutics [1]. To meet these requirements, lipid nanoparticles (LNPs) have been established as a major delivery platform for nucleic acid therapeutics, with clinical success demonstrated by both mRNA vaccines and LNP‐based siRNA therapeutics [2, 3].

Clinically established mRNA‐LNP formulations comprise four components: an ionizable lipid, a helper phospholipid, a polyethylene glycol (PEG) lipid, and a structural sterol (most commonly cholesterol) [1, 4]. These components function individually and interact to collectively determine the LNP membrane organization, surface properties, stability, and in vivo behavior. Among them, ionizable lipids are regarded as key elements because they drive mRNA encapsulation (via electrostatic complexation) and mediate endosomal escape through protonation at the endosomal pH, leading to membrane destabilization [1, 5]. Accordingly, LNP design over the past several years has primarily focused on optimizing ionizable lipid structures, including headgroups, linkers, and hydrophobic tails [1, 6, 7]. Helper phospholipids contribute to membrane organization and mechanical stability, and a range of combinations and substitution strategies have been investigated to modulate membrane properties such as curvature and fusogenicity, influencing delivery efficiency and particle behavior [4, 8, 9]. PEG lipids are surface components that regulate colloidal stability in circulation and nonspecific interactions, and the effects of PEG lipid identity, length, molar ratio, and shedding behavior on in vivo performance have been extensively explored [4, 10].

Conventionally, cholesterol serves as the gold‐standard sterol component and is widely used as a structural scaffold to stabilize LNP structure, tune membrane fluidity/rigidity, and maintain particle integrity [4, 11]. However, sterols are less frequently considered primary optimization targets than ionizable lipids. Consequently, the structure‐function relationships connecting sterol molecular changes to membrane organization and in vivo behavior remain insufficiently systematized. Nevertheless, sterols are key formulation components that typically constitute the second‐largest fraction after ionizable lipids, suggesting that they represent an underutilized but potentially independent design variable capable of influencing LNP membrane organization and downstream in vivo behaviors. Component strategies have also been pursued, which involve adding extra components while maintaining the four core constituents. For example, selective organ targeting (SORT) approaches that modulate organ distribution (*e.g*., the liver, spleen, and lungs) by incorporating charged lipids/additives have been reported [12, 13], along with strategies that confer immunological advantages through cholesterol mannosylation [14], and designs that introduce functional ligands (such as peptides) onto PEG lipids to target specific tissues or organs, including tumors and the brain [15, 16].

Although each LNP component contributes differently to the formulation properties and biological interactions, the in vivo fate and organ selectivity after systemic administration remain difficult to predict and design. Most ionizable lipid‐based LNPs exhibit pronounced liver tropism following systemic dosing, which is a major limitation for applications requiring extrahepatic targeting [17]. This hepatic preference has often been attributed to apolipoprotein E (ApoE) adsorption on the LNP surface, highlighting how interactions between the LNP surface composition and circulating proteins can bias the organ‐level fate [18, 19]. In this context, accumulating reports have shown that even when the same ionizable lipid is used, organ‐level expression patterns can shift simply by altering other formulation components, strengthening the evidence that organ selectivity can be modulated through compositional control [9, 12]. Studies with varied sterol compositions and structures have reported shifts in expression efficiency and organ‐level distribution [20, 21, 22]. Despite these observations, most sterol modification studies have relied on partial substitution or empirical screening and have largely adopted a candidate‐by‐candidate, outcome‐focused evaluation paradigm rather than a hypothesis‐driven, structure‐guided sterol series design that enables trend‐level comparisons. Accordingly, the development of a generalizable and systematic structure‐function framework that links sterol structural changes via physicochemical properties and membrane organization to downstream outcomes, including expression efficiency, organ selectivity, and immunological readouts, has been limited. Consequently, it remains unclear whether the sterol structure can be used as a predictive model to rationally modulate LNP behavior across formulations.

In this study, rather than aiming to actively target a specific organ, we investigated whether sterol structure alone, when treated as an independent design variable, could reprogram LNP membrane organization and, consequently, in vivo expression patterns. To test this hypothesis, we introduced a structure‐guided strategy that replaced cholesterol with bile acid‐derived sterols. Bile acids share a steroidal backbone but allow diverse combinations of hydroxyl number/position and stereochemistry, providing a design advantage for tuning structural features such as particle stability, lipid packing, and membrane interactions at the molecular level. Moreover, rather than adding a fifth component, substituting cholesterol at an equivalent structural position preserves the clinically established LNP framework while enabling a more systematic validation of causal links among the structure, physicochemical properties, and in vivo behavior without an excessive increase in variables. By integrating experimental measurements with all‐atom molecular dynamics (MD) simulations, we aimed to establish structure‐derived descriptors that could inform sterol selection beyond empirical screenings. Accordingly, in this study, we redesigned bile acid scaffolds into fully LNP‐compatible structural sterols and sought to systematically connect and elucidate how the sterol chemical structure governs (i) LNP physicochemical properties (e.g., encapsulation efficiency, particle size, and surface charge), (ii) surface‐protein‐binding characteristics in circulation (particularly ApoE binding), and (iii) organ‐level expression and distribution patterns, along with immunogenicity and safety‐related readouts.

## 2 Results and Discussion

### 2.1 Construction of a Structurally Diverse Bile Acid‐Derived Sterol Library for LNP Formulation

**FIGURE 1:**
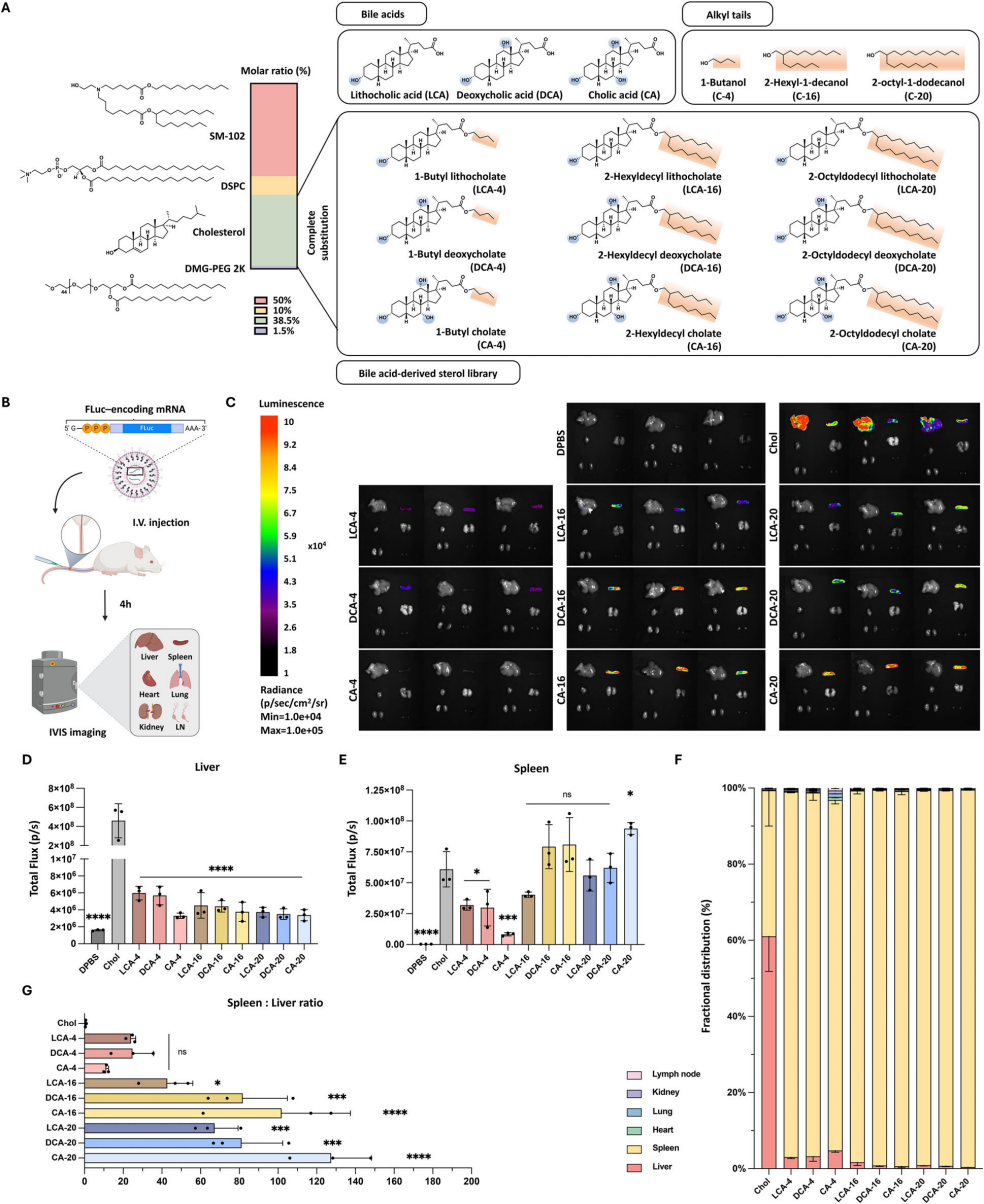
Design of bile acid‐derived sterol LNPs and reprogrammed organ‐level biodistribution. (A) Schematic illustration of LNP composition and the bile acid‐derived sterol library. (B) Experimental scheme for in vivo biodistribution analysis following intravenous administration of Firefly luciferase (FLuc)‐encoding mRNA‐LNPs (0.5 mg kg^−^^1^). Major organs (liver, spleen, heart, lung, kidney, and lymph nodes) were harvested 4 h after injection for IVIS imaging. (C) Representative IVIS images of excised major organs from female BALB/c mice (6–7 weeks old, *n* = 3 biologically independent animals) treated with DPBS, Chol LNPs, or bile acid‐derived sterol LNPs. (D,E) Quantification of total flux (photons/s) in the liver (D) and spleen (E) measured using IVIS imaging. (F) Fractional distribution of luciferase expression (%) across major organs, calculated using the average radiance (photons/s/cm^2^/sr). (G) Spleen‐to‐liver ratio of luciferase expression, representing relative splenic tropism, calculated using the average radiance (photons/s/cm^2^/sr). Data are presented as individual data points with the mean ± SD. Statistical analysis was performed using one‐way ANOVA with Dunnett's multiple comparisons test (^*^ *p <* 0.05, ^**^ *p <* 0.01, ^***^ *p <* 0.001, ^****^ *p <* 0.0001; ns, not significant), with all comparisons made relative to the Chol LNP group.

Based on the premise that the sterol chemical structure can influence the organ‐level expression patterns of mRNA‐LNPs, we constructed a structurally defined sterol library in which two key molecular parameters, the number of hydroxyl groups on the bile acid core and the length of the hydrophobic alkyl chain, were systematically varied, and organ‐level expression profiles were subsequently analyzed (Figure 1). This sterol library was designed to enable a controlled and systematic comparison of sterol‐dependent effects on LNP formulations by varying the sterol structure while maintaining all other formulation parameters constant.

As illustrated in Figure 1A, nine bile acid alkyl esters were synthesized using three bile acid scaffolds: lithocholic acid (LCA), deoxycholic acid (DCA), and cholic acid (CA). These bile acids were selected because they contain one, two, and three hydroxyl groups, respectively, thereby constituting a hydroxylation series that enables systematic evaluation of how sterol polarity and hydrophilicity influence LNP behavior. To further modulate sterol structure, the esterification of the C24 carboxyl group with one of three alcohols differing in chain length and branching (C4, C16, and C20) was performed. Specifically, each bile acid was esterified with 1‐butanol (C4; linear primary alcohol), 2‐hexyl‐1‐decanol (C16; monobranched 2‐alkyl‐1‐alkanol bearing a C6 side chain at the C2 position), or 2‐octyl‐1‐dodecanol (C20; monobranched 2‐alkyl‐1‐alkanol bearing a C8 side chain at the C2 position). Branching at the C2 position generates a Y‐shaped hydrophobic tail with increased steric volume proximal to the ester linkage, whereas increasing alkyl‐chain length progressively expands the hydrophobic volume. The selected alkyl tails were intended to represent distinct degrees of hydrophobic expansion within the bile acid scaffold. Specifically, C4 was chosen as a short alkyl chain providing minimal hydrophobic extension, whereas C16 and C20 were selected to represent intermediate and greater increases in hydrophobic volume, respectively. This strategy enabled the construction of a structurally defined hydrophobic volume series for systematically evaluating the effects of hydrophobic and steric volume on LNP behavior while maintaining a chemically manageable library size. All compounds were synthesized as described in Supplementary Methods. In this study, these bile acid alkyl esters are collectively referred to as bile acid‐derived sterols. Together, these design variables defined a cholesterol‐centered sterol chemical space that enabled systematic evaluation of how structural variations in sterols arising from differences in hydroxylation pattern and hydrophobic volume influence LNP behavior.

Bile acid‐derived sterols were incorporated into a clinically relevant SM‐102‐based LNP formulation (SM‐102:DSPC:sterol:DMG‐PEG2000 = 50:10:38.5:1.5, mol %) to replace cholesterol, which served as a reference formulation. This design enabled the selective modulation of sterol polarity and hydrophobic volume while preserving the overall lipid composition. The detailed chemical structures and formulation compositions are summarized in Tables S1 and S2. All LNP formulations were prepared under identical microfluidic mixing conditions, providing an experimental framework that allows for the direct comparison of physicochemical and biological properties attributable specifically to the sterol chemical structure rather than formulation‐related variability. This structurally defined sterol library provided a basis for the subsequent evaluation of sterol‐dependent differences in LNP physicochemical properties, membrane organization, and in vivo fate.

### 2.2 Bile Acid‐Derived Sterol Substitution Induces a Marked Shift in Biodistribution With Reduced Hepatic Expression

First, we examined whether substituting cholesterol with bile acid‐derived sterols alters the organ‐level distribution of mRNA expression following systemic administration. BALB/c mice were intravenously administered LNPs encapsulating Firefly luciferase (FLuc) mRNA at a dose of 0.5 mg kg^−^^1^ (Figure 1B). Major organs, including the liver, spleen, heart, lungs, kidneys, and lymph nodes, were harvested 4 h post‐injection, a time point commonly used to capture organ‐level expression patterns in mRNA‐LNP biodistribution studies [23]. Hereafter, organ‐level expression was quantified by in vivo imaging system (IVIS) luminescence and used as a functional readout. Representative IVIS imaging (Figure 1C) showed that cholesterol‐containing LNPs (Chol LNPs) produced strong luciferase expression, predominantly in the liver, recapitulating the well‐established liver‐centered expression profile of conventional LNPs [24, 25]. In contrast, LNPs formulated with bile acid‐derived sterols markedly reduced hepatic expression. This distribution pattern was consistent across all the bile acid and alkyl chain variants. Collectively, these results indicate that bile acid‐derived sterol substitution broadly attenuates liver‐dominant gene expression following intravenous administration.

The quantitative analysis of the total photon flux further supports these observations. As shown in Figure 1D, the hepatic expression levels of bile acid‐derived sterol LNPs were reduced by approximately 77–139‐fold relative to those of Chol LNPs. In contrast, splenic expression varied depending on the sterol structure, ranging from lower to comparable or higher levels relative to Chol LNPs (Figure 1E). CA‐20 LNPs exhibited approximately a 1.6‐fold increase in splenic expression compared to Chol LNPs. Analysis of fractional organ distribution revealed a pronounced redistribution of overall expression (Figure 1F). Whereas Chol LNPs allocated ∼61% of the total expression to the liver and 38% to the spleen, bile acid‐derived sterol LNPs displayed a dramatic reduction in hepatic contribution (0%–5%), accompanied by a dominant splenic fraction (92%–99%). Among these formulations, CA‐20 LNPs showed the most extreme redistribution, with approximately 0.5% of the total expression in the liver and approximately 99.1% localized to the spleen. The spleen‐to‐liver expression ratio provides a metric for evaluating relative organ preference, independent of absolute expression levels [22]. Accordingly, spleen‐to‐liver expression ratios were calculated (Figure 1G). All bile acid‐derived sterol LNPs exhibited significantly elevated spleen‐to‐liver ratios compared to Chol LNPs, with CA‐20 displaying the highest ratio. This metric indicates a structural shift in relative organ tropism that is independent of absolute expression levels.

To further validate the spleen‐associated expression pattern observed by IVIS imaging, we performed an independent flow cytometric analysis using eGFP‐encoding mRNA‐LNPs following intravenous administration (Figure S1A). Consistent with the IVIS results, the relative differences in expression levels observed among the bile acid‐derived sterol LNPs were similarly reproduced in eGFP expression across total splenocytes. In particular, DCA‐16 LNP, CA‐16 LNP, and CA‐20 LNP, which exhibited FLuc expression levels in the spleen that were comparable to or higher than those of Chol LNP, also showed elevated eGFP expression by flow cytometry. Among the tested formulations, CA‐20 LNP again exhibited the highest level of expression, consistent with the IVIS findings (Figure S1B). Furthermore, splenic FLuc expression measured by IVIS showed a relatively strong correlation with eGFP expression measured by flow cytometry (R^2^ = 0.69, Figure S1C), supporting the overall agreement between organ‐level expression assessed by IVIS and cellular‐level expression assessed by flow cytometry. The eGFP expression trend observed in total splenocytes was similarly reflected across major splenic immune‐cell populations, including macrophages, dendritic cells, B cells, and T cells (Figure S1D–G). Notably, relatively high levels of eGFP expression were observed in professional antigen‐presenting cells (APCs), including macrophages, dendritic cells, and B cells. Collectively, these results provide independent cellular‐level validation of the spleen‐associated expression pattern observed by IVIS imaging and suggest that bile acid‐derived sterol LNPs facilitate mRNA delivery to diverse immune‐cell populations within the spleen.

To assess whether this redistribution reflected an intrinsic consequence of sterol substitution rather than a phenomenon specific to intravenous exposure, BALB/c mice received the same SM‐102‐based LNP formulations via intramuscular (IM) injection (Figure S2). Under IM conditions, bile acid‐derived sterol LNPs showed substantially reduced hepatic expression relative to cholesterol LNPs, with preferential expression in the spleen and muscles. Fractional distribution analysis revealed that cholesterol LNPs were distributed with approximately 21% of the total expression in the liver, 69% in the spleen, and 9% in the muscle, whereas bile acid‐derived sterol LNPs reduced the liver contribution to approximately 2%–8% and redistributed 75%–95% of the total expression in the spleen and muscle. CA‐20 exhibited the most pronounced extrahepatic redistribution (liver, 2%; spleen, 67%; muscle, 29%), and all bile acid‐derived sterol LNPs showed increased spleen‐to‐liver ratios.

To determine whether the observed biodistribution patterns were specific to the SM‐102‐based LNPs, the same bile acid‐derived sterol library was applied to the DLin‐MC3‐DMA (MC3)‐based LNPs and evaluated (Figure S3). Cholesterol‐containing MC3 LNPs exhibited a strong liver‐dominant distribution, with approximately 86% of the total expression localized in the liver and approximately 12% in the spleen. In contrast, substitution with bile acid‐derived sterols markedly reduced the hepatic fraction to 25%–58% and increased the splenic distribution to 41%–74%, recapitulating the redistribution trend observed in SM‐102‐based LNPs. Within the MC3 platform, certain sterols, such as DCA‐4 and CA‐16, produced relatively higher splenic expression, suggesting that the optimal bile acid‐derived sterol structure may depend on the identity of the ionizable lipid and the overall LNP composition. Nevertheless, an overarching trend of attenuated hepatic expression accompanied by enhanced spleen‐centered distribution upon cholesterol replacement was consistently observed across both ionizable lipid platforms. These results suggest that the modulation of biodistribution via bile acid‐derived sterol substitution is not restricted to specific ionizable lipids.

Further, we showed that complete replacement of cholesterol with bile acid‐derived sterols was sufficient to induce considerable alterations in the systemic distribution profile of mRNA‐LNPs. This alteration is characterized by a marked reduction in hepatic expression, accompanied by a redistribution of expression toward extrahepatic organs, in contrast to the canonical liver‐centered distribution pattern of conventional LNPs. However, the present results alone are insufficient to elucidate the underlying principles and mechanisms by which these changes in distribution are mediated. Previous studies have consistently reported that the intrinsic chemical properties of the constituent lipids, the resulting physicochemical characteristics of LNPs, and the nature of the protein corona formed under physiological conditions collectively play critical roles in governing the in vivo behavior and organ selectivity of LNPs [13, 26]. The chemical features of bile acid‐derived sterols, which are structurally distinct from cholesterol, may influence key physicochemical parameters such as mRNA encapsulation efficiency, particle size, surface charge, and particle homogeneity. These parameters are likely closely associated with the biological fate of LNPs in vivo. In the following section, we systematically analyze the physicochemical properties of bile acid‐derived sterol LNPs and interpret the observed distribution changes from a structure‐property perspective.

### 2.3 Structural and Physicochemical Comparison Between Cholesterol and Bile Acid LNPs

As an initial step toward defining the structural requirements for sterol function in mRNA‐LNPs, we evaluated whether unmodified bile acids could be directly incorporated into the canonical LNP formulations. Before analyzing the physicochemical properties of bile acid‐derived sterol LNPs, we formulated their precursors, unmodified bile acids, under identical conditions and characterized their physicochemical profiles. These results motivated us to synthesize bile acid‐derived sterols (Figure S4). Among the evaluated parameters, the mRNA encapsulation efficiency (EE) most clearly highlighted the structural and physicochemical differences between cholesterol and bile acids. Chol LNPs exhibited a high EE of 94.2%, whereas LNPs formulated with unmodified bile acids showed a markedly reduced EE, with values of 2.5% for LCA, 22.0% for DCA, and 28.8% for CA. In addition, bile acid LNPs exhibited slightly smaller particle sizes, reduced assembly uniformity, and a tendency toward a more negative surface charge. Taken together, these results indicate that unmodified bile acids do not meet the physicochemical requirements of canonical LNP formulations for stable mRNA encapsulation and are therefore unsuitable as structural sterols for mRNA‐LNPs.

To further interpret these limitations, we analyzed the structural features of cholesterol and bile acids. As summarized in Figure S5, bile acids differ from cholesterol in several key structural aspects that are likely to influence the lipid association patterns and hydrophobic balance within LNPs. Cholesterol contains a *trans*‐fused A/B ring junction, a single C3‐β hydroxyl group, and a long hydrophobic alkyl tail at C17, which together form a planar, compact, and rigid sterol scaffold [27, 28]. In contrast, bile acids possess *cis*‐fused A/B rings that bend the molecular plane by nearly 90° [29], thereby reducing the planarity of the steroid framework [30, 31]. Moreover, lithocholic acid contains one hydroxyl group at C3‐α, deoxycholic acid contains two (C3‐α and C12‐α), and CA contains three (C3‐α, C7‐α, and C12‐α), representing a progressive increase in core polarity. Unlike cholesterol, which bears a hydrophobic alkyl tail at C17, bile acids terminate in a carboxyl group at C24, providing an additional hydrogen‐bonding site and substantially altering amphiphilicity [32]. Although the precise quantitative contributions of individual structural features such as polarity, hydroxylation patterns, and A/B ring geometry remain to be fully elucidated, these molecular distinctions provide a structural basis for interpreting the limitations observed when bile acids are directly incorporated into LNPs as sterol components.

### 2.4 Comparative Physicochemical Analysis of Cholesterol and Bile Acid‐Derived Sterol LNPs

These intrinsic limitations of unmodified bile acids motivated the design of bile acid‐derived sterols to restore the hydrophobic‐hydrophilic balance and recover the cholesterol‐like packing behavior. Accordingly, we systematically analyzed the structure‐property relationships across sterol cores and tail architectures to elucidate how alkyl‐tail conjugation modulates the physicochemical behavior of bile acid‐derived sterols in LNPs. We evaluated the resulting structure‐function relationships by comparing the physicochemical properties of the nine bile acid‐derived sterols with those of their corresponding unmodified bile acids. EE, Z‐average, Zeta potential, and polydispersity index (PDI) were evaluated (Table 1).

**TABLE 1:** Physicochemical characterization of cholesterol, bile acid, and bile acid‐derived sterol LNPs.

LNP	EE (%)	Z‐average (nm)	PDI	Zeta potential (mV)
Chol	94.2 ± 1.2	95.8 ± 1.7	0.05 ± 0.02	16.6 ± 0.3
LCA	2.5 ± 1.1	81.0 ± 0.9	0.12 ± 0.01	−24.0 ± 1.5
DCA	22.0 ± 3.3	64.6 ± 1.8	0.17 ± 0.01	−22.6 ± 2.7
CA	28.8 ± 11.1	77.0 ± 1.4	0.11 ± 0.01	−14.3 ± 0.9
LCA‐4	70.7 ± 0.3	92.2 ± 0.6	0.08 ± 0.01	−5.5 ± 0.4
DCA‐4	43.0 ± 8.2	83.6 ± 0.7	0.05 ± 0.02	11.0 ± 0.6
CA‐4	36.4 ± 3.9	80.8 ± 0.5	0.10 ± 0.01	9.4 ± 1.8
LCA‐16	76.8 ± 2.0	120.5 ± 1.0	0.05 ± 0.01	1.9 ± 0.2
DCA‐16	87.4 ±0.7	118.0 ± 0.3	0.02 ± 0.01	7.8 ± 0.2
CA‐16	72.9 ± 0.1	102.4 ± 0.6	0.05 ± 0.01	15.0 ± 0.6
LCA‐20	78.4 ± 2.0	135.2 ± 1.0	0.03 ± 0.01	−1.0 ± 0.6
DCA‐20	90.8 ± 0.8	128.7 ± 0.6	0.06 ± 0.01	12.6 ± 0.6
CA‐20	87.4 ± 0.6	118.3 ± 0.4	0.04 ± 0.02	7.1 ± 2.2

*EE (*n* = 3) and Z‐average, PDI, Zeta potential (*n* = 4) are shown as mean ± standard deviation.

**FIGURE 2:**
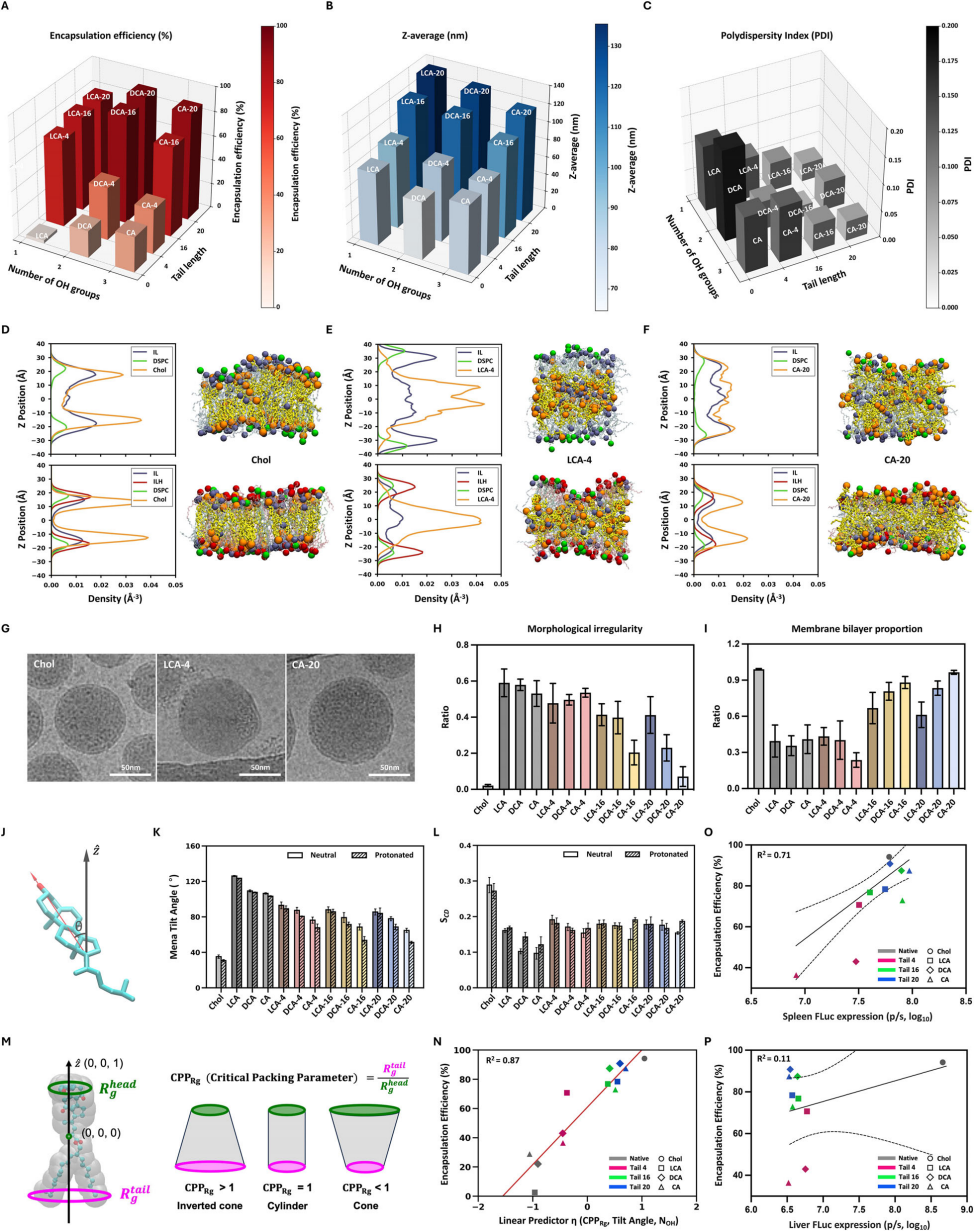
Physicochemical properties, cryo‐TEM characterization, and all‐atom molecular dynamics analysis of bile acid‐derived sterol LNPs. A total of 13 LNP formulations were analyzed, including Chol LNP, three unmodified bile acid‐containing LNPs, and nine bile acid‐derived sterol LNPs, focusing on variations arising from the number of hydroxyl groups in the bile acid core and the alkyl chain length. (A) Encapsulation efficiency (EE) of mRNA‐LNPs, (B) Z‐average hydrodynamic diameter measured using dynamic light scattering, and (C) polydispersity index (PDI) of unmodified bile acid and bile acid‐derived sterol LNPs. (D–F) All‐atom molecular dynamics snapshots (side view) and Z‐density profiles of ionizable lipids (IL, ILH for protonated IL), DSPC, and sterol headgroups in LNP bilayer systems containing (D) Chol, (E) LCA‐4, and (F) CA‐20. The bilayer center is located at Z = 0. (G) Representative high‐magnification cryo‐TEM images of LNPs containing Chol, LCA‐4, and CA‐20. Scale bar = 50 nm. (H, I) Quantitative analysis of morphological irregularity (surface protrusion, non‐spherical, emulsion‐like structure, and bleb‐like structure) and membrane bilayer proportion across the 13 LNP formulations. (H) Ratio of LNPs exhibiting morphological irregularity. (I) Ratio of LNPs exhibiting a discernible membrane bilayer structure. (J) Schematic representation defining the sterol tilt angle (θ) as the angle between the sterol ring's principal axis and the Z‐axis. (K) Mean tilt angles of bile acid‐derived sterols under neutral and protonated conditions. (L) Ensemble‐averaged lipid acyl‐chain order parameters, S_CD_. (M) Illustration of the critical packing parameter calculated from the radii of gyration; CPP_Rg_ with CA‐20 is used as an example. (N) Scatter plot of encapsulation efficiency vs. the expanded linear predictor η derived from CPP_Rg_, tilt angle, and the number of hydroxyl groups on the sterol core (N_OH_), with a linear fit (R^2^ = 0.87). (O) Scatter plot of encapsulation efficiency vs. splenic FLuc expression (p/s, log_10_) with a simple linear regression fit (solid line) and 95% confidence bands (dashed line), yielding an R^2^ value of 0.71. (P) Scatter plot of encapsulation efficiency vs. hepatic FLuc expression (p/s, log_10_) with a simple linear regression fit (solid line) and 95% confidence bands (dashed line), with R^2^ = 0.11. (N–P) Each point represents an LNP formulation. Point colors denote tail length series (Native (unmodified), C4, C16, and C20), and point shapes denote sterol scaffolds (Chol, LCA, DCA, and CA), as indicated in the legend.

Hydrophobic‐tail conjugation led to an overall improvement in EE across all bile acid classes (Figure 2A). However, both the magnitude and pattern of improvement strongly depended on the alkyl‐tail structure and hydroxyl‐group count of the bile acid core. Two tail‐structure effects were apparent: (i) the effect of short linear C4 tails and (ii) the effect of extended branched C16/C20 tails. For LCA, which contains a single hydroxyl group, conjugation with a short linear C4 alkyl tail (LCA‐4) resulted in a pronounced increase in EE relative to that of unmodified LCA. This observation suggests that even minimal hydrophobic tail addition can improve EE of LCA, potentially reflecting structural differences from cholesterol, such as distinct sterol‐ring geometry and the presence of a terminal carboxyl group. In contrast, for highly hydroxylated bile acids (DCA and CA), the same C4 tail conjugation (DCA‐4 and CA‐4) led to only modest improvements in EE. As the alkyl tails became longer and more branched (C16 and C20), divergent trends emerged across the bile acid classes. For the DCA and CA derivatives, extended and branched hydrophobic tails produced dramatic and monotonic increases in EE, with DCA‐16 (87.4%), CA‐20 (87.4%), and DCA‐20 (90.8%) all exceeding 80%. This substantial recovery of EE suggests that the synergistic restoration of hydrophobic volume and sterol anchoring effectively counterbalances the high polarity of multi‐hydroxylated steroid cores. In contrast, LCA derivatives showed only limited additional gains with further tail elongation (LCA‐4, 70.0%; LCA‐16, 76.8%; LCA‐20, 78.4%), indicating a weaker dependence on tail length than that observed for DCA and CA derivatives. These trends indicate that the recovery of EE requires the coordinated tuning of both the sterol core polarity and alkyl‐tail structure.

Z‐average analysis further showed that alkyl‐tail conjugation induced structure‐dependent changes in the particle size distribution (Figure 2B). All bile acid‐derived sterol LNPs exhibited hydrodynamic diameters between 80 and 135 nm, which are suitable for in vivo delivery [33]. Two structural determinants governed these particle size trends: (i) bile acid core hydroxylation and (ii) hydrophobic tail length and branching. The particle size decreased with an increase in the number of hydroxyl groups (LCA > DCA > CA). This trend may arise because the additional hydroxyl groups promote a tighter interfacial organization through enhanced interfacial interactions, contributing to the formation of smaller nanostructures. Within each bile acid class, the particle size increased with the tail length (C4 < C16 < C20). Similar observations have been reported for lipid assemblies, where the extension of hydrophobic tails increases the particle size [34].

Zeta potential analysis provided additional evidence that tail conjugation restored the cholesterol‐like physicochemical characteristics (Figure S6). The unmodified bile acid LNPs exhibited negative surface charges (−24.0 mV for LCA, −22.6 mV for DCA, and −14.3 mV for CA). In contrast, all bile acid‐derived sterol LNPs shifted toward neutral or mildly positive values (−1 to +15 mV), approaching the profile of cholesterol LNPs (+16.6 mV). PDI provided complementary insights into the packing uniformity (Figure 2C). Unmodified bile acid LNPs displayed broader size distributions (0.11–0.17), reflecting relatively non‐uniform and unstable self‐assembly driven by excessive polarities. In contrast, all bile acid‐derived sterols exhibited PDI < 0.10, forming monodisperse and homogeneous populations comparable to or, sometimes, exceeding the uniformity of Chol LNPs (0.05 ± 0.02). These observations suggest that the addition of a hydrophobic tail stabilizes the self‐assembly process and promotes a more coherent sterol organization within the nanoparticle cores.

Alkyl‐tail conjugation restored multiple key physicochemical properties of bile acid‐derived sterol LNPs, including enhanced EE, particle size normalization through the interplay between sterol polarity and tail hydrophobicity, markedly improved homogeneity reflected by reduced PDI, and a shift in Zeta potential toward a cholesterol‐like profile. These structure‐property relationships established sterol polarity (hydroxyl content) and tail hydrophobicity (length and branching) as synergistic determinants of the physicochemical properties and overall stability of LNPs.

### 2.5 All‐Atom MD Simulations Link Sterol Structure to Physicochemical Properties of Bile Acid‐Derived Sterol LNPs

To obtain atomistic insights into the structure‐property relationships, we performed all‐atom MD simulations to investigate how the sterol structure modulates the membrane organization and physicochemical properties of ionizable lipid bilayers. Our current simulation systems do not cover the entire LNP, which is beyond the size limit of all‐atom MD. However, we aimed to explore potential descriptors that could be correlated with experimental observations, such as EE, by simulating symmetric bilayer systems of a small patch of the outer leaflet of the LNP facing the outer leaflet of the plasma membrane or the inner leaflet of the endosomal membrane (Figure S7). Two protonation conditions were modeled: a neutral condition system (hereafter referred to as the neutral system), where all ionizable lipids were neutral, and a protonated condition system (hereafter referred to as the protonated system), where 50% of the ionizable lipids were protonated; the former corresponded to the extracellular environment, and the latter mimicked the endosomal low pH environment. Notably, we did not include PEG‐lipids to reduce the system size, as our previous simulation study has demonstrated that the membrane properties calculated from MD simulations were almost identical with and without PEG‐lipids [35]. Figure 2D–F show representative simulation snapshots (bilayer side view) of LNP bilayer systems containing cholesterol, LCA‐4, and CA‐20, whereas Figures S8–S11 show the complete results for all bile acid and bile acid‐derived sterol LNP bilayer systems, and the reference Chol LNP bilayer systems are shown in Figure S12.

Figure 2D–F and Figures S10 and S11 show the Z‐density distribution of the ionizable lipid (SM‐102), helper lipid (DSPC), and sterol headgroups, averaged over the simulation time; the membrane normal is along the Z‐axis, and its center is at Z = 0. In all neutral and protonated systems, except for the protonated CA‐16 and CA‐20 LNP bilayer systems, neutral ionizable lipids exhibited variable behaviors, with some remaining at the membrane‐water interface and others buried within the hydrophobic core, whereas all protonated ionizable lipid headgroups were well positioned at the interface. Note that such a neutral ionizable lipid distribution is dynamic rather than static. In the protonated Chol LNP bilayer systems, neutral ionizable lipids were much less populated at the bilayer center, whereas some portion was found in the neutral systems (Figure S12), which is like those in the CA‐16 and CA‐20 LNP bilayer systems. Regarding bile acid‐derived sterol distributions along the membrane normal, except for the protonated CA‐16 and CA‐20 systems, all neutral and protonated bilayer systems exhibited pronounced sterol clustering near the bilayer midplane, forming sterol core‐localized aggregates reminiscent of previously reported sterol‐sterol associations and central aggregation behavior in related systems [36]. In contrast, the CA‐16 and CA‐20 LNP bilayer systems, which possess multiple hydroxyl groups and the longest hydrophobic tail among the tested bile acid‐derived sterols, maintained Z‐density distributions closely resembling those in the Chol LNP bilayer systems under both neutral and protonated conditions (Figures S10–S12). The resemblance became even more evident upon protonation, suggesting enhanced interfacial stabilization with protonated ionizable lipids because of polar interactions between protonated ionizable lipids and the multiple hydroxyl groups in CA‐16 and CA‐20.

To determine whether the membrane organizational differences predicted by the MD simulations were reflected in the experimentally observed LNP structures, cryo‐transmission electron microscopy (cryo‐TEM), a powerful technique for directly visualizing the nanostructural features of LNPs, was performed [37]. The structural morphologies of LNP formulations prepared with Chol, three unmodified bile acids, and nine bile acid‐derived sterols were evaluated by cryo‐TEM, and the results are presented in Figures S13 and S14. Overall, the cryo‐TEM observations supported the trends observed in the physicochemical characterization and revealed distinct differences in particle morphology and size distribution depending on sterol structure. Chol LNPs exhibited relatively uniform particle sizes and spherical morphologies, whereas LNPs containing unmodified bile acids and short‐tail sterols generally showed smaller particle sizes with broader size distributions. In addition, increasing alkyl tail length was associated with more homogeneous particle populations and reduced size heterogeneity, a trend that was particularly evident in the DCA‐16, CA‐16, DCA‐20, and CA‐20 formulations. Collectively, these cryo‐TEM observations were consistent with the overall trends observed in the Z‐average and polydispersity index (PDI) measurements obtained by dynamic light scattering (DLS) (Figure 2B,C).

To examine whether the membrane structural features predicted by the MD simulations were reflected in the experimentally observed LNP morphologies, high‐magnification cryo‐TEM images of representative formulations (Chol, LCA‐4, and CA‐20) were compared with their corresponding MD simulation results (Figure 2D–G). Chol LNPs exhibited highly spherical morphologies with clearly discernible membrane bilayer structures across the particle surface [38], and CA‐20 likewise displayed relatively regular spherical morphologies and well‐defined membrane bilayers. These observations were generally consistent with the more organized membrane arrangements observed in the MD simulations. In contrast, LCA‐4 frequently exhibited irregular membrane contours and less distinct bilayer structures, resembling the relatively less organized membrane organization predicted by MD simulations. Notably, LCA‐4 showed a more heterogeneous distribution of ionizable lipids and sterols compared with Chol and CA‐20, together with a tendency for sterol headgroups to cluster near the bilayer center. These molecular‐level differences may be reflected in the increased membrane irregularity and reduced bilayer visibility observed in the cryo‐TEM images.

To systematically evaluate these observations across all formulations, morphological irregularity and membrane bilayer proportion were quantified (Figure 2H,I). Morphological irregularity was defined by the presence of surface protrusions [39], non‐spherical morphologies [40], emulsion‐like structures [41], and bleb‐like structures [42]. Chol LNPs exhibited the lowest level of morphological irregularity and the highest membrane bilayer proportion. In contrast, formulations containing unmodified bile acids and short‐tail sterols generally showed increased morphological irregularity and reduced membrane bilayer visibility. In many of these formulations, the membrane bilayer was only weakly visible or rarely observed on the particle surface, suggesting pronounced bilayer thinning and/or membrane structural instability arising from poor lipid organization [37]. By comparison, long‐tail sterol formulations, particularly CA‐16 and CA‐20, exhibited lower morphological irregularity and higher membrane bilayer proportions. These cryo‐TEM‐based analyses were also broadly consistent with the trends observed for Z‐average and PDI, suggesting that differences in membrane organization predicted by MD simulations are reflected in the experimentally observed LNP structures. Collectively, the physicochemical characterization, cryo‐TEM observations, and MD simulation results support the notion that sterol‐dependent membrane organization is closely associated with both the morphology and physicochemical properties of LNPs.

To gain further mechanistic insight into the membrane organizational differences among formulations, we analyzed the sterol tilt angle and acyl‐chain order parameter derived from the MD simulations. Tilt‐angle analysis (Figure 2J,K and Figure S15), which measures the angle between the principal axis of the sterol ring and the Z‐axis, revealed systematic structure‐orientation relationships. The tilt angle of cholesterol has a narrow distribution centered around 20° and shows a minor peak at approximately 160° because of its flip‐flop in a typical bilayer, such as the protonated Chol LNP bilayer system. For bile acid‐derived sterols, at a fixed tail length, the mean tilt angle (θ) decreased with increasing ring hydroxylation from LCA to DCA to CA, reflecting stronger interfacial anchoring. At fixed hydroxylation, the mean tilt angle decreased progressively with tail elongation from C4 to C16 to C20, consistent with the improved hydrophobic alignment and reduced interfacial disorder, as is evident from the representative snapshots and Z‐density profiles (Figures S8–S11). Consequently, θ in the CA‐16 and CA‐20 LNP bilayer systems best approached those of the Chol LNP bilayers in terms of both the mean and distribution, whereas other bile acid‐derived sterols exhibited large tilt angles (Table S3 and Figure S15). Furthermore, the structural significance of the sterol tilt angle identified by MD simulations was supported by cryo‐TEM analysis (Figure S16). Morphological irregularity and membrane bilayer proportion quantified from cryo‐TEM images showed positive (R^2^ = 0.77, Figure S16A) and negative (R^2^ = 0.56, Figure S16B) correlations with the sterol tilt angle, respectively. These results indicate that sterol orientation within the membrane is closely linked to LNP membrane architecture and morphology, providing experimental support that the molecular‐level membrane organization predicted by LNP bilayer MD simulations can be translated into observable structural features of LNPs. Consistent with the sterol tilt‐angle distributions and acyl‐chain order parameter (see Experimental Section for its calculation) of the bile acid‐derived sterol LNP bilayer systems were lower and thus more fluid than those of the Chol systems under both conditions, but higher and thus more ordered than those of the bile acid LNP bilayer systems (Figure 2L).

To quantitatively link the simulation and experiment, we adopted the classical critical packing parameter (CPP) [43], a dimensionless descriptor that relates a lipid shape to preferred lipid phases, and applied this framework to sterols using the radii of the gyration‐based metric CPP_Rg_ (Figure 2M; see Experimental Section for its calculation). According to this definition, conical lipids with a large head group and a small tail group have CPP_Rg_ < 1 and form a bilayer with a positive curvature similar to that of a micelle, whereas inverted conical lipids with a small polar head group and a large hydrophobic‐tail group have CPP_Rg_ > 1 and form a bilayer with a negative curvature. CPP_Rg_ ≈ 1 indicates cylindrical lipids that form planar bilayers. The CPP_Rg_ increased with the tail length of the bile acid‐derived sterols and differed only marginally between the neutral and protonated conditions (Figure S17). In our study, CPP_Rg_ was positively correlated with EE, yielding a linear fit (R^2^ = 0.59) and Pearson r = 0.77 (*p <* 0.05).

We also evaluated whether incorporating both MD‐derived structural descriptors and sterol‐core chemistry could better capture the experimental variation. In addition to CPP_Rg_ and the mean tilt angle, we included the number of hydroxyl groups on the sterol core, N_OH_, as a sterol‐chemistry descriptor. N_OH_ was assigned according to the bile acid scaffold: cholesterol and LCA‐derived sterols = 1, DCA‐derived sterols = 2, and CA‐derived sterols = 3. The same N_OH_ value was applied to the C4, C16, and C20 esterified derivatives within each sterol family. We then constructed an expanded linear predictor η from standardized CPP_Rg_, θ, and N_OH_, such that higher CPP_Rg_, lower tilt angle, and sterol‐core hydroxylation jointly contributed to η. This expanded predictor improved the correlation with EE, increasing R^2^ from 0.80 to 0.87 (Figure 2N). S_CD_ was excluded because it was strongly correlated with θ and contributed negligible weight in the multivariate fit. The same expanded predictor η accounted for Z‐average with an R^2^ of 0.89, PDI with an R^2^ of 0.66, and Zeta potential with an R^2^ of 0.83 (Figure S18). LNP formulations with larger CPP_Rg_ and smaller tilt angles, as exemplified by CA‐16 and CA‐20 LNPs, scored high and showed higher EE. These results indicate that sterol geometries that enhance tail‐to‐head asymmetry while maintaining interfacial alignment promote encapsulation. Together, these analyses support the combined use of MD‐derived descriptors, including CPP_Rg_ and tilt angle, together with the sterol‐chemistry descriptor N_OH_, to screen sterol chemotypes and prioritize LNP formulations before experimental validation.

Taken together, the MD simulation trajectory analyses indicate that the sterol‐dependent encapsulation performance arises from the interplay between intrinsic sterol polarity, A/B ring geometry, and the compensatory influence of the hydrophobic‐tail structure. Bile acid‐derived sterols with short tails, representing chemotypes in which the polarity of the ester linkage and cis‐fused ring geometry remain dominant, consistently display large tilt angles, reduced acyl‐chain order, and a tendency to accumulate near the bilayer midplane. These configurations reflect weakened interfacial anchoring and diminished packing coherence, consistent with the physicochemical behaviors observed in Figure 2A–C, including low encapsulation efficiencies and broad particle size characteristics. In contrast, long and branched hydrophobic tails (C16 and C20) progressively alleviated polarity‐ and geometry‐driven constraints by stabilizing sterol orientation and enhancing hydrophobic alignment, yielding tilt‐angle distributions and Z‐density profiles that more closely resembled those of cholesterol. This compensatory effect explains why only tail‐extended derivatives recovered cholesterol‐like packing and encapsulation performance, whereas their short‐tailed counterparts remained structurally and functionally limited. Importantly, the consistency among MD‐derived structural descriptors, cryo‐TEM‐observed morphological features, and experimentally measured physicochemical properties further supports the proposed structure–property relationships governing bile acid‐derived sterol LNPs.

Collectively, these findings highlight the utility of MD‐derived descriptors in rationalizing how sterol molecular structure governs experimentally observed LNP behavior. Among these, CPP_Rg_ and sterol tilt angle emerged as robust complementary predictors that together accounted for a substantial fraction of the experimental variation in EE and related physicochemical parameters. In this sterol library, N_OH_ further improved the explanatory power by capturing scaffold‐specific hydroxylation information that was not fully represented by the MD‐derived geometric and orientational descriptors alone. Because they capture geometric and orientational features that are not unique to sterols but are generalizable across diverse lipid classes, these metrics offer a broadly applicable framework for understanding how molecular structure governs interfacial behavior and packing within LNP assemblies. Nevertheless, a broader evaluation across diverse simulation and formulation contexts, including multiple environments, independent replicates, and an expanded chemical space, will be valuable for further assessing the generality of these relationships [35]. Nevertheless, these analyses indicate that optimal LNP performance is achieved when the hydrophobic‐tail design sufficiently counterbalances the sterol core polarity and geometric constraints, positioning CPP_Rg_ and tilt angle as practical simulation‐derived descriptors for early‐stage lipid and rational formulation screening, with principles likely to extend to next‐generation LNP systems incorporating alternative lipid chemotypes.

### 2.6 Encapsulation Efficiency Tracks Splenic Expression, Whereas Hepatic Attenuation is Decoupled From Overall Physicochemical Properties

The organ‐level expression distribution patterns observed for bile acid‐derived sterol LNPs in Figure 1 can be interpreted by integrating sterol structural variables with the structure‐property relationships established in Figure 2. Therefore, we performed correlation analyses between the liver and spleen FLuc expression values (Figure 1D,E) and the physicochemical metrics summarized in Table 1 (Figure 2O,P and Figure S19). Among the evaluated parameters, EE showed the strongest correlation with splenic FLuc expression (R^2^ = 0.71; Figure 2O), emerging as a key formulation‐level metric that accounted for a substantial proportion of spleen‐associated expression trends. In contrast, Z‐average, Zeta potential, and PDI exhibited weaker correlations than EE (Figure S19A). Consistent with EE trends across the bile acid‐derived sterol library, short‐tailed (C4) formulations yielded low splenic expression, whereas the incorporation of long hydrophobic tails (C16 or C20) was associated with increased splenic expression (Figure 1E). These results suggest that across bile acid cores bearing different hydroxyl numbers and positions, achieving a cholesterol‐like functional output requires sufficient hydrophobic compensation. A clear polarity‐hydrophobicity coupling trend was observed between the number of hydroxyl groups and the tail structure. Within the C4 series, increased hydroxylation was associated with a stepwise decrease in expression (LCA‐4 > DCA‐4 > CA‐4), suggesting that under conditions where core polarity remains high and hydrophobic volume/anchoring is insufficient, a reduced EE may translate into attenuated expression. In contrast, within the long‐tail (C16/C20) series, higher hydroxylation correlated with increased expression (CA > DCA > LCA), indicating that when the tail design adequately offsets the core polarity, the restoration of EE and improved expression can be favored, which was also observed in our LNP bilayer simulations. Collectively, these findings suggest that the spleen‐specific structure‐expression relationship likely reflects a composite interplay between core polarity (hydroxyl‐group count) and tail hydrophobicity (length/branching), mediated through EE, rather than a single dominant determinant.

The liver expression patterns showed no systematic correlation with the evaluated physicochemical metrics, with consistently low coefficients of determination across all parameters (EE, R^2^ = 0.11; Z‐average, R^2^ = 0.07; Zeta potential, R^2^ = 0.15; PDI, R^2^ = 0.002) (Figure 2P and Figure S19B). This suggests that the observed hepatic attenuation cannot be readily explained by simple size‐ or surface charge‐driven physicochemical effects. Reduced liver preference was broadly conserved across the bile acid‐derived sterol LNP class and was consistent with the structural comparison summarized in Figure S5. This phenotype may reflect the scaffold‐level structural features shared among bile acid‐derived sterols. To support this interpretation, we performed additional quantitative validation of liver‐associated expression, as described in the following section.

### 2.7 Protein‐Level Validation of Attenuated Hepatic Expression in Bile Acid‐Derived Sterol LNPs

**FIGURE 3:**
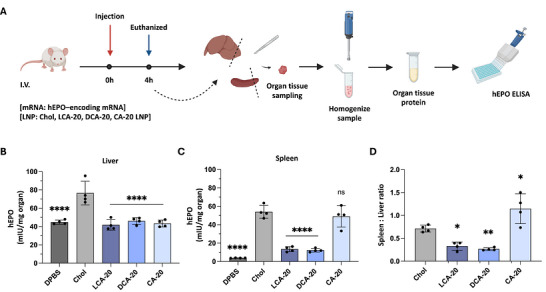
Comparative organ‐level hEPO expression in the liver and spleen across Chol and bile acid‐derived sterol LNPs. (A) Experimental scheme to analyze organ‐level protein expression following intravenous administration of human erythropoietin (hEPO)‐encoding mRNA‐LNPs (0.5 mg kg^−^^1^) in female BALB/c mice (6–7 weeks old, *n* = 4 biologically independent animals per group) administered DPBS, Chol LNPs, or bile acid‐derived sterol LNPs (LCA‐20, DCA‐20, and CA‐20). The liver and spleen were harvested 4 h after injection, and proteins were extracted from equal organ tissue masses for quantification of hEPO protein levels using ELISA. (B,C) Quantification of hEPO protein levels in the liver (B) and spleen (C). hEPO values were normalized to the organ tissue weight and are expressed as mIU per mg of organ. (D) Spleen‐to‐liver ratios of hEPO expression calculated for each LNP group. Data are presented as individual data points with the mean ± SD. Statistical analysis was performed using one‐way ANOVA with Dunnett's multiple comparisons test (^*^ *p <* 0.05, ^**^ *p <* 0.01, ^****^ *p <* 0.0001; ns, not significant), with all comparisons performed relative to the Chol LNP group.

To quantitatively validate the attenuated hepatic expression phenotype observed for bile acid‐derived sterol LNPs, we selected the C20‐tail formulations (LCA‐20, DCA‐20, and CA‐20), which exhibited relatively recovered EE within each bile acid series, as representative candidates. These sterols were formulated with human erythropoietin (hEPO)‐encoding mRNA and administered intravenously, after which liver and spleen tissues were harvested at 4 h for ELISA‐based quantification of hEPO levels (Figure 3A). Consistent with the organ‐level expression trends observed in the FLuc dataset, Chol LNPs produced the highest hEPO levels in the liver, whereas all three bile acid‐derived sterol C20 formulations exhibited markedly reduced hepatic hEPO levels, approaching Dulbecco's phosphate‐buffered saline (DPBS) baseline (Figure 3B). This protein‐level measurement indicated the attenuation of liver‐associated expression across the bile acid‐derived sterol LNP class under matched dosing conditions. Formulation‐dependent differences were also observed in the spleen (Figure 3C). Although LCA‐20 and DCA‐20 showed reduced splenic hEPO levels compared to Chol LNPs, CA‐20 maintained splenic hEPO at a level comparable to that of Chol LNPs. The spleen‐to‐liver ratio was highest for CA‐20 (Figure 3D), reflecting a combined phenotype of strong hepatic attenuation and preserved splenic expression. Based on this coupled phenotype, CA‐20 was prioritized as the lead formulation for subsequent studies.

To further contextualize these tissue‐level findings, we measured serum hEPO levels 4 h postinjection and evaluated their relationship with organ‐level FLuc expression (Figure S20). Serum hEPO levels were markedly reduced in the bile acid‐derived sterol C20 formulations relative to those in Chol LNPs, with approximately 54‐fold (LCA‐20), 33‐fold (DCA‐20), and 30‐fold (CA‐20) lower concentrations (Figure S20A). Across individual animals, serum hEPO levels were strongly correlated with liver FLuc expression (R^2^ = 0.96; Figure S20B), but not with spleen FLuc expression (R^2^ = 0.02; Figure S20C). This pattern agrees with prior observations that systemic hEPO protein levels predominantly reflect the liver's capacity for protein production, rather than expression originating from the spleen [44, 45].

Multiple orthogonal readouts were consistent with the attenuated liver‐associated protein expression for bile acid‐derived sterol LNPs: (i) reduced hepatic hEPO levels quantified directly in the liver tissue (Figure 3), (ii) decreased serum hEPO concentrations that tracked liver FLuc signals across animals (Figure S20), and (iii) liver‐depleted, spleen‐shifted organ‐level FLuc expression profiles in the luciferase dataset (Figure 1). Taken together, these results support the conclusion that liver‐associated expression is attenuated across bile acid‐derived sterol LNPs under matched dosing conditions, with CA‐20 showing the most pronounced shift toward spleen‐centered expression.

### 2.8 ApoE Binding Characteristics in the Protein Corona of Chol and CA‐20 LNPs

**FIGURE 4:**
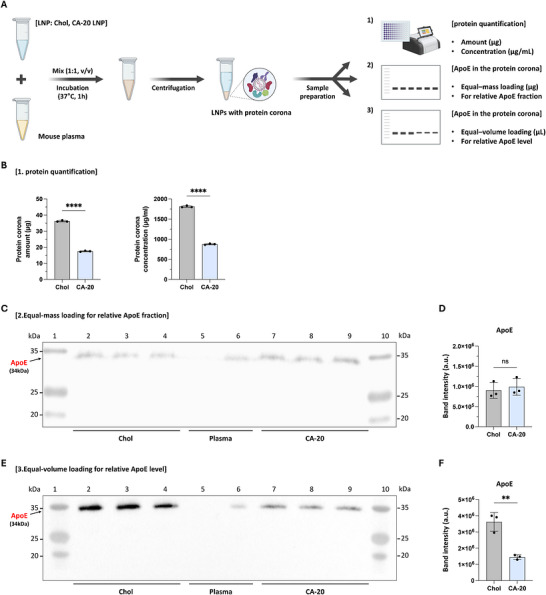
ApoE binding characteristics in the protein coronas of Chol and CA‐20 LNPs. (A) Chol LNPs and CA‐20 LNPs were mixed with mouse plasma to form protein coronas, and the corona‐coated LNPs were collected by centrifugation for downstream analyses (*n* = 3 independent corona preparations per group). The recovered corona samples were used for total protein quantification and ApoE Western blotting under either equal protein‐mass loading or equal sample volume loading conditions. (B) Total protein amount and concentration associated with the LNP protein corona. (C,D) Western blot analysis of ApoE (34 kDa) associated with the LNP protein corona, with equal protein‐mass loading (C), and corresponding band‐intensity quantification (a.u.) (D) relative ApoE fraction in the corona. Lanes 2–4 correspond to Chol LNP coronas (n = 3), and lanes 7–9 correspond to CA‐20 LNP coronas (*n* = 3). Lanes 5 and 6 contain centrifuged plasma (LNP‐free control) and native plasma (baseline reference; no centrifugation or LNP incubation), respectively, and lanes 1 and 10 represent protein ladders. (E,F) ApoE Western blot of corona samples loaded by equal sample volume (E) and corresponding band‐intensity quantification (a.u.) (F), comparing relative ApoE levels between the Chol and CA‐20 LNP groups. Lane assignments are described in (C). Data are presented as individual data points with the mean ± SD. Statistical analysis was performed using an unpaired two‐tailed *t*‐test (^**^ *p <* 0.01, ^****^ *p <* 0.0001; ns, not significant).

To evaluate whether the reduced liver‐associated expression observed for bile acid‐derived sterol LNPs was linked to altered interactions under circulating conditions, we compared the plasma protein corona profiles of Chol and CA‐20 LNPs, particularly focusing on apolipoprotein E (ApoE), which is widely implicated in liver targeting. As shown in Figure 4A, LNPs were incubated with mouse plasma to form protein coronas using independent samples (*n* = 3), and the corona‐coated LNPs were recovered by centrifugation for downstream analyses.

First, the total protein and protein concentrations associated with each LNP corona were quantified. CA‐20 LNPs consistently exhibited lower total corona protein amounts (µg) and concentrations (µg/mL) than Chol LNPs (Figure 4B), indicating that CA‐20 LNPs adsorb fewer plasma proteins overall under matched incubation conditions. Next, ApoE association was assessed by western blotting using two loading strategies designed to distinguish the relative ApoE fraction in the corona from the relative ApoE levels at the sample level. Under equal protein‐mass loading (i.e., reflecting ApoE as a fraction of the total protein corona), the ApoE band patterns near 34 kDa were similar between Chol and CA‐20 LNPs (Figure 4C), and band‐intensity quantification normalized to arbitrary units (a.u.) showed no statistically significant difference between the two formulations (ns) (Figure 4D). This indicates that the relative contribution of ApoE to the protein corona did not differ substantially between the formulations. In contrast, under equal sample volume loading, the ApoE band intensity was reduced for CA‐20 LNPs compared to that for Chol LNPs (Figure 4E). This trend was confirmed by densitometric quantification (Figure 4F), where CA‐20 LNPs exhibited approximately 2.5‐fold lower ApoE association than Chol LNPs (a.u.), and the difference was statistically significant (*p <* 0.01).

Considering the reduced total protein corona burden for CA‐20 LNPs, these data suggest that although CA‐20 LNPs maintain a broadly similar ApoE fraction within the protein corona, the overall extent of ApoE association is reduced. Collectively, these results provide protein‐corona‐level evidence consistent with the attenuated liver‐associated expression phenotype observed for bile acid‐derived sterol LNPs. Specifically, CA‐20 LNPs formed a reduced overall protein corona and exhibited lower ApoE association under matched incubation conditions.

### 2.9 Immunological Evaluation of HA mRNA‐LNPs Formulated With Cholesterol and CA‐20

**FIGURE 5:**
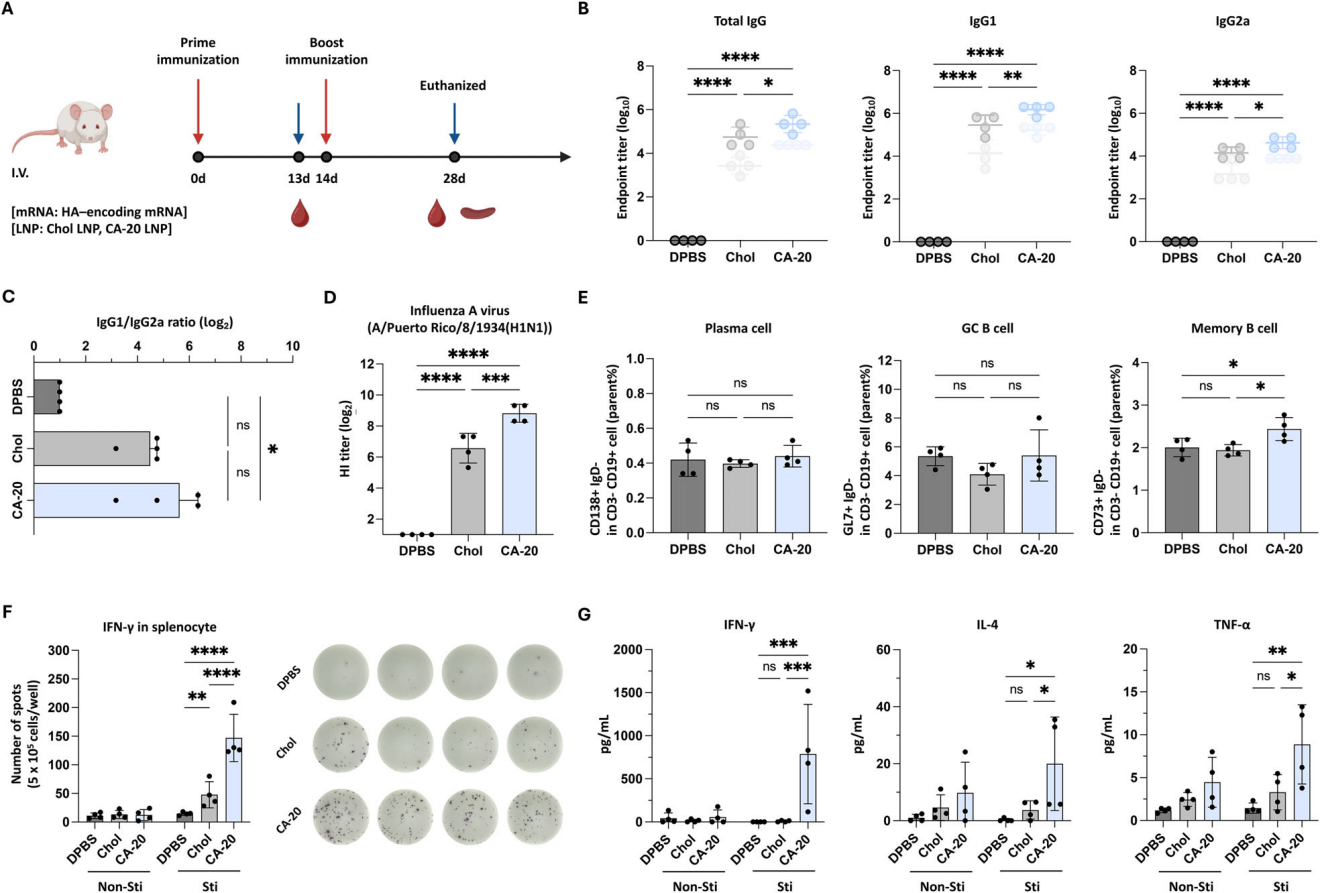
Antigen‐specific immune responses induced by Chol and CA‐20 LNPs. (A) Immunization schedule. BALB/c female mice (6–7 weeks old, *n* = 4 biologically independent animals per group) were intravenously administered DPBS, Chol LNPs, or CA‐20 LNPs encapsulating HA‐encoding mRNA at a dose of 0.5 mg kg^−^^1^. Mice that received mRNA LNPs were primed and boosted 2 weeks later. Serum was collected on day 13 (post‐prime) and day 28 (post‐boost), and spleens were harvested on day 28. (B) Serum antibody titers (total IgG, IgG1, and IgG2a) were measured using endpoint ELISA. Light‐colored dots correspond to titers obtained after the prime immunization (day 13), whereas dark‐colored dots correspond to titers obtained after the boost immunization (day 28). (C) IgG1/IgG2a ratio indicating the Th2/Th1 balance. (D) Hemagglutination‐inhibition (HI) titers against influenza A/Puerto Rico/8/1934 (H1N1) as a measure of antigen‐specific neutralizing antibody responses. (E) Flow cytometry quantification of splenic plasma cells (CD138^+^ IgD^−^), GC B cells (GL7^+^ IgD^−^), and memory B cells (CD73^+^ IgD^−^) gated within CD3^−^ CD19^+^ B cells. (F,G) Antigen‐specific cellular immune responses: IFN‐γ‐secreting splenocytes measured by ELISPOT following antigenic peptide stimulation (Sti) or non‐stimulation (Non‐Sti) (F), and cytokine levels (IFN‐γ, IL‐4, TNF‐α) in splenocyte culture supernatants measured by ELISA under the same conditions (G). Data are presented as individual data points with mean ± SD. Statistical analyses were performed using one‐way ANOVA followed by Fisher's least significant difference (LSD) test (B–D) or one‐way ANOVA with Tukey's multiple comparisons test (E‐G), as indicated (^*^ *p <* 0.05, ^**^ *p <* 0.01, ^***^ *p <* 0.001, ^****^ *p <* 0.0001; ns, not significant).

To determine whether the spleen‐centered expression profile induced by bile acid‐derived sterol substitution translated into enhanced immunogenicity, we evaluated a prime‐boost immunization regimen using hemagglutinin (HA) mRNA‐LNPs formulated with either cholesterol or CA‐20. Mice were intravenously (I.V.) administered DPBS, Chol LNPs, or CA‐20 LNPs (0.5 mg kg^−^^1^), followed by a booster dose 2 weeks later, and both humoral and cellular immune responses were analyzed (Figure 5A). Immunization via the standard vaccination route (I.M.) was also performed (Figure S21).

Among the bile acid‐derived sterol libraries, CA‐20 was selected as the lead formulation based on an integrated assessment of its physicochemical properties, simulation‐based structural analysis, and in vivo expression profiles. CA‐20 exhibited high mRNA EE and low dispersity, maintained stability comparable to that of the Chol LNPs (Figure S22), and showed a relatively ordered lipid arrangement in the MD analyses. In vivo, CA‐20 preserved the overall expression capacity, while markedly attenuating liver‐associated output and redistributing expression toward the spleen, an immune‐relevant organ, supporting its suitability for immunological evaluation.

Serum analyses showed that CA‐20 LNPs elicited strong antigen‐specific antibody responses following IV immunization (Figure 5B). After priming and boosting, Chol and CA‐20 LNPs induced a robust increase in total IgG and IgG subclasses compared to that in DPBS (*p <* 0.001). After the boost, CA‐20 LNPs produced significantly higher endpoint titers than Chol LNPs (total IgG, *p <* 0.05, IgG1, *p <* 0.01, IgG2a, *p <* 0.05), corresponding to approximately 3.4‐fold (total IgG), approximately 4.1‐fold (IgG1), and approximately 3.0‐fold (IgG2a) increases (fold changes calculated based on group mean endpoint titers). These results indicate that the CA‐20 formulation broadly enhanced the magnitude of humoral responses under I.V. conditions. To indirectly assess the Th2/Th1 balance, IgG1/IgG2a ratios were calculated (Figure 5C). CA‐20 LNPs exhibited a significantly higher IgG1/IgG2a ratio than Chol LNPs (*p <* 0.05), suggesting a relative shift toward Th2‐associated humoral features with CA‐20.

Functional antibody quality was evaluated using hemagglutination‐inhibition (HI) assays against H1N1 (Influenza A/Puerto Rico/8/1934) (Figure 5D). CA‐20 LNP‐immunized mice showed significantly higher HI titers than Chol LNP‐immunized mice (*p <* 0.001), reflecting improved neutralizing activity with an approximately 4.4‐fold increase (fold change calculated based on the group mean HI titers). For reference, following I.M. immunization, broadly comparable titers were observed between the two formulations for antigen‐specific total IgG/IgG subclasses and neutralizing responses, as shown in Figure S21B–D.

Flow cytometric analysis of splenocytes collected on day 28 showed no substantial between‐group differences in the frequency of plasma cells (CD138^+^IgD^−^) or GC B cells (GL7^+^IgD^−^) (Figure 5E). In contrast, memory B cells (CD73^+^IgD^−^) were significantly increased in the CA‐20 group compared with the Chol group (*p <* 0.05), suggesting enhanced formation/maintenance of antigen‐experienced memory B cells after I.V. immunization. The magnitude of this increase corresponded to an approximate +0.5% change in the memory B cell frequency. The corresponding I.M. analysis is presented in Figure S21E, showing broadly similar trends.

Antigen‐specific cellular responses were assessed using IFN‐γ enzyme‐linked immunospot (ELISPOT) and cytokine enzyme‐linked immunosorbent assay (ELISA) following peptide stimulation. In the IFN‐γ ELISPOT assay, CA‐20 LNPs induced a markedly higher number of IFN‐γ‐secreting cells than Chol LNPs under stimulation conditions (*p <* 0.0001), indicating enhanced cellular immune responses after intravenous immunization (Figure 5F). Cytokine measurements further supported this pattern. CA‐20 LNPs produced significantly higher IFN‐γ (*p <* 0.001) and increased IL‐4 and TNF‐α (*p <* 0.05) levels relative to Chol LNPs, suggesting broader activation of antigen‐responsive cytokine production (Figure 5G). The corresponding I.M. analysis is presented in Figure S21F,G. Although the Chol LNPs elicited higher cellular responses under I.M. conditions, CA‐20 LNPs still induced statistically significant cellular immunity relative to that of DPBS (*p <* 0.0001).

Taken together, CA‐20 LNPs enhanced the magnitude and functional quality of HA‐specific humoral immunity, increased memory B cell formation, and elicited more robust antigen‐specific cellular immune responses than Chol LNPs. These immune outcomes were aligned with the spleen‐centered expression phenotype observed in vivo for CA‐20 LNPs, which follows the concept that sterol substitution can modulate organ‐level expression patterns associated with enhanced immunogenicity. However, the enhanced immune activity observed in this study reflects immunogenicity alone, and the extent to which these immune signatures translate into or correlate with in vivo safety remains to be determined.

### 2.10 Acute Biochemical and Histopathological Safety Evaluation of Chol and CA‐20 LNPs

**FIGURE 6:**
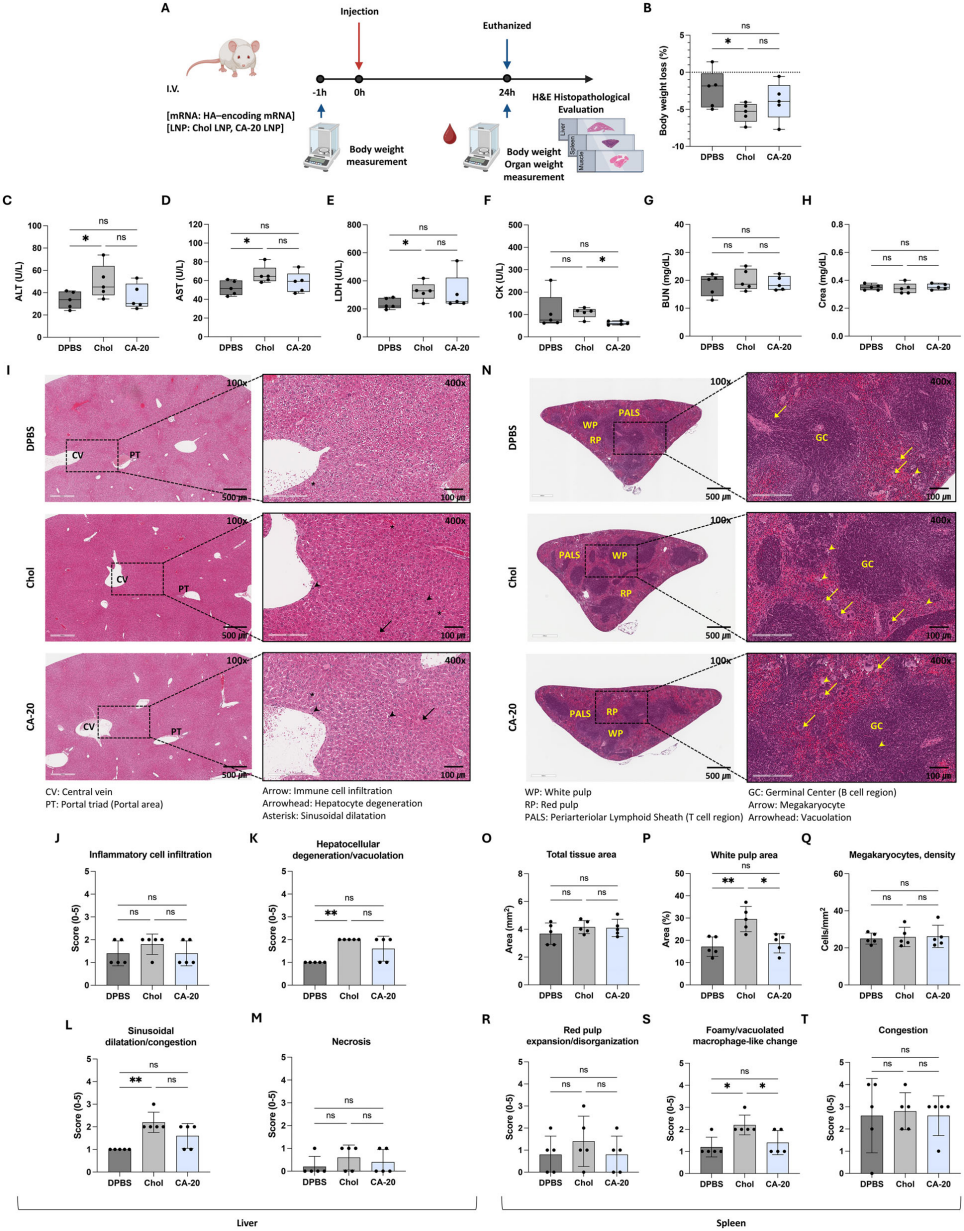
Acute safety evaluation of Chol and CA‐20 LNPs. (A) Experimental timeline for toxicity evaluation. ICR female mice (6–7 weeks old, *n* = 5 biologically independent animals per group) were intravenously administered DPBS, Chol LNPs, or CA‐20 LNPs encapsulating HA‐encoding mRNA at a dose of 0.5 mg kg^−^^1^. Body weight was measured 1 h before administration and 24 h post‐administration. At 24 h post‐injection, mice were euthanized, and blood and tissues were collected for serum biochemistry and histopathological analyses. (B) Body weight loss. Serum biochemical parameters including (C) alanine aminotransferase (ALT), (D) aspartate aminotransferase (AST), (E) lactate dehydrogenase (LDH), (F) creatine kinase (CK), (G) blood urea nitrogen (BUN), and (H) creatinine (Crea). (I) Representative H&E‐stained liver sections (100× and 400× magnification). CV, central vein; PT, portal triad. Arrows indicate inflammatory cell infiltration, arrowheads indicate hepatocellular degeneration, and asterisks indicate sinusoidal dilatation. Semi‐quantitative histopathological evaluation of liver sections including (J) inflammatory cell infiltration, (K) hepatocellular degeneration/vacuolation, (L) sinusoidal dilatation/congestion, and (M) necrosis. (N) Representative H&E‐stained spleen sections (100× and 400× magnification). WP, white pulp; RP, red pulp; PALS, periarteriolar lymphoid sheath; GC, germinal center. Arrows indicate megakaryocytes, and arrowheads indicate vacuolation. Quantitative and semi‐quantitative histopathological evaluation of spleen sections including (O) total tissue area, (P) white pulp area, (Q) megakaryocyte density, (R) red pulp expansion/disorganization, (S) foamy/vacuolated macrophage‐like change, and (T) congestion. Data are presented with individual data points. Body weight and serum biochemistry data (B–H) are shown as box‐and‐whisker plots, whereas histopathological analyses (J–M, O–T) are presented as mean ± SD. Statistical significance was assessed using the Kruskal–Wallis test followed by Dunn's multiple‐comparisons test (^*^ *p* < 0.05, ^**^ *p* < 0.01; ns, not significant).

To evaluate the safety of CA‐20 LNPs, which exhibited reduced hepatic expression, spleen‐selective biodistribution, and enhanced immunogenicity, an acute safety assessment was performed using outbred ICR mice (6–7 weeks old, *n* = 5 per group). DPBS, Chol LNPs, or CA‐20 LNPs (0.5 mg kg^−^^1^) were administered intravenously, and body weight changes and serum biochemical markers associated with hepatic injury, tissue/muscle injury, and renal function were quantitatively analyzed 24 h post‐injection (Figure 6A). Furthermore, gross organ morphology, organ weights, and hematoxylin and eosin (H&E)‐based histopathological evaluations were performed.

At 24 h post‐administration, the Chol LNP group exhibited significant body weight loss compared with the DPBS group, whereas the CA‐20 LNP group showed no significant difference relative to DPBS (Figure 6B). Hepatocellular injury was assessed by measuring serum alanine aminotransferase (ALT) and aspartate aminotransferase (AST) levels (Figure 6C,D) [46]. Chol LNPs significantly increased ALT and AST levels compared with DPBS, whereas CA‐20 LNPs did not induce significant elevations relative to DPBS. These findings are consistent with the reduced hepatic expression profile of CA‐20 LNPs and suggest reduced liver‐associated stress. Tissue and muscle injury were evaluated using lactate dehydrogenase (LDH) and creatine kinase (CK) [47]. Chol LNPs significantly increased LDH levels compared with DPBS, whereas CA‐20 LNPs maintained LDH levels comparable to those of DPBS (Figure 6E). Moreover, CK levels were significantly lower in the CA‐20 LNP group than in the Chol LNP group, indicating a more favorable safety profile with respect to tissue and muscle injury (Figure 6F). Renal function was assessed by measuring blood urea nitrogen (BUN) and creatinine levels (Figure 6G,H) [48]. Both parameters remained within the normal physiological range, with no significant differences among the groups, indicating that CA‐20 LNPs did not measurably affect renal filtration capacity.

To further evaluate tissue‐level safety, gross organ morphology, organ weights, and H&E‐based histopathological analyses were performed 24 h after administration. No overt abnormalities were observed in the gross appearance of the liver or spleen, and body weight‐normalized liver and spleen weights showed no significant adverse changes following CA‐20 LNP administration (Figure S23). Histopathological evaluation was subsequently performed on liver and spleen tissues collected following intravenous administration (Figure 6I–T). In liver sections, Chol LNP‐treated mice showed significantly increased hepatocellular degeneration/vacuolation and sinusoidal dilatation/congestion compared with DPBS controls, whereas CA‐20 LNP‐treated mice maintained both parameters at levels comparable to those of DPBS controls. Inflammatory cell infiltration and necrosis also remained similar to DPBS levels in the CA‐20 LNP group (Figure 6I–M). In spleen sections, Chol LNP treatment resulted in a significant increase in white pulp area and showed a tendency toward increased foamy/vacuolated macrophage‐like changes. In contrast, the CA‐20 LNP group‐maintained levels comparable to those of DPBS controls across most spleen histopathological parameters, including white pulp area (Figure 6N–T). These findings suggest that the increased splenic accumulation of CA‐20 LNPs is not accompanied by increased histopathological responses in the spleen.

The toxicity evaluation following I.M. administration is shown in Figure S24. Consistent with the intravenous findings, Chol LNPs increased hepatic injury markers, including ALT and AST, whereas CA‐20 LNPs maintained levels comparable to those of DPBS. Furthermore, CA‐20 LNPs exhibited a more stable serum biochemical profile across tissue/muscle injury and renal function parameters. H&E‐based histopathological evaluation likewise showed no evidence of exacerbated tissue responses in the CA‐20 LNP group relative to Chol LNPs. Consistent with these findings, no abnormal gross observations or significant organ weight changes were detected following I.M. administration (Figure S25). In addition, H&E analysis of skeletal muscle tissues collected from the injection site demonstrated that CA‐20 LNPs induced less pronounced myofiber degeneration, interstitial edema, and inflammatory cell infiltration than Chol LNPs, whereas myofiber necrosis remained comparable among groups (Figure S26).

Collectively, while Chol LNPs induced changes in body weight, selected serum biochemical markers, and histopathological parameters, CA‐20 LNPs maintained serum biochemical profiles comparable to those of DPBS and exhibited lower or comparable tissue‐level responses relative to both Chol LNPs and DPBS controls. However, the present study is limited to an acute safety assessment conducted 24 h after a single administration and therefore does not address potential long‐term or repeat‐dose toxicities. Additional studies involving repeated administration and long‐term follow‐up will be required to further establish the safety profile of CA‐20 LNPs. Nevertheless, the current findings suggest that CA‐20 LNPs maintain reduced hepatic expression, spleen‐selective biodistribution, and enhanced immune responses while exhibiting a more favorable acute biochemical and histopathological safety profile than Chol LNPs.

## 3 Conclusion

We posit that sterol engineering is a key, yet relatively underutilized, design axis in mRNA‐LNP development, demonstrating that the modulation of sterol structure can influence LNP physicochemical properties, in vivo expression patterns, and immune function. We constructed and compared a library of nine bile acid‐derived sterols with distinct hydroxyl‐group patterns and alkyl tail lengths. Through a structure‐performance framework, we revealed that sterol structure is a major molecular determinant governing encapsulation performance, particle characteristics, and organ‐level expression outcomes.

Mechanistically, we integrated physicochemical measurements with all‐atom molecular dynamics (MD) simulations and found that the experimentally observed formulation behaviors were aligned with the simulation‐derived structural descriptors. Composite metrics, such as CPP_Rg_ (a lipid shape parameter) and sterol tilt angle, meaningfully explain the variability in EE and related physicochemical properties, supporting the concept that sterol‐driven membrane organization can provide a structural basis for formulation‐level outcomes. These results suggest that MD can serve as a post hoc interpretive tool and as a practical framework for rational sterol screening and candidate narrowing.

Substituting cholesterol with bile acid‐derived sterols consistently attenuated liver‐associated expression and promoted redistribution toward spleen‐centered expression, indicating that the sterol structure can exert a substantial influence on the in vivo fate of LNPs. CA‐20 LNPs linked this redistribution of expression to immunologically favorable outcomes by functionally enhancing antigen‐specific humoral immunity and eliciting antigen‐specific cellular immune responses, including improved memory‐associated immune features. Acute biochemical and histopathological assessments further indicated a more favorable safety profile than that of Chol LNPs under the conditions tested, suggesting meaningful potential in terms of the efficacy–safety balance.

Although ApoE‐focused corona findings provide an important clue for sterol‐dependent changes in surface‐protein interactions, future LC‐MS/MS‐based corona profiling is needed to comprehensively define the corona network and refine the causal links to hepatic targeting and redistribution. In addition, sterol substitution alone may face structural constraints in actively driving specific organs beyond the spleen and injection site (muscle). Accordingly, a promising approach is to use sterol substitution as a hepatic off‐target mitigation module to reduce nonspecific hepatic exposure and expand integrated designs by combining this approach with organ‐selective ionizable lipid engineering and/or ligand‐based strategies (peptides/antibodies).

We established that replacing cholesterol with bile acid‐derived sterols is sufficient to modulate LNP membrane organization, protein corona formation, organ‐level expression patterns, and immune function. By presenting an integrated experimental‐computational workflow that connects sterol structure, membrane organization, and biological performance, we provide a structure‐guided blueprint for developing next‐generation mRNA‐LNP platforms aimed at lymphoid tissues and broader extrahepatic targets beyond liver‐centric delivery paradigms.

## 4 Experimental Section

### 4.1 Synthesis of Bile Acid‐Derived Sterols

Bile acid‐derived sterols were synthesized from three‐bile acids, lithocholic acid (LCA), deoxycholic acid (DCA), and cholic acid (CA), which were purchased from Sigma‐Aldrich (St. Louis, MO, USA). The compounds were synthesized by alkylating the carboxylic acid group using three alkanols, namely 1‐butanol (C4), 2‐hexyl‐1‐decanol (C16), and 2‐octyl‐1‐dodecanol (C20), to systematically modulate the alkyl chain length. The resulting bile acid‐derived sterols (LCA‐4, LCA‐16, and LCA‐20; DCA‐4, DCA‐16, and DCA‐20; CA‐4, CA‐16, and CA‐20) were purified and obtained as final products. Detailed synthetic procedures, together with spectroscopic characterization data (^1^H NMR, ^1^ ^3^C NMR, HRMS), are available in the Supplementary Methods and Figures S27–S53.

### 4.2 Preparation of mRNA

A cap‐dependent RNA expression template (CUK3‐1) containing a 5′ untranslated region (UTR), a multiple‐cloning site within the open reading frame (ORF), a 3′ untranslated region (UTR), and a poly(A) tail was used as previously described [49]. Plasmid DNA was linearized using NotI (Enzynomics, Daejeon, Republic of Korea). In vitro transcription (IVT) was performed using the EZ T7 High‐Yield In Vitro Transcription Kit (Enzynomics, Daejeon, Republic of Korea). Co‐transcriptional capping was performed using SC101 (ST Pharm, Siheung, Republic of Korea), and N1‐methyl‐pseudouridine (CATUG, Basel, Switzerland) was used as a substitute for uridine triphosphate (UTP). The IVT reactions were incubated at 37°C overnight. Residual DNA was removed by treatment with Deoxyribonuclease I (Enzynomics, Daejeon, Republic of Korea) at 37°C for 30 min. RNA was precipitated by adding LiCl and incubating at −20°C for 30 min, followed by centrifugation (13,000 × g, 4°C for 15 min). The pellets were washed with 70% ethanol (in DI water) and centrifuged under the same conditions. The RNA was resuspended in sterile nuclease‐free water. A cellulose‐based secondary purification step was performed to minimize contamination with double‐stranded RNA. RNA concentration and purity were determined using a NanoDrop 2000 spectrophotometer (Thermo Fisher Scientific, Waltham, MA, USA). Purified RNA was aliquoted and stored at −80°C until use. The coding sequences used in this study were Firefly luciferase (FLuc), enhanced green fluorescent protein (eGFP), human erythropoietin (hEPO), and hemagglutinin (HA) inserted into the CUK3‐1 ORF.

### 4.3 Formulation of mRNA‐LNPs

Lipid nanoparticles (LNPs) were formulated using an ionizable lipid (SM‐102 or DLin‐MC3‐DMA; Hanmi Fine Chemical Co., Siheung, Republic of Korea), a helper lipid (DSPC; Avanti Polar Lipids, Alabaster, AL, USA), structural lipids including cholesterol (Avanti Polar Lipids, Alabaster, AL, USA), bile acids, or bile acid‐derived sterols, and a PEG lipid (DMG‐PEG2000; Avanti Polar Lipids, Alabaster, AL, USA). To ensure homogeneous pre‐mixing and complete solubilization of structurally diverse sterol components prior to formulation, the lipid components were first dissolved in a chloroform/methanol mixture (1:1, v/v) at a concentration of 50 µg/µL, mixed according to the molar ratio of ionizable lipid: DSPC: sterol: DMG‐PEG2000 = 50:10:38.5:1.5, and concentrated under reduced pressure to form a thin lipid film. Each lipid film was subsequently re‐dissolved in 100% ethanol to prepare the organic lipid phase. mRNA was diluted in 10.8 mM sodium citrate buffer (pH 4.0) at an N/P ratio of 6 and combined with the lipid solution at a 3:1 (v/v) aqueous‐to‐organic ratio using an Encell Master microfluidic mixer (Enparticle, Busan, Republic of Korea). The resulting LNP formulations were dialyzed overnight in 1× Dulbecco's phosphate‐buffered saline (DPBS; Cell Nest, Yongin, Republic of Korea) using Slide‐A‐Lyzer G3 Dialysis Cassettes, 10 K MWCO (Thermo Fisher Scientific, Waltham, MA, USA), followed by concentration using an Amicon Ultra‐15 centrifugal filter unit (Merck, Darmstadt, Germany). All LNPs were stored at 4°C until use. LNPs containing cholesterol as the structural lipid were prepared under identical conditions and served as control formulations for comparison with the bile acid‐derived sterol LNPs. Both SM‐102‐ and MC3‐based compositions were evaluated to assess the impact of ionizable lipid species on the formulation performance.

### 4.4 Measurement of Encapsulation Efficiency in mRNA‐LNPs

Encapsulation efficiency (EE) was quantified using the Quanti‐iT RiboGreen RNA Assay Kit (Invitrogen, Thermo Fisher Scientific, Waltham, MA, USA) [50]. For each sample, external and total RNA fractions were prepared: external RNA was diluted in 1× TE buffer (Invitrogen, Thermo Fisher Scientific, Waltham, MA, USA), and total RNA was diluted in 0.5% Triton X‐100 in TE buffer (Thermo Fisher Scientific, Waltham, MA, USA). Both samples were diluted 1:1,000, mixed with RiboGreen reagent, and transferred to black 96‐well plates (Corning, NY, USA). Fluorescence intensity was measured using a GloMax Explorer microplate reader (Promega, Madison, WI, USA) at a 500–550 nm wavelength. RNA concentration was determined from a standard curve (top concentration: 1 µg/mL, 2‐fold serial dilutions), and EE (%) was calculated using the following equation:

$$EE\left(\%\right)=\left(\frac{\left(RNA_{Total}-RNA_{free}\right)}{RNA_{Total}}\right)\times 100$$

### 4.5 Dynamic Light Scattering Measurement of mRNA‐LNPs

LNP samples were diluted 100‐fold in the appropriate buffer before measurement. Z‐average hydrodynamic diameter and polydispersity index (PDI) were measured in 1× Dulbecco's phosphate‐buffered saline (DPBS; Cell Nest, Yongin, Republic of Korea), and Zeta potential was measured in deionized water. Measurements were conducted using a Zetasizer Nano ZS (Malvern, Worcestershire, UK) equipped with 12 mm square polystyrene cuvettes (DTS0012 for Z‐average and PDI) and folded capillary Zeta cells (DTS1070 for Zeta potential). All measurements were performed at 25°C after an equilibration period of 120 s.

### 4.6 All‐Atom Molecular Dynamics Simulations

All bilayer systems were constructed using the MolCube Membrane Builder, which is analogous to the CHARMM‐GUI Membrane Builder [51, 52, 53]. Each leaflet contained 98 lipids and was solvated with TIP3P water and 0.15 M NaCl [54]. For each composition, two conditions were simulated: a neutral condition with all ionizable lipids neutral and a protonated condition with a 50:50 mixture of neutral and protonated ionizable lipids. As shown in Figure S7, these conditions approximate neutral pH before intracellular uptake and an early endosome environment (pH approximately 6.5), where approximately 50% of ionizable lipids are protonated, given their pKa ≈ 6.5 [55]. PEGylated lipids were excluded because PEG chains are typically shed during intracellular uptake, and their exclusion reduces the system size and thus improves the sampling efficiency with no differences in the simulation observables [35, 56]. Table S4 summarizes the information for each system, such as the system name, lipid counts per leaflet, water, ion, total atom numbers, system size, and simulation time.

### 4.7 Compositions

SM‐102 served as the ionizable lipid, and DSPC served as the helper lipid. Sterol identity varied among cholesterol (Chol) and nine bile acid‐derived sterols.

### 4.8 Force Fields and Parameterization

All simulations were performed using the CHARMM additive framework (C36 lipids) [57, 58]. Chol, LCA, DCA, and CA were assigned the standard CHARMM sterol parameters. The tail variants (LCA‑4/16/20, DCA‑4/16/20, and CA‑4/16/20) were parameterized by analogy mapping to the CHARMM fragments [59]. DSPC used the standard CHARMM lipid definition, and SM‑102 force‐field parameters were obtained from LNPDB [35].

### 4.9 Equilibration and Production of Molecular Dynamics Simulations

We followed the six‐step equilibration procedure used in the CHARMM‐GUI Membrane Builder protocol at 310 K, gradually reducing the positional and dihedral restraints on the lipids and sterols during equilibration [51, 52]. Production simulations were conducted using OpenMM in the NPT (constant number of particles, pressure, temperature) ensemble (310 K, 1 bar) for 1.5 µs per system [60], with no restraints. A 4‐fs time step was used for hydrogen mass repartitioning (HMR) [61, 62]. The temperature was controlled using a Langevin thermostat (collision frequency 1 ps^−^^1^) and the pressure using a semi‑isotropic Monte Carlo barostat (coupling interval 0.4 ps) [63]. The bonds to the hydrogen atoms were constrained using SHAKE [64]. The Lennard‑Jones interactions used a 10–12 Å force‑switch scheme, and the long‑range electrostatics used the Particle‐mesh Ewald method with a 12 Å real‐space cutoff [65]. Each system had three replicates starting with different initial velocities, which improved the sampling and trajectory analysis statistics.

### 4.10 Trajectory Preparation for Analysis

The final 500 ns of each trajectory were used for all the analyses. Each frame was recentered along the Z‐axis by placing the instantaneous bilayer midplane at Z = 0. All reported values for each system are the averages and standard deviations (SD) across independent replicates.

### 4.11 Density Profiles

We computed the one‐dimensional mass density (Z‐density) profiles along the Z‐axis (i.e., membrane normal). For each molecular species, a single representative atom was used to track the Z‐position over time: hydroxyl oxygen for sterols, phosphorus for DSPC, and headgroup nitrogen for SM‐102. The representative atom Z‐positions were binned along the Z‐axis with a fixed bin width, and the histograms were normalized by the bin width and time‐averaged to yield smooth continuous profiles that report the vertical organization of each component within the bilayer.

### 4.12 Sterol Tilt Angle

The principal axis of the sterol ring was defined as the vector from C3 (ring hydroxyl‐bearing carbon) to C17 (side‐chain junction carbon) (Figure 2J). For each frame and sterol, the tilt angle was computed as the angle between the principal axis and the Z‐axis. The angles from all sterols and frames were accumulated to obtain the per‐system distributions. The mean tilt angle was defined as the time and ensemble averages, with the values summarized in Figure 2K and Table S3.

### 4.13 Lipid Order Parameter (*S_CD_*)

The acyl‐chain carbon deuterium order parameters (*S*_CD_) were computed as follows:

$$S_{\mathrm{CD}}=\left|\frac{\langle 3\cos^{2}\theta -1\rangle }{2}\right|$$

where θ is the angle between each C‐H bond unit vector and the bilayer normal (ẑ), and ⟨·⟩ denotes time and ensemble averaging. For each acyl‐chain carbon, bonded hydrogens were identified, unit vectors **u**_CH_ were formed to calculate *S*_CD_, and per‐bond values were averaged over hydrogens to obtain a per‐carbon *S*_CD_. From each per‐carbon profile, the chain value was taken as the average of the three peak S_CD_ carbon values. We report *S*_CD_ in the *sn*‐1 chain only because DSPC carries two chemically identical saturated stearoyl chains (*sn*‐1/*sn*‐2).

### 4.14 Computing the Critical Packing Parameter Using Radii of Gyration (CPP_Rg_)

We also quantified the CPP for each sterol using an approach based on the radii of gyration (CPP_Rg_). Atoms for each sterol and frame were divided into head and tail groups. We computed the mass‐weighted centers of mass for each set, $R_{\mathrm{COM}}^{\mathrm{head}}$ and $R_{\mathrm{COM}}^{\mathrm{tail}}$, and recentered the sterol coordinates by subtracting the midpoint, $R_{\mathrm{COM}}^{\mathrm{mid}}$.

$$R_{\mathrm{COM}}^{\mathrm{mid}}=\frac{1}{2}\;\left(R_{\mathrm{COM}}^{\mathrm{head}}+R_{\mathrm{COM}}^{\mathrm{tail}}\right)$$

The sterol axis is defined as $v=R_{\mathrm{COM}}^{\mathrm{head}}\;-R_{\mathrm{COM}}^{\mathrm{tail}}$. We then rigidly rotated the sterol to align **v** with the bilayer normal $\hat{z}=\left[0,0,1\right]$. With all sterols consistently oriented, the planar (xy) radii of gyration were evaluated separately for the head and tail atoms.

$$R_{g}\left(S\right)=\sqrt{\frac{1}{M_{S}}\sum_{i\in S}m_{i}\left(x_{i}^{2}+y_{i}^{2}\right)},S\in \left(\mathrm{head},\;\mathrm{tail}\right)$$

where $M_{S}=\sum_{i\in S}m_{i}\;$. A frame‐wise packing ratio was defined as

$${\mathrm{CPP}}_{\mathrm{Rg}}=\frac{R_{g}^{\mathrm{tail}}}{R_{g}^{\mathrm{head}}}\;$$

CPP_Rg_ values > 1 indicate an inverted cone shape, whereas CPP_Rg_ values < 1 indicate a cone shape.

### 4.15 Cryo‐Transmission Electron Microscopy (Cryo‐TEM) Characterization and Morphological Quantification of LNPs

The morphological characteristics of the LNPs were evaluated using cryo‐TEM. Cryogenic samples were prepared by loading a 3 µL aliquot of the LNP solution onto a glow‐discharged Quantifoil grid (Quantifoil R 1.2/1.3 copper 300 mesh, Ted Pella Inc., USA), which was treated at 15 mA for 60 s to enhance hydrophilicity. The grid was loaded into the Vitrobot Mark IV (Thermo Fisher Scientific, Waltham, MA, USA), with the sample chamber maintained at 15°C and 100% humidity. After blotting to remove excess liquid, the specimen was rapidly plunge frozen in liquid ethane and stored in liquid nitrogen before cryo‐TEM imaging. Cryo‐TEM imaging was performed using 200 kV Cryo‐TEM (Thermo Fisher Scientific, Waltham, MA, USA). One hundred images were acquired for each sample condition. Morphological irregularity was evaluated by counting the number of LNPs exhibiting normal spherical morphologies and those displaying irregular morphologies, including surface protrusions, non‐spherical morphologies, emulsion‐like structures, and bleb‐like structures. The morphological irregularity ratio was calculated based on the total number of LNPs analyzed with five randomly selected images per condition. Moreover, the proportion of the bilayer structure within a single LNP was quantified by measuring the length of the visible lipid bilayer region relative to the length of the total surface of the LNP, using ImageJ software version 1.54 (National Institutes of Health, Bethesda, MD, USA).

### 4.16 Animal Experiments

All animal procedures were approved by the Institutional Animal Care and Use Committee (IACUC) of the Catholic University of Korea (CUK‐IACUC‐2024‐032‐02 and CUK‐IACUC‐2024‐033‐02). Female BALB/c and ICR (CD‐1) mice (6–7 weeks old) were purchased from Koatech (Gyeonggi‐do, Republic of Korea) and housed under specific pathogen‐free (SPF) conditions at the Catholic University Animal Facility under a 12 h light/dark cycle. BALB/c mice were used for biodistribution studies using FLuc‐encoding mRNA‐LNPs and enhanced eGFP‐encoding mRNA‐LNPs, tissue and serum quantification of hEPO‐encoding mRNA‐LNPs, and immunization studies using HA‐encoding mRNA‐LNPs. ICR (CD‐1) mice were used for toxicological evaluation. All animal experiments were conducted in accordance with institutional guidelines and approved protocols.

### 4.17 Firefly Luciferase (FLuc) Assay for In Vivo Biodistribution Analysis

LNP formulations were prepared by encapsulating FLuc‐encoding mRNA. BALB/c mice were administered FLuc‐encoding mRNA‐LNPs via intravenous (I.V.) or intramuscular (I.M.) injection at an mRNA dose of 0.5 mg kg^−^^1^. For the I.V.‐injected groups, mice were analyzed 4 h post‐injection, whereas for the I.M.‐injected groups, analysis was performed 6 h post injection. Immediately before imaging, the mice were intraperitoneally (I.P.) injected with 100 µL of luciferin (Promega, Madison, WI, USA) solution prepared at 3 mg/mL in saline. After 5 min, the mice were euthanized, and the spleen, liver, lymph nodes, kidneys, heart, lungs, and injection sites (I.M.) were collected. Bioluminescence signals representing mRNA expression were recorded using an IVIS Spectrum imaging system (LUCI, CELLGENTEK, Republic of Korea), and quantitative image analysis was performed using Living Image software (NEOimage program). Total flux was used for organ‐specific comparisons (liver and spleen), whereas average radiance was used to compute the fractional distribution and spleen‐to‐liver ratios to minimize ROI/organ size effects.

### 4.18 Flow Cytometric Analysis of Enhanced Green Fluorescent Protein (eGFP) Expression in Splenocytes

eGFP‐encoding mRNA‐LNPs were I.V. administered to female BALB/c mice at a dose of 0.5 mg kg^−^^1^. At 4 h post‐injection, mice were euthanized, and spleens were collected for flow cytometric analysis. Splenocytes were isolated according to the procedure described in the Splenocyte Isolation and Preparation section. Splenocytes (1 × 10^6^ cells per well) were seeded into 96‐well U‐bottom plates. Cells were incubated with anti‐mouse CD16/32 antibody (BioLegend, San Diego, CA, USA) for 30 min at 4°C to block Fc receptors. Cell viability was assessed using a LIVE/DEAD Fixable Near‐IR Dead Cell Stain Kit (Thermo Fisher Scientific, Waltham, MA, USA). Surface staining was performed using fluorescently labeled antibodies at 0.1 µg per well for 30 min at 4°C in the dark. Splenocytes were stained with antibodies against CD45, CD3, CD19, CD11b, CD11c, and F4/80. Immune‐cell populations were defined as follows: B cells (CD45^+^CD19^+^), T cells (CD45^+^CD3^+^), dendritic cells (CD45^+^CD11c^+^CD11b^−^), and macrophages (CD45^+^CD11b^+^F4/80^+^). Cells were fixed with 4% paraformaldehyde. Flow cytometry data were acquired using a CytoFLEX flow cytometer (Beckman Coulter, Brea, CA, USA) and analyzed using CytExpert software (Beckman Coulter, Brea, CA, USA).

### 4.19 Organ Tissue Protein Extraction and Enzyme‐Linked Immunosorbent Assay for Human Erythropoietin (hEPO) Quantification

hEPO‐encoding mRNA‐LNPs were intravenously administered to female BALB/c mice at a dose of 0.5 mg kg^−^^1^. At 4 h post‐injection, the mice were euthanized, and liver and spleen tissues were harvested. The excised tissues were immediately snap‐frozen in liquid nitrogen and stored at −80°C until processing. For protein extraction, frozen tissues were weighed, and equal tissue masses (5 mg per sample) were transferred to round‐bottom tubes. Samples were lysed in 150 µL of RIPA lysis buffer supplemented with NaF, Na_3_VO_4_, β‐glycerophosphate, sodium pyrophosphate, a protease inhibitor cocktail, and PMSF. Tissues were homogenized using an electric homogenizer, followed by incubation on ice at 4°C for 1 h with intermittent vortexing (three times during incubation). Lysates were clarified by centrifugation at 16,000 × *g* for 20 min at 4°C, and the supernatant was transferred to a fresh tube. This clarification step was repeated under the same conditions to remove any residual debris. The final supernatant (100 µL) was collected as an organ tissue protein extract. The hEPO levels in the organ tissue protein extracts were quantified using a human EPO DuoSet ELISA kit (R&D Systems, Minneapolis, MN, USA) according to the manufacturer's instructions.

### 4.20 Serum Analysis for Quantification of Human Erythropoietin (hEPO) Expression

hEPO‐encoding mRNA‐LNPs were administered intravenously to BALB/c mice at a dose of 0.5 mg kg^−^^1^. At 4 h post‐injection, the mice were euthanized, and serum was collected for analysis. Serum hEPO levels were quantified using the human EPO DuoSet ELISA kit (R&D Systems, Minneapolis, MN, USA) according to the manufacturer's instructions.

### 4.21 Isolation of Plasma Protein Corona on LNPs

LNPs were prepared as described above and diluted with 1× Dulbecco's phosphate‐buffered saline (DPBS) to a final lipid concentration of 3 mg/mL. To form the plasma protein corona, equal volumes of LNP and EDTA‐treated mouse plasma (Innovative Research, Novi, MI, USA) were mixed at a 1:1 (v/v) ratio and incubated at 37°C for 1 h. Each condition was prepared in triplicate. To isolate LNP‐associated protein corona complexes, the LNP plasma mixtures were centrifuged at 15,300 × g for 20 min at 4°C. After centrifugation, the supernatant containing unbound plasma proteins was carefully removed and discarded. The resulting LNP pellets were washed three times with 1× DPBS by centrifugation at 15,300 × *g* for 5 min at 4°C to remove loosely bound proteins. After the final wash, the pellets were resuspended in RIPA lysis buffer containing protease inhibitors for protein extraction, and the extracted protein samples were stored at −20°C until further analysis. The total protein concentrations of the isolated protein corona samples were quantified using a Pierce BCA Protein Assay Kit (Thermo Fisher Scientific, Waltham, USA) according to the manufacturer's instructions.

### 4.22 Western Blot Analysis of Apolipoprotein E (ApoE) in LNP‐Associated Protein Corona

Equal masses or volumes of protein extracted from LNP‐associated protein corona samples were resolved by 12% SDS‐PAGE and subsequently transferred onto nitrocellulose membranes. The membranes were blocked with 5% (w/v) skim milk in phosphate‐buffered saline containing 0.1% Tween‐20 (PBST) for 1 h at room temperature to minimize nonspecific binding. After blocking, the membranes were incubated overnight at 4°C with primary antibodies against ApoE (BioLegend, San Diego, CA, USA; 1:1,000 dilution). Following primary antibody incubation, the membranes were washed three times with PBST and incubated with horseradish peroxidase (HRP)‐conjugated anti‐rat IgG secondary antibody (Santa Cruz Biotechnology, Dallas, TX, USA; 1:5,000 dilution) for 1 h at room temperature. Antibody‐bound proteins were detected using a D‐Plus ECL Pico or Femto detection system (Dongin Biotech, Seoul, Republic of Korea) and visualized using a ChemiDoc Touch Imaging System (Bio‐Rad Laboratories, Hercules, CA, USA). Band intensities were quantified using the Image Lab 6.1 software (Bio‐Rad Laboratories, Hercules, CA, USA).

### 4.23 Immunization with Hemagglutinin (HA)‐mRNA LNPs

LNPs were formulated by encapsulating the HA‐encoding mRNA. BALB/c mice were immunized via I.V. or I.M. administration at a dose of 0.5 mg kg^−^^1^, administered twice at 2‐week intervals. Mice were sacrificed two weeks after the second immunization, and the spleen and blood were collected for subsequent immunological analysis.

### 4.24 Enzyme‐Linked Immunosorbent Assay

Antigen‐specific total IgG, IgG1, and IgG2a levels were analyzed in the serum of HA‐immunized mice. A 100 µL coating of HA protein (Influenza A H1N1 (A/Puerto Rico/8/1934) HA (Sino Biological, Beijing, China)) (100 ng/well) was applied to a 96‐well plate (SPL, Pocheon, Republic of Korea), followed by incubation at 4°C for 12 h. Subsequently, three washes were performed using 200 µL phosphate‐buffered saline (PBS) containing 0.05% Tween‐20 (0.05% PBST). Blocking was performed for 1 h at room temperature using a blocking buffer (1% bovine serum albumin in PBS). During this period, the serum was diluted 17,714,700‐fold. The mixture was then incubated at room temperature for 2 h. After washing thrice with 0.05% PBST, HRP‐conjugated anti‐mouse IgG (BETHYL, Montgomery, TX, USA), IgG1 (BETHYL, Montgomery, TX, USA), and IgG2a (Bio‐Rad, Hercules, CA, USA) antibodies were diluted to 1:5,000 and incubated at room temperature for 2 h. Subsequently, the samples were washed three times with 0.05% PBST. The reaction was performed for 2 min using a tetramethylbenzidine substrate (TMB; BioLegend, San Diego, CA, USA). The reaction was stopped by adding 2 N H_2_SO_4_. Optical density (OD) was measured at 450 nm using a GloMax Explorer microplate reader (Promega, Madison, WI, USA).

To quantify cytokine secretion by splenocytes, cells were isolated from immunized mice and seeded in 96‐well plates at a density of 5 × 10^5^ cells per well. Splenocytes were stimulated with a mixture of HA‐specific T cell epitope peptides (IYSTVASSL, LYEKVKSQL, DYEELREQL, SFERFEIFPKE, HNTNGVTAACSH, KLKNSYVNKKGK, NAYVSVVTSNYNRRF, and CPKYVRSAKLRM) at a total concentration of 5 µg per well, followed by incubation for 72 h at 37°C. The cytokine levels of IL‐4, IFN‐γ, and TNF‐α in the culture supernatants were determined using ELISA kits (Invitrogen, Thermo Fisher Scientific, Waltham, MA, USA) according to the manufacturer's instructions. Quantification was performed based on standard curves, and cytokine concentrations were expressed in picograms (pg) per mL of supernatant.

### 4.25 Hemagglutination Inhibition Assay

Mouse serum samples were treated with receptor‐destroying enzyme (Denka Seiken, Tokyo, Japan) at 37°C overnight, followed by heat inactivation at 56°C for 30 min to remove nonspecific inhibitors. The treated sera were serially diluted two‐fold with cold phosphate‐buffered saline (PBS) in 96‐well V‐bottom plates (Corning, NY, USA). A standardized influenza virus suspension (8 HA units per 50 µL) was then added to each well, and the plate was incubated at room temperature for 30 min. Subsequently, 1% (v/v) chicken red blood cells diluted in PBS (Innovative Research, Novi, MI, USA) were added (50 µL per well) and incubated for an additional 30 min at room temperature. Antibody titers were expressed as geometric mean titers and determined as the reciprocal of the highest serum dilution that inhibited hemagglutination in duplicate experiments.

### 4.26 Splenocyte Isolation and Preparation

Splenocytes were digested in serum‐free RPMI 1640 medium containing 1 mg/mL Collagenase D (Sigma‐Aldrich, St. Louis, MO, USA) at 37°C for 15 min. The digested spleen tissue was gently homogenized using a 40 µm cell strainer (SPL, Pocheon, Republic of Korea) and centrifuged at 315 × g for 5 min. To remove red blood cells, the pellet was treated with 1× red blood cell lysis buffer (BioLegend, San Diego, CA, USA) and incubated on ice for 5 min. The reaction was neutralized by adding RPMI 1640 medium supplemented with 10% fetal bovine serum (FBS). After centrifugation under the same conditions, the cell pellet was resuspended in RPMI 1640 medium containing 10% FBS for further analysis.

### 4.27 Flow Cytometry Analysis

Splenocytes (1 × 10^6^ cells per well) were seeded into 96‐well U‐bottom plates and prepared as single‐cell suspensions. Cells were incubated with anti‐mouse CD16/32 antibody (BioLegend, San Diego, CA, USA) for 30 min at 4°C to block Fc receptors. Cell viability was assessed using LIVE/DEAD Fixable Aqua Dead Cell Stain. Surface staining was performed using fluorescently labeled antibodies at 0.1 µg per well for 30 min at 4°C in the dark. Splenocytes were stained with antibodies against CD3, CD19, CD138, IgD, GL7, and CD73. B‐cell subsets were defined as follows: plasma cells (CD3^−^CD19^+^CD138^+^IgD^−^), germinal center (GC) B cells (CD3^−^CD19^+^GL7^+^IgD^−^), and memory B cells (CD3^−^CD19^+^CD73^+^IgD^−^). Cells were fixed with 4% paraformaldehyde. Flow cytometry data were acquired using a CytoFLEX flow cytometer (Beckman Coulter, Brea, CA, USA) and analyzed using CytExpert software (Beckman Coulter, Brea, CA, USA).

### 4.28 Enzyme‐Linked Immunospot Assay

Splenocytes (5 × 10^5^ cells per well) were seeded in 96‐well Multi‐Screen‐IP Filter Plates (Millipore, Burlington, MA, USA) and stimulated with 5 µg/mL of an HA‐specific T cell epitope peptide mixture (IYSTVASSL, LYEKVKSQL, DYEELREQL, SFERFEIFPKE, HNTNGVTAACSH, KLKNSYVNKKGK, NAYVSVVTSNYNRRF, and CPKYVRSAKLRM) for 48 h at 37°C. All peptides were synthesized by Peptron (Daejeon, Republic of Korea). IFN‐γ secretion by T cells was detected using a Mouse IFN‐γ ELISpot BASIC Kit (Mabtech, Stockholm, Sweden) according to the manufacturer's instructions.

### 4.29 Toxicological Analysis

BALB/c mice were administered HA‐encoding mRNA‐LNP formulations via I.M. or I.V. injection at a dose of 0.5 mg kg^−^^1^. At 24 h post‐injection, blood samples were collected, and serum was analyzed by the Korea Pathology Technical Center (KP&T, Cheongju‐si, Republic of Korea). Body weight was recorded 1 h before injection and again 24 h post‐administration to monitor the acute systemic responses. Serum biochemical parameters, including alanine aminotransferase (ALT), aspartate aminotransferase (AST), lactate dehydrogenase (LDH), creatine kinase (CK), blood urea nitrogen (BUN), and creatinine (Crea) levels, were quantified to evaluate hepatic, renal, muscular, and systemic toxicities.

### 4.30 Histopathological Analysis (Hematoxylin and Eosin Staining, H&E)

The spleen, liver, and skeletal muscle were collected according to standard procedures. Before further processing, the excised spleen and liver were gently blotted to remove excess moisture and weighed. Subsequently, liver, spleen, and skeletal muscle tissues were fixed in 4% paraformaldehyde, paraffin‐embedded, and sectioned at a thickness of 4 µm for histopathological analysis. Tissue sections were stained with H&E to evaluate tissue morphology and cellular architecture. Histopathological images were acquired and analyzed using Aperio Image Scope version 12.6 (Leica Biosystems Pathology Imaging, Buffalo Grove, IL, USA). Stained sections were examined and imaged under a light microscope. Histopathological findings were semi‐quantitatively scored on a 0–5 scale according to the severity and extent of lesions as follows: 0, none; 1, minimal; 2, mild; 3, moderate; 4, marked; and 5, severe. Histopathological evaluation was performed using organ‐specific parameters. For liver tissues, inflammatory cell infiltration, hepatocellular degeneration/vacuolation, sinusoidal dilatation/congestion, and necrosis were assessed using the semi‐quantitative scoring system described above. For spleen tissues, white pulp area and megakaryocyte density were quantitatively measured, whereas red pulp expansion/disorganization, congestion, and foamy/vacuolated macrophage‐like changes were evaluated using the semi‐quantitative scoring system. For skeletal muscle tissues, myofiber degeneration, myofiber necrosis, interstitial edema, and inflammatory cell infiltration were assessed using the same semi‐quantitative scoring criteria.

### 4.31 Statistical Analysis

Statistical analyses were performed using GraphPad Prism 10 (GraphPad Software, San Diego, CA, USA). Data are presented as individual data points with mean ± standard deviation (SD) unless otherwise indicated. Statistical tests were selected based on the experimental design, data distribution, and number of groups. One‐way analysis of variance (ANOVA) was used to compare three or more groups. When ANOVA indicated statistical significance, appropriate post hoc tests were used depending on the comparison strategy. Dunnett's multiple comparison test was used for comparisons relative to the single control group (Chol LNPs). Tukey's multiple comparison test was used for pairwise comparisons among groups. Fisher's least significant difference (LSD) test was used for predefined comparisons between selected groups. For direct comparisons between the two groups, an unpaired two‐tailed Student's t‐test was performed. For datasets that did not meet the assumptions of normality or homogeneity of variance, the non‐parametric Kruskal–Wallis test followed by Dunn's multiple‐comparisons test was applied. Statistical significance was defined as follows: ^*^ *p <* 0.05, ^**^ *p <* 0.01, ^***^ *p <* 0.001, ^****^ *p <* 0.0001, and ns, not significant.

To quantitatively assess the relationship between the simulation‐derived structural descriptors and the experimentally measured values, we constructed multivariate linear regression models using standardized input variables. For the regression analysis, CPP_Rg_, tilt angle, S_CD_, and the number of hydroxyl groups on the sterol core (N_OH_) were evaluated. CPP_Rg_, tilt angle, and S_CD_ were obtained from MD simulations, whereas N_OH_ was assigned as a sterol‐chemistry descriptor based on the sterol scaffold. Before the regression analysis, all variables were standardized (z‐score) to remove scale dependence. *S*_CD_ was excluded from the final multivariate model because it was collinear with the tilt angle and contributed negligibly to the model fit. Directionality was imposed such that a larger CPP_Rg_ and a smaller tilt angle corresponded to a higher EE. The final expanded linear predictor η was defined as a weighted combination of standardized CPP_Rg_, tilt angle, and N_OH_,

$$\eta =w_{1}\;\left({\mathrm{CPP}}_{R_{g}}\right)+w_{2}\left(\mathrm{Tilt}\;\mathrm{Angle}\right)+w_{3}\left(N_{\mathrm{OH}}\right)$$

where the regression coefficients *w*_1_,*w*_2_, *w*_3_ were determined by least‐squares minimization.

## Author Contributions

**Sanghyuk Jeon**: writing – original draft, writing – review and editing, visualization, validation, conceptualization, methodology, software, data curation, investigation, formal analysis, project administration. **Seohyeon Bae**: conceptualization, methodology, data curation, investigation, validation, formal analysis, supervision, writing – review and editing, resources, project administration. **Jungyong Ji**: investigation, conceptualization, methodology, validation, visualization, software, formal analysis, data curation, Writing – review and editing, Writing – original draft. **Jisun Lee**: writing – review and editing, validation, methodology, supervision, data curation, funding acquisition, conceptualization. **Hosam Choi**: methodology, investigation, resources. **Min‐Ho Kang**: methodology, validation, visualization, writing – review and editing, investigation, formal analysis, data curation. **Sang‐In Park**: methodology, validation, visualization, writing – review and editing, investigation, formal analysis, data curation. **Hyemin Kim**: writing – review and editing, validation, methodology, investigation. **Nakyung Lee**: methodology, investigation, writing – original draft. **Hajin Lee**: methodology, validation, investigation, software. **Seonghoon Kim**: methodology, validation, investigation, software. **Jungmin Kim**: methodology, investigation, data curation, conceptualization. **Subin Yoon**: methodology, investigation. **Seonghyun Lee**: methodology, investigation. **Seongje Cho**: methodology, investigation. **Dahyeon Ha**: methodology, investigation. **Ayoung Oh**: methodology, investigation. **Sohee Jo**: methodology, investigation. **Huijeong Choi**: methodology, investigation. **Yeeun Lee**: methodology, investigation. **Sowon Lee**: methodology, investigation. **Hyo‐Jung Park**: methodology, investigation. **Gitak Nam**: methodology, investigation. **Jisu Shin**: methodology, investigation. **Yujin Kang**: methodology, investigation. **Wonpil Im**: project administration, supervision, resources, conceptualization, writing – review and editing, software. **Kiyoun Lee**: conceptualization, writing – review and editing, project administration, resources, supervision. **Jae‐Hwan Nam**: conceptualization, funding acquisition, writing – review and editing, project administration, resources, supervision.

## Conflicts of Interest

The authors declare no conflicts of interest.

## Supporting information




**Supporting File**: advs76671‐sup‐0001‐SuppMat.docx.

## Data Availability

Source data are available for the study. Data supporting the findings of this study are available from the corresponding authors upon request.
