# Supplementary material for: Molecular Dynamics‐Guided Sterol Engineering of mRNA‐Lipid Nanoparticles Reprograms Biodistribution and Enhances Spleen‐Selective Immunity
Source: Adv Sci (Weinh). 2026 Jul 21:e76671. Online ahead of print. doi: 10.1002/advs.76671 (PMC13387038; doi:10.1002/advs.76671)
Supplement: Supplementary file 1 — Supporting File: advs76671‐sup‐0001‐SuppMat.docx. [file ADVS-9999-e76671-s001.docx]

Supporting Information

**Molecular Dynamics-Guided Sterol Engineering of mRNA-Lipid Nanoparticles Reprograms Biodistribution and Enhances Spleen-Selective Immunity**

Sanghyuk Jeon, Seohyeon Bae, Jungyong Ji, Jisun Lee, Hosam Choi, Min-Ho Kang, Sang-In Park, Hyemin Kim, Nakyung Lee, Hajin Lee, Seonghoon Kim, Jungmin Kim, Subin Yoon, Seonghyun Lee, Seongje Cho, Dahyeon Ha, Ayoung Oh, Sohee Jo, Huijeong Choi, Yeeun Lee, Sowon Lee, Hyo-Jung Park, Gitak Nam, Jisu Shin, Yujin Kang, Wonpil Im^*^, Kiyoun Lee^*^, and Jae-Hwan Nam^*^

**Supplementary Methods**

**1. Synthesis of Bile acid-derived sterols**

**1.1 General Methods**

All reactions were conducted in oven-dried glassware under nitrogen. Unless otherwise stated, all reagents were purchased from Sigma-Aldrich, TCI, or Fisher and were used without further purification. All solvents were ACS grade or better and used without further purification. Analytical thin layer chromatography (TLC) was performed with glass backed silica gel (60 Å) plates with fluorescent indication (Whatman). Visualization was accomplished by UV irradiation at 254 nm and/or by staining with ceric ammonium molybdate (CAM) solution or *p*-anisaldehyde solution. Flash column chromatography was performed by using silica gel (particle size 70-230 mesh ASTM). All ^1^H NMR and ^13^C NMR spectra were recorded at 298 K on a Bruker Avance III HD 500 (500 MHz) spectrometer in CDCl_3_ by using the signal of residual CHCl_3_, as an internal standard. All NMR *δ* values are given in ppm, and all *J* values are in Hz.

**1.2 Chemical Synthesis Procedure**

**1.2.1. Synthesis of Butyl (R)-4-((3R,5R,8R,9S,10S,13R,14S,17R)-3-hydroxy-10,13-dimethylhexadecahydro-1H-cyclopenta[a]phenanthren-17-yl)pentanoate (Butyl lithocholate; LCA-4)**

A solution of **Lithocholic acid** (200 mg, 1.0 equiv) in DMF (1.0 mL, 0.5 M) was treated with imidazole (216.5 mg, 6.0 equiv), TBSCl (239.6 mg, 3.0 equiv), and DMAP (6.5 mg, 10 mol%) and stirred at room temperature for 12 h, after which H₂O (5 mL) was added and the mixture was extracted with EtOAc (5 mL × 2); the combined organic layers were concentrated in vacuo to afford a crude residue that was dissolved in MeOH (1 mL) and H₂O (1 mL), treated with K₂CO₃ (88.1 mg, 1.2 equiv), and stirred vigorously at room temperature for 1 h. After evaporation of the solvent in vacuo, the residue was dissolved in CH₂Cl₂ (5 mL), cooled to 4 °C, acidified with 6 M HCl aqueous solution, and extracted with CH₂Cl₂ (10 mL × 2); the combined organic layers were dried over anhydrous Na₂SO₄, filtered, and concentrated in vacuo to give a colorless oil used directly in the next step. To this residue dissolved in CH₂Cl₂ (1.3 mL, 0.4 M) were added EDC·HCl (152.4 mg, 1.5 equiv) and DMAP (12.9 mg, 20 mol%), and the mixture was cooled to 4 °C and stirred under nitrogen for 30 min before 1-butanol (0.06 mL, 1.3 equiv) was added dropwise and the reaction was stirred at room temperature for 7 h. The reaction was quenched with saturated aqueous NaHCO₃ (5 mL), and the mixture was extracted with CH₂Cl₂ (5 mL × 3); the combined organic layers were dried over anhydrous Na₂SO₄, filtered, and concentrated in vacuo to give a colorless oil, which was dissolved in THF (5.3 mL, 0.1 M), cooled to 4 °C, and treated with TBAF (1.06 mL, 1.0 M in THF, 2.0 equiv); after stirring for 2 h at room temperature, the reaction was quenched with H₂O (5 mL), extracted with CH₂Cl₂ (5 mL × 3), and the combined organic layers were dried over anhydrous Na₂SO₄, filtered, and concentrated in vacuo, and the residue was purified by column chromatography (SiO₂, EtOAc/hexane = 1:10) to afford **LCA-4** (80 mg, 35%) as a colorless oil.; 1H NMR (CDCl3, 500 MHz): δ 4.04 (m, 2H), 3.53-3.64 (m, 1H), 2.31 (ddd, J = 15.27, 10.05, 5.19 Hz, 1H), 2.12-2.23 (m, 1H), 1.92 (ddd, J = 16.56, 9.69, 6.70 Hz, 1H), 1.68-1.90 (m, 6H), 1.44-1.67 (m, 5H), 1.83-0.43(m, 27H), 0.61(s, 3H); 13C NMR (CDCl3, 125 MHz): δ 174.5, 77.3, 77.1, 76.8, 71.8, 64.1, 56.5, 56.0, 42.7, 42.1, 40.4, 40.2, 36.4, 35.8, 35.4, 35.3, 34.6, 31.3, 31.0, 30.69, 30.5, 28.2, 27.2, 26.4, 24.2, 23.4, 20.8, 19.2, 18.2, 13.7, 12.0; HRMS (FAB, m/z): [M+H]+ calcd for C28H48O3, 433.3682; found: 433.3680.

**1.2.2. Synthesis of 2-hexyldecyl (4R)-4-((3R,5R,8R,9S,10S,13R,14S,17R)-3-hydroxy-10,13-dimethylhexadecahydro-1H-cyclopenta[a]phenanthren-17-yl)pentanoate (2-Hexyldecyl lithocholate; LCA-16)**

A solution of **Lithocholic acid** (200 mg, 1.0 equiv) in DMF (1.0 mL, 0.5 M) was treated with imidazole (216.5 mg, 6.0 equiv), TBSCl (239.6 mg, 3.0 equiv), and DMAP (6.5 mg, 10 mol%) and stirred at room temperature for 12 h, after which H₂O (5 mL) was added and the mixture was extracted with EtOAc (5 mL × 2); the combined organic layers were concentrated in vacuo to afford a crude residue that was dissolved in MeOH (1 mL) and H₂O (1 mL), treated with K₂CO₃ (88.1 mg, 1.2 equiv), and stirred vigorously at room temperature for 1 h. After evaporation of the solvent in vacuo, the residue was dissolved in CH₂Cl₂ (5 mL), cooled to 4 °C, acidified with 6 M HCl aqueous solution, and extracted with CH₂Cl₂ (10 mL × 2); the combined organic layers were dried over anhydrous Na₂SO₄, filtered, and concentrated in vacuo to give a colorless oil used directly in the next step. To this residue dissolved in CH₂Cl₂ (1.3 mL, 0.4 M) were added EDC·HCl (152.4 mg, 1.5 equiv) and DMAP (12.9 mg, 20 mol%), and the mixture was cooled to 4 °C and stirred under nitrogen for 30 min before 2-Hexyl-1-decanol (0.19 mL, 1.3 equiv) was added dropwise and the reaction was stirred at room temperature for 7 h. The reaction was quenched with saturated aqueous NaHCO₃ (5 mL), and the mixture was extracted with CH₂Cl₂ (5 mL × 3); the combined organic layers were dried over anhydrous Na₂SO₄, filtered, and concentrated in vacuo to give a colorless oil, which was dissolved in THF (5.3 mL, 0.1 M), cooled to 4 °C, and treated with TBAF (1.06 mL, 1.0 M in THF, 2.0 equiv); after stirring for 2 h at room temperature, the reaction was quenched with H₂O (5 mL), extracted with CH₂Cl₂ (5 mL × 3), and the combined organic layers were dried over anhydrous Na₂SO₄, filtered, and concentrated in vacuo, and the residue was purified by column chromatography (SiO₂, EtOAc/hexane = 1:20 to 1:10) to afford **LCA-16** (175.5 mg, 55%) as a colorless oil.; 1H NMR (CDCl3, 500 MHz): δ 3.94 (d, J = 5.7 Hz, 2H), 3.61 (ddd, J = 15.61, 10.86, 4.56 Hz, 1H), 2.33 (ddd, J = 15.56, 9.96, 5.17 Hz, 1H), 2.15-2.25 (m, 1H), 1.94 (dd, J = 9.45, 2.68 Hz, 1H), 1.69-1.89 (m, 7H), 1.44-1.67 (m, 5H), 0.76-1.44 (m, 62H), 0.62 (s, 3H); 13C NMR (CDCl3, 125 MHz): δ 174.6, 77.3, 77.1, 76.8, 71.8, 67.1, 56.5, 56.0, 42.7, 42.1, 40.4, 40.2, 37.3, 36.4, 35.8, 35.4, 34.6, 31.9, 31.8, 31.4, 31.3, 30.0, 29.7, 29.6, 29.6, 29.3, 26.71, 26.68, 26.4, 23.4, 22.70, 22.67, 18.2, 14.14, 14.12, 12.1; HRMS (FAB, m/z): [M+H]+ calcd for C40H72O3, 601.5560; found: 601.5554.

**1.2.3. Synthesis of 2-octyldodecyl (4R)-4-((3R,5R,8R,9S,10S,13R,14S,17R)-3-hydroxy-10,13-dimethylhexadecahydro-1H-cyclopenta[a]phenanthren-17-yl)pentanoate (2-Octyldodecyl lithocholate; LCA-20)**

A solution of **Lithocholic acid** (200 mg, 1.0 equiv) in DMF (1.0 mL, 0.5 M) was treated with imidazole (216.5 mg, 6.0 equiv), TBSCl (239.6 mg, 3.0 equiv), and DMAP (6.5 mg, 10 mol%) and stirred at room temperature for 12 h, after which H₂O (5 mL) was added and the mixture was extracted with EtOAc (5 mL × 2); the combined organic layers were concentrated in vacuo to afford a crude residue that was dissolved in MeOH (1 mL) and H₂O (1 mL), treated with K₂CO₃ (88.1 mg, 1.2 equiv), and stirred vigorously at room temperature for 1 h. After evaporation of the solvent in vacuo, the residue was dissolved in CH₂Cl₂ (5 mL), cooled to 4 °C, acidified with 6 M HCl aqueous solution, and extracted with CH₂Cl₂ (10 mL × 2); the combined organic layers were dried over anhydrous Na₂SO₄, filtered, and concentrated in vacuo to give a colorless oil used directly in the next step. To this residue dissolved in CH₂Cl₂ (1.3 mL, 0.4 M) were added EDC·HCl (152.4 mg, 1.5 equiv) and DMAP (12.9 mg, 20 mol%), and the mixture was cooled to 4 °C and stirred under nitrogen for 30 min before 2-Octyl-1-dodecanol (0.24 mL, 1.3 equiv) was added dropwise and the reaction was stirred at room temperature for 7 h. The reaction was quenched with saturated aqueous NaHCO₃ (5 mL), and the mixture was extracted with CH₂Cl₂ (5 mL × 3); the combined organic layers were dried over anhydrous Na₂SO₄, filtered, and concentrated in vacuo to give a colorless oil, which was dissolved in THF (5.3 mL, 0.1 M), cooled to 4 °C, and treated with TBAF (1.06 mL, 1.0 M in THF, 2.0 equiv); after stirring for 2 h at room temperature, the reaction was quenched with H₂O (5 mL), extracted with CH₂Cl₂ (5 mL × 3), and the combined organic layers were dried over anhydrous Na₂SO₄, filtered, and concentrated in vacuo, and the residue was purified by column chromatography (SiO₂, EtOAc/hexane = 1:20) to afford **LCA-20** (209.0 mg, 60%) as a colorless oil.; 1H NMR (CDCl3, 500 MHz): δ 3.94 (d, J = 5.7 Hz, 2H), 3.55-3.66 (m, 1H), 2.33 (ddd, J = 15.15, 9.93, 5.17 Hz, 1H), 2.14-2.25 (m, 1H), 1.94 (dd, J = 9.44, 2.68 Hz, 1H), 1.43-1.89 (m, 11H), 0.76-1.43 (m, 63H), 0.62 (s, 3H); 13C NMR (CDCl3, 125 MHz): δ 174.6, 77.30, 77.25, 77.1, 76.8, 71.8, 67.1, 56.5, 56.0, 42.7, 42.1, 40.4, 40.2, 37.3, 36.4, 35.8, 35.4, 34.6, 31.93, 31.92, 31.4, 31.3, 31.1, 30.0, 29.69, 29.67, 29.63, 29.58, 29.38, 29.35, 28.2, 27.2, 26.7, 26.4, 24.2, 23.4, 22.7, 20.8, 18.2, 14.1, 12.1; HRMS (FAB, m/z): [M+H]+ calcd for C44H80O3, 657.6186; found: 657.6183.

**1.2.4. Synthesis of Butyl (R)-4-((3R,5R,8R,9S,10S,12S,13R,14S,17R)-3,12-dihydroxy-10,13-dimethylhexadecahydro-1H-cyclopenta[a]phenanthren-17-yl)pentanoate (Butyl deoxycholate; DCA-4)**

A solution of **Deoxycholic acid** (200 mg, 1.0 equiv) in DMF (1.0 mL, 0.5 M) was treated with imidazole (486.0 mg, 14.0 equiv), TBSCl (538.0 mg, 7.0 equiv), and DMAP (6.2 mg, 10 mol%) and stirred at room temperature for 12 h, after which H₂O (5 mL) was added and the mixture was extracted with EtOAc (5 mL × 2); the combined organic layers were concentrated in vacuo to afford a crude residue that was dissolved in MeOH (1 mL) and H₂O (1 mL), treated with K₂CO₃ (84.5 mg, 1.2 equiv), and stirred vigorously at room temperature for 1 h. After evaporation of the solvent in vacuo, the residue was dissolved in CH₂Cl₂ (5 mL), cooled to 4 °C, acidified with 6 M HCl aqueous solution, and extracted with CH₂Cl₂ (10 mL × 2); the combined organic layers were dried over anhydrous Na₂SO₄, filtered, and concentrated in vacuo to give a colorless oil used directly in the next step. To this residue dissolved in CH₂Cl₂ (1.3 mL, 0.4 M) were added EDC·HCl (146.6 mg, 1.5 equiv) and DMAP (12.5 mg, 20 mol%), and the mixture was cooled to 4 °C and stirred under nitrogen for 30 min before 1-Butanol (0.06 mL, 1.3 equiv) was added dropwise and the reaction was stirred at room temperature for 7 h. The reaction was quenched with saturated aqueous NaHCO₃ (5 mL), and the mixture was extracted with CH₂Cl₂ (5 mL × 3); the combined organic layers were dried over anhydrous Na₂SO₄, filtered, and concentrated in vacuo to give a colorless oil, which was dissolved in THF (5.1 mL, 0.1 M), cooled to 4 °C, and treated with TBAF (1.53 mL, 1.0 M in THF, 3.0 equiv); after stirring for 2 h at room temperature, the reaction was quenched with H₂O (5 mL), extracted with CH₂Cl₂ (5 mL × 3), and the combined organic layers were dried over anhydrous Na₂SO₄, filtered, and concentrated in vacuo, and the residue was purified by column chromatography (SiO₂, EtOAc/hexane = 1:5) to afford **DCA-4** (148.0 mg, 65%) as a colorless oil.; 1H NMR (CDCl3, 500 MHz): δ 4.04 (t, J = 6.66 Hz, 2H), 3.96 (s, 1H), 3.53-3.64 (m, 1H), 2.34 (ddd, J = 14.86, 9.86, 4.94 Hz, 1H), 2.14-2.24 (m, 1H), 1.94-2.14 (m, 2H), 1.44-1.91 (m, 15H), 0.73-1.45 (m, 25H), 0.65 (s, 3H); 13C NMR (CDCl3, 125 MHz): δ 174.4, 77.3, 77.1, 76.8, 73.1, 71.7, 64.1, 48.2, 47.3, 46.5, 42.1, 36.4, 36.0, 35.24, 35.16, 34.1, 33.6, 31.4, 31.0, 30.7, 30.4, 29.7, 28.6, 27.5, 27.1, 26.1, 23.7, 23.1, 19.2, 17.3, 13.7, 12.7; HRMS (FAB, m/z): [M-H2O+H]+ calcd for C28H48O4, 413.3420; found: 413.3420.

**1.2.5. Synthesis of 2-hexyldecyl (4R)-4-((3R,5R,8R,9S,10S,12S,13R,14S,17R)-3,12-dihydroxy-10,13-dimethylhexadecahydro-1H-cyclopenta[a]phenanthren-17-yl)pentanoate (2-Hexyldecyl deoxycholate; DCA-16)**

A solution of **Deoxycholic acid** (200 mg, 1.0 equiv) in DMF (1.0 mL, 0.5 M) was treated with imidazole (486.0 mg, 14.0 equiv), TBSCl (538.0 mg, 7.0 equiv), and DMAP (6.2 mg, 10 mol%) and stirred at room temperature for 12 h, after which H₂O (5 mL) was added and the mixture was extracted with EtOAc (5 mL × 2); the combined organic layers were concentrated in vacuo to afford a crude residue that was dissolved in MeOH (1 mL) and H₂O (1 mL), treated with K₂CO₃ (84.5 mg, 1.2 equiv), and stirred vigorously at room temperature for 1 h. After evaporation of the solvent in vacuo, the residue was dissolved in CH₂Cl₂ (5 mL), cooled to 4 °C, acidified with 6 M HCl aqueous solution, and extracted with CH₂Cl₂ (10 mL × 2); the combined organic layers were dried over anhydrous Na₂SO₄, filtered, and concentrated in vacuo to give a colorless oil used directly in the next step. To this residue dissolved in CH₂Cl₂ (1.3 mL, 0.4 M) were added EDC·HCl (146.6 mg, 1.5 equiv) and DMAP (12.5 mg, 20 mol%), and the mixture was cooled to 4 °C and stirred under nitrogen for 30 min before 2-Hexyl-1-decanol (0.19 mL, 1.3 equiv) was added dropwise and the reaction was stirred at room temperature for 7 h. The reaction was quenched with saturated aqueous NaHCO₃ (5 mL), and the mixture was extracted with CH₂Cl₂ (5 mL × 3); the combined organic layers were dried over anhydrous Na₂SO₄, filtered, and concentrated in vacuo to give a colorless oil, which was dissolved in THF (5.1 mL, 0.1 M), cooled to 4 °C, and treated with TBAF (1.53 mL, 1.0 M in THF, 3.0 equiv); after stirring for 2 h at room temperature, the reaction was quenched with H₂O (5 mL), extracted with CH₂Cl₂ (5 mL × 3), and the combined organic layers were dried over anhydrous Na₂SO₄, filtered, and concentrated in vacuo, and the residue was purified by column chromatography (SiO₂, EtOAc/hexane = 1:3) to afford **DCA-16** (195.0 mg, 62%) as a colorless oil.; 1H NMR (CDCl3, 500 MHz): δ 3.95 (dd, J = 8.43, 4.29 Hz, 3H), 3.59-3.62 (m, 1H), 2.34 (ddd, J = 14.89, 9.80, 4.90 Hz, 1H), 2.15-2.25 (m, 1H), 1.45-1.90 (m, 16H), 1.19-1.44 (m, 35H), 0.78-1.17 (m, 17H), 0.65 (s, 3H); 13C NMR (CDCl3, 125 MHz): δ 174.5, 77.3, 77.1, 76.8, 73.1, 71.7, 67.1, 48.2, 47.3, 46.5, 42.1, 37.3, 36.4, 36.00, 35.2, 35.1, 34.1, 33.6, 31.9, 31.8, 31.4, 31.3, 31.0, 30.5, 30.0, 29.6, 29.6, 29.3, 28.7, 27.5, 27.1, 26.71, 26.67, 26.1, 23.7, 23.1, 22.69, 22.66, 17.2, 14.13, 14.11, 12.7; HRMS (FAB, m/z): [M-H2O+H]+ calcd for C40H72O4, 599.5403; found: 599.5405.

**1.2.6. Synthesis of 2-octyldodecyl (4R)-4-((3R,5R,8R,9S,10S,12S,13R,14S,17R)-3,12-dihydroxy-10,13-dimethylhexadecahydro-1H-cyclopenta[a]phenanthren-17-yl)pentanoate (2-Octyldodecyl deoxycholate; DCA-20)**

A solution of **Deoxycholic acid** (200 mg, 1.0 equiv) in DMF (1.0 mL, 0.5 M) was treated with imidazole (486.0 mg, 14.0 equiv), TBSCl (538.0 mg, 7.0 equiv), and DMAP (6.2 mg, 10 mol%) and stirred at room temperature for 12 h, after which H₂O (5 mL) was added and the mixture was extracted with EtOAc (5 mL × 2); the combined organic layers were concentrated in vacuo to afford a crude residue that was dissolved in MeOH (1 mL) and H₂O (1 mL), treated with K₂CO₃ (84.5 mg, 1.2 equiv), and stirred vigorously at room temperature for 1 h. After evaporation of the solvent in vacuo, the residue was dissolved in CH₂Cl₂ (5 mL), cooled to 4 °C, acidified with 6 M HCl aqueous solution, and extracted with CH₂Cl₂ (10 mL × 2); the combined organic layers were dried over anhydrous Na₂SO₄, filtered, and concentrated in vacuo to give a colorless oil used directly in the next step. To this residue dissolved in CH₂Cl₂ (1.3 mL, 0.4 M) were added EDC·HCl (146.6 mg, 1.5 equiv) and DMAP (12.5 mg, 20 mol%), and the mixture was cooled to 4 °C and stirred under nitrogen for 30 min before 2-Octyl-1-dodecanol (0.23 mL, 1.3 equiv) was added dropwise and the reaction was stirred at room temperature for 7 h. The reaction was quenched with saturated aqueous NaHCO₃ (5 mL), and the mixture was extracted with CH₂Cl₂ (5 mL × 3); the combined organic layers were dried over anhydrous Na₂SO₄, filtered, and concentrated in vacuo to give a colorless oil, which was dissolved in THF (5.1 mL, 0.1 M), cooled to 4 °C, and treated with TBAF (1.53 mL, 1.0 M in THF, 3.0 equiv); after stirring for 2 h at room temperature, the reaction was quenched with H₂O (5 mL), extracted with CH₂Cl₂ (5 mL × 3), and the combined organic layers were dried over anhydrous Na₂SO₄, filtered, and concentrated in vacuo, and the residue was purified by column chromatography (SiO₂, EtOAc/hexane = 1:3) to afford **DCA-20** (188.0 mg, 55%) as a colorless oil.; 1H NMR (CDCl3, 500 MHz): δ 3.87-3.98 (m, 3H), 3.55 (ddd, J = 15.20, 10.48, 4.33 Hz, 1H), 3.03 (br s, 1H), 2.31 (ddd, J = 14.60, 9.61, 4.75 Hz, 1H), 2.18 (dt, J = 15.41, 7.87 Hz, 1H), 0.73-1.87 (m, 76H), 0.63 (s, 3H); 13C NMR (CDCl3, 125 MHz): δ 174.4, 77.4, 77.11, 76.8, 73.0, 71.6, 67.1, 48.2, 47.2, 46.5, 42.1, 37.3, 36.4, 36.0, 35.3, 35.2, 34.1, 33.5, 31.92, 31.90, 31.5, 31.2, 31.0, 30.4, 30.0, 29.7, 29.7, 29.63, 29.58, 29.4, 29.3, 28.7, 27.5, 27.2, 26.7, 26.1, 23.7, 23.1, 22.7, 17.2, 14.1, 12.7; HRMS (FAB, m/z): [M-H2O+H]+ calcd for C44H80O4, 655.6029; found: 655.6033.

**1.2.7. Synthesis of Butyl (R)-4-((3R,5S,7R,8R,9S,10S,12S,13R,14S,17R)-3,7,12-trihydroxy-10,13-dimethylhexadecahydro-1H-cyclopenta[a]phenanthren-17-yl)pentanoate (Butyl cholate; CA-4)**

A solution of **Cholic acid** (200 mg, 1.0 equiv) in DMF (0.9 mL, 0.5 M) was treated with imidazole (665.8 mg, 20.0 equiv), TBSCl (737.8 mg, 10.0 equiv), and DMAP (5.9 mg, 10 mol%) and stirred at room temperature for 12 h, after which H₂O (5 mL) was added and the mixture was extracted with EtOAc (5 mL × 2); the combined organic layers were concentrated in vacuo to afford a crude residue that was dissolved in MeOH (1 mL) and H₂O (1 mL), treated with K₂CO₃ (81.1 mg, 1.2 equiv), and stirred vigorously at room temperature for 1 h. After evaporation of the solvent in vacuo, the residue was dissolved in CH₂Cl₂ (5 mL), cooled to 4 °C, acidified with 6 M HCl aqueous solution, and extracted with CH₂Cl₂ (10 mL × 2); the combined organic layers were dried over anhydrous Na₂SO₄, filtered, and concentrated in vacuo to give a colorless oil used directly in the next step. To this residue dissolved in CH₂Cl₂ (1.2 mL, 0.4 M) were added EDC·HCl (140.9 mg, 1.5 equiv) and DMAP (11.9 mg, 20 mol%), and the mixture was cooled to 4 °C and stirred under nitrogen for 30 min before 1-Butanol (0.06 mL, 1.3 equiv) was added dropwise and the reaction was stirred at room temperature for 7 h. The reaction was quenched with saturated aqueous NaHCO₃ (5 mL), and the mixture was extracted with CH₂Cl₂ (5 mL × 3); the combined organic layers were dried over anhydrous Na₂SO₄, filtered, and concentrated in vacuo to give a colorless oil, which was dissolved in THF (4.9 mL, 0.1 M), cooled to 4 °C, and treated with TBAF (2.45 mL, 1.0 M in THF, 5.0 equiv); after stirring for 2 h at room temperature, the reaction was quenched with H₂O (5 mL), extracted with CH₂Cl₂ (5 mL × 3), and the combined organic layers were dried over anhydrous Na₂SO₄, filtered, and concentrated in vacuo, and the residue was purified by column chromatography (SiO₂, EtOAc/hexane = 1:1) to afford **CA-4** (145.0 mg, 64%) as a colorless oil.; 1H NMR (CDCl3, 500 MHz): δ 4.05 (t, J = 6.66 Hz, 2H), 3.95 (s, 1H), 3.82 (d, J = 2.35 Hz, 1H), 2.74 (br s, 1H), 2.52 (br s, 1H), 2.35 (ddd, J = 14.73, 9.76, 4.78 Hz, 1H), 2.21 (qd, J = 12.43, 4.53 Hz, 3H), 1.20-1.98 (m, 23H), 1.01-1.14 (m, 1H), 0.81-1.01 (m, 10H), 0.66 (s, 3H); 13C NMR (CDCl3, 125 MHz): δ 174.5, 77.3, 77.0, 76.8, 73.1, 71.9, 68.5, 64.2, 47.1, 46.5, 41.6, 41.5, 39.5, 35.3, 34.8, 34.7, 31.4, 31.0, 30.7, 30.5, 28.2, 27.5, 26.4, 23.2, 22.47, 19.2, 17.3, 13.7, 12.5; HRMS (FAB, m/z): [M+H]+ calcd for C28H48O5, 465.3580; found: 465.3578.

**1.2.8. Synthesis of 2-hexyldecyl (4R)-4-((3R,5S,7R,8R,9S,10S,12S,13R,14S,17R)-3,7,12-trihydroxy-10,13-dimethylhexadecahydro-1H-cyclopenta[a]phenanthren-17-yl)pentanoate (2-Hexyldecyl cholate; CA-16)**

A solution of **Cholic acid** (200 mg, 1.0 equiv) in DMF (0.9 mL, 0.5 M) was treated with imidazole (665.8 mg, 20.0 equiv), TBSCl (737.8 mg, 10.0 equiv), and DMAP (5.9 mg, 10 mol%) and stirred at room temperature for 12 h, after which H₂O (5 mL) was added and the mixture was extracted with EtOAc (5 mL × 2); the combined organic layers were concentrated in vacuo to afford a crude residue that was dissolved in MeOH (1 mL) and H₂O (1 mL), treated with K₂CO₃ (81.1 mg, 1.2 equiv), and stirred vigorously at room temperature for 1 h. After evaporation of the solvent in vacuo, the residue was dissolved in CH₂Cl₂ (5 mL), cooled to 4 °C, acidified with 6 M HCl aqueous solution, and extracted with CH₂Cl₂ (10 mL × 2); the combined organic layers were dried over anhydrous Na₂SO₄, filtered, and concentrated in vacuo to give a colorless oil used directly in the next step. To this residue dissolved in CH₂Cl₂ (1.2 mL, 0.4 M) were added EDC·HCl (140.9 mg, 1.5 equiv) and DMAP (11.9 mg, 20 mol%), and the mixture was cooled to 4 °C and stirred under nitrogen for 30 min before 2-Hexyl-1-decanol (0.18 mL, 1.3 equiv) was added dropwise and the reaction was stirred at room temperature for 7 h. The reaction was quenched with saturated aqueous NaHCO₃ (5 mL), and the mixture was extracted with CH₂Cl₂ (5 mL × 3); the combined organic layers were dried over anhydrous Na₂SO₄, filtered, and concentrated in vacuo to give a colorless oil, which was dissolved in THF (4.9 mL, 0.1 M), cooled to 4 °C, and treated with TBAF (2.45 mL, 1.0 M in THF, 5.0 equiv); after stirring for 2 h at room temperature, the reaction was quenched with H₂O (5 mL), extracted with CH₂Cl₂ (5 mL × 3), and the combined organic layers were dried over anhydrous Na₂SO₄, filtered, and concentrated in vacuo, and the residue was purified by column chromatography (SiO₂, EtOAc/hexane = 1:2 to 1:1) to afford **CA-16** (213.0 mg, 69%) as a colorless oil.; 1H NMR (CDCl3, 500 MHz): δ 3.97 (d, J = 5.57 Hz, 3H), 3.84 (d, J = 1.73 Hz, 1H), 3.38-3.50 (m, 1H), 3.12 (br s, 1H), 2.85 (br s, 1H), 2.63 (br s, 1H), 2.37 (ddd, J = 14.43, 9.53, 4.63 Hz, 1H), 2.15-2.31 (m, 3H), 1.47-1.97 (m, 14H), 1.21-1.46 (m, 29H), 1.05-1.16 (m, 1H), 0.81-1.05 (m, 13H), 0.68 (s, 3H); 13C NMR (CDCl3, 125 MHz): δ 174.6, 77.3, 77.1, 76.8, 73.1, 71.9, 68.5, 67.1, 47.1, 46.4, 41.6, 41.5, 39.5, 37.3, 35.28, 35.25, 34.8, 34.7, 31.9, 31.8, 31.4, 31.3, 31.0, 30.4, 30.0, 29.6, 29.6, 29.3, 28.2, 27.5, 26.71, 26.67, 26.3, 23.2, 22.69, 22.66, 22.4, 17.3, 14.13, 14.12, 12.5; HRMS (FAB, m/z): [M-2H2O+H]+ calcd for C40H72O5, 597.5247; found: 597.5240.

**1.2.9. Synthesis of 2-octyldodecyl (4R)-4-((3R,5S,7R,8R,9S,10S,12S,13R,14S,17R)-3,7,12-trihydroxy-10,13-dimethylhexadecahydro-1H-cyclopenta[a]phenanthren-17-yl)pentanoate (2-Octyldodecyl cholate; CA-20)**

A solution of **Cholic acid** (200 mg, 1.0 equiv) in DMF (0.9 mL, 0.5 M) was treated with imidazole (665.8 mg, 20.0 equiv), TBSCl (737.8 mg, 10.0 equiv), and DMAP (5.9 mg, 10 mol%) and stirred at room temperature for 12 h, after which H₂O (5 mL) was added and the mixture was extracted with EtOAc (5 mL × 2); the combined organic layers were concentrated in vacuo to afford a crude residue that was dissolved in MeOH (1 mL) and H₂O (1 mL), treated with K₂CO₃ (81.1 mg, 1.2 equiv), and stirred vigorously at room temperature for 1 h. After evaporation of the solvent in vacuo, the residue was dissolved in CH₂Cl₂ (5 mL), cooled to 4 °C, acidified with 6 M HCl aqueous solution, and extracted with CH₂Cl₂ (10 mL × 2); the combined organic layers were dried over anhydrous Na₂SO₄, filtered, and concentrated in vacuo to give a colorless oil used directly in the next step. To this residue dissolved in CH₂Cl₂ (1.2 mL, 0.4 M) were added EDC·HCl (140.9 mg, 1.5 equiv) and DMAP (11.9 mg, 20 mol%), and the mixture was cooled to 4 °C and stirred under nitrogen for 30 min before 2-Octyl-1-dodecanol (0.22 mL, 1.3 equiv) was added dropwise and the reaction was stirred at room temperature for 7 h. The reaction was quenched with saturated aqueous NaHCO₃ (5 mL), and the mixture was extracted with CH₂Cl₂ (5 mL × 3); the combined organic layers were dried over anhydrous Na₂SO₄, filtered, and concentrated in vacuo to give a colorless oil, which was dissolved in THF (4.9 mL, 0.1 M), cooled to 4 °C, and treated with TBAF (2.45 mL, 1.0 M in THF, 5.0 equiv); after stirring for 2 h at room temperature, the reaction was quenched with H₂O (5 mL), extracted with CH₂Cl₂ (5 mL × 3), and the combined organic layers were dried over anhydrous Na₂SO₄, filtered, and concentrated in vacuo, and the residue was purified by column chromatography (SiO₂, EtOAc/hexane = 1:2 to 1:1) to afford **CA-20** (202.0 mg, 60%) as a colorless oil.; 1H NMR (CDCl3, 500 MHz): δ 3.95 (d, J = 5.78 Hz, 3H), 3.82 (t, J = 9.99 Hz, 1H), 3.44 (ddd, J = 15.14, 10.75, 4.10 Hz, 1H), 2.53-2.95 (m, 3H), 2.35 (ddd, J = 14.58, 9.53, 4.78 Hz, 2H), 2.14-2.28 (m, 3H), 1.16-1.96 (m, 48H), 1.09 (ddd, J = 24.15, 11.98, 6.05 Hz, 1H), 0.78-1.01 (m, 12H), 0.66 (s, 3H); 13C NMR (CDCl3, 125 MHz): δ 174.6, 77.3, 77.1, 76.8, 73.1, 71.9, 68.5, 67.1, 47.0, 46.4, 41.5, 39.5, 39.4, 37.3, 35.3, 34.80, 34.75, 31.92, 31.91, 31.4, 31.2, 31.0, 30.4, 30.0, 29.67, 29.65, 29.63, 29.58, 29.4, 29.3, 28.2, 27.5, 26.7, 26.3, 23.3, 22.7, 22.4, 17.3, 14.1, 12.5; HRMS (FAB, m/z): [M-2H2O+H]+ calcd for C44H80O5, 653.5873; found: 653.5872.

**Supplementary Tables**

**Table S1.** Cholesterol, bile acids, and bile acid-derived sterol employed in this study with their code names, common names, and chemical structures.

| **Code name** | **Common name** | **Chemical structure** | **Code name** | **Common name** | **Chemical structure** |
| --- | --- | --- | --- | --- | --- |
| Chol | Cholesterol | 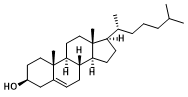 |  | | |
| LCA | Lithocholic acid | 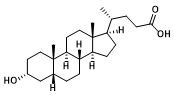 | LCA-16 | 2-Hexyldecyl lithocholate | 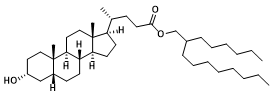 |
| DCA | Deoxycholic acid | 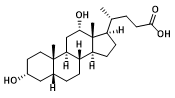 | DCA-16 | 2-Hexyldecyl deoxycholate | 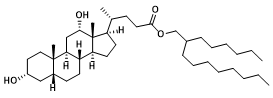 |
| CA | cholic acid | 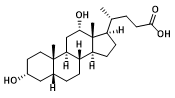 | CA-16 | 2-Hexyldecyl cholate | 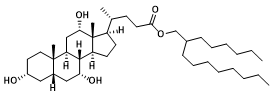 |
| LCA-4 | Butyl lithocholate | 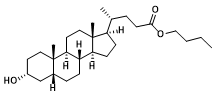 | LCA-20 | 2-Octyldodecyl lithocholate | 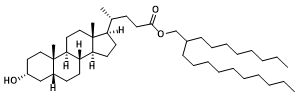 |
| DCA-4 | Butyl deoxycholate | 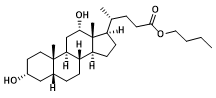 | DCA-20 | 2-Octyldodecyl deoxycholate | 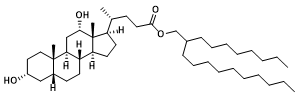 |
| CA-4 | Butyl cholate | 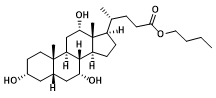 | CA-20 | 2-Octyldodecyl cholate | 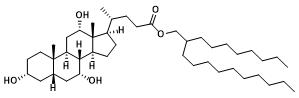 |

**Table S2.** Formulations and lipid compositions of the LNP samples.

| **LNP sample** | **N/P ratio** | **Ionizable lipid**  **(mol%)** | **Sterol lipid**  **(mol%)** | **Helper lipid**  **(mol%)** | **PEG-lipid**  **(mol%)** |
| --- | --- | --- | --- | --- | --- |
| Chol | 6 | SM-102  (50) | Cholesterol (Chol) (38.5) | DSPC  (10) | DMG-PEG 2K  (1.5) |
| LCA |  |  | Lithocholic acid (LCA) (38.5) |  |  |
| DCA |  |  | Deoxycholic acid (DCA) (38.5) |  |  |
| CA |  |  | cholic acid (CA) (38.5) |  |  |
| LCA-4 |  |  | Butyl lithocholate (LCA-4)  (38.5) |  |  |
| DCA-4 |  |  | Butyl deoxycholate (DCA-4) (38.5) |  |  |
| CA-4 |  |  | Butyl cholate (CA-4)  (38.5) |  |  |
| LCA-16 |  |  | 2-Hexyldecyl lithocholate (LCA-16) (38.5) |  |  |
| DCA-16 |  |  | 2-Hexyldecyl deoxycholate (DCA-16) (38.5) |  |  |
| CA-16 |  |  | 2-Hexyldecyl cholate (CA-16) (38.5) |  |  |
| LCA-20 |  |  | 2-Octyldodecyl lithocholate (LCA-20) (38.5) |  |  |
| DCA-20 |  |  | 2-Octyldodecyl deoxycholate (DCA-20) (38.5) |  |  |
| CA-20 |  |  | 2-Octyldodecyl cholate (CA-20) (38.5) |  |  |

**Table S3.** LNP bilayer structural properties.

| **Model** | **Tilt Angle (°)** | $\mathbf{S}_{\mathbf{CD}}$ | **CPP_Rg_** |
| --- | --- | --- | --- |
| Chol | 30.670 ± 1.170 | 0.267 ± 0.020 | 5.068 ± 0.015 |
| LCA | 123.505 ± 0.210 | 0.169 ± 0.004 | 1.532 ± 0.001 |
| DCA | 107.599 ± 0.509 | 0.140 ± 0.012 | 1.484 ± 0.002 |
| CA | 103.276 ± 0.580 | 0.127 ± 0.022 | 1.501 ± 0.001 |
| LCA-4 | 89.123 ± 3.180 | 0.181 ± 0.015 | 0.948 ± 0.002 |
| DCA-4 | 80.608 ± 0.346 | 0.161 ± 0.007 | 0.935 ± 0.004 |
| CA-4 | 67.729 ± 4.128 | 0.166 ± 0.015 | 0.932 ± 0.001 |
| LCA-16 | 85.664 ± 2.890 | 0.180 ± 0.011 | 9.249 ± 0.029 |
| DCA-16 | 71.401 ± 2.655 | 0.173 ± 0.010 | 9.314 ± 0.014 |
| CA-16 | 53.551 ± 4.190 | 0.191 ± 0.006 | 9.417 ± 0.021 |
| LCA-20 | 83.885 ± 6.130 | 0.178 ± 0.021 | 11.002 ± 0.053 |
| DCA-20 | 68.603 ± 3.096 | 0.167 ± 0.014 | 10.984 ± 0.081 |
| CA-20 | 51.161 ± 1.313 | 0.186 ± 0.004 | 11.194 ± 0.082 |
| Chol | 35.101 ± 1.890 | 0.284 ± 0.019 | 5.039 ± 0.064 |
| LCA | 126.026 ± 0.637 | 0.160 ± 0.005 | 1.539 ± 0.002 |
| DCA | 109.268 ± 1.188 | 0.104 ± 0.007 | 1.488 ± 0.002 |
| CA | 106.432 ± 0.822 | 0.097 ± 0.013 | 1.502 ± 0.001 |
| LCA-4 | 92.927 ± 3.768 | 0.191 ± 0.012 | 0.949 ± 0.004 |
| DCA-4 | 87.363 ± 3.294 | 0.170 ± 0.010 | 0.941 ± 0.004 |
| CA-4 | 76.238 ± 3.518 | 0.154 ± 0.012 | 0.933 ± 0.006 |
| LCA-16 | 87.935 ± 3.302 | 0.179 ± 0.011 | 9.162 ± 0.073 |
| DCA-16 | 79.205 ± 5.538 | 0.174 ± 0.008 | 9.288 ± 0.082 |
| CA-16 | 68.514 ± 3.478 | 0.136 ± 0.030 | 9.412 ± 0.062 |
| LCA-20 | 85.527 ± 3.411 | 0.178 ± 0.013 | 11.059 ± 0.065 |
| DCA-20 | 77.846 ± 2.319 | 0.176 ± 0.014 | 11.024 ± 0.079 |
| CA-20 | 64.676 ± 2.430 | 0.154 ± 0.004 | 11.096 ± 0.061 |
| *For each model, values are reported as the mean and standard deviation across three replicates.  **White rows represent the protonated condition, and gray rows represent the neutral condition. | | | |

**Table S4.** Simulation system information.

| **Model** | **IL**  **name** | **HL**  **name** | **Sterol**  **name** | **# molecules per leaflet** | | | | **Water**  **thickness (Å)** | **XYZ (Å^3^)** | **# Na** | **# Cl** | **# waters** | **#**  **atoms** | **Time (ns)** |
| --- | --- | --- | --- | --- | --- | --- | --- | --- | --- | --- | --- | --- | --- | --- |
|  |  |  |  | **IL (P)** | **IL (N)** | **HL** | **Sterol** |  |  |  |  |  |  |  |
| Chol | SM-102 | DSPC | Cholesterol | 25 | 25 | 10 | 38 | 20 | 87x87x81 | 24 | 24 | 8854 | 48774 | 1,500 |
| LCA |  |  | Litocholic  acid |  |  |  |  |  | 86x86x81 | 100 | 24 | 8266 | 46478 |  |
| DCA |  |  | Deoxycholic  acid |  |  |  |  |  | 86x86x82 | 100 | 24 | 8688 | 47820 |  |
| CA |  |  | Cholic  acid |  |  |  |  |  | 86x86x83 | 102 | 26 | 8939 | 48653 |  |
| LCA-4 |  |  | Butyl  litocholate |  |  |  |  |  | 86x86x84 | 26 | 26 | 9576 | 51324 |  |
| DCA-4 |  |  | Butyl  deoxycholate |  |  |  |  |  | 86x86x81 | 24 | 24 | 8747 | 48909 |  |
| CA-4 |  |  | Butyl  cholate |  |  |  |  |  | 86x86x85 | 26 | 26 | 9600 | 51548 |  |
| LCA-16 |  |  | 2-Hexyldecyl  litocholate |  |  |  |  |  | 87x87x81 | 24 | 24 | 8786 | 51686 |  |
| DCA-16 |  |  | 2-Hexyldecyl  deoxycholate |  |  |  |  |  | 88x88x80 | 24 | 24 | 8912 | 52140 |  |
| CA-16 |  |  | 2-Hexyldecyl  cholate |  |  |  |  |  | 88x88x85 | 28 | 28 | 9951 | 55341 |  |
| LCA-20 |  |  | 2-Octyldodecyl  litocholate |  |  |  |  |  | 88x88x81 | 24 | 24 | 9057 | 53411 |  |
| DCA-20 |  |  | 2-Octyldodecyl  deoxycholate |  |  |  |  |  | 86x86x83 | 26 | 26 | 9253 | 54079 |  |
| CA-20 |  |  | 2-Octyldodecyl  cholate |  |  |  |  |  | 87x87x85 | 28 | 28 | 9892 | 56076 |  |
| Chol | SM-102 | DSPC | Cholesterol | 0 | 50 | 10 | 38 | 20 | 86x86x91 | 30 | 80 | 10590 | 54094 | 1,500 |
| LCA |  |  | Litocholic  acid |  |  |  |  |  | 87x87x85 | 52 | 26 | 9598 | 50478 |  |
| DCA |  |  | Deoxycholic  acid |  |  |  |  |  | 86x86x86 | 52 | 26 | 9686 | 50818 |  |
| CA |  |  | Cholic  acid |  |  |  |  |  | 87x87x78 | 48 | 22 | 8312 | 46764 |  |
| LCA-4 |  |  | Butyl  litocholate |  |  |  |  |  | 88x88x91 | 30 | 80 | 11115 | 56049 |  |
| DCA-4 |  |  | Butyl  deoxycholate |  |  |  |  |  | 87x87x91 | 30 | 80 | 10723 | 54949 |  |
| CA-4 |  |  | Butyl  cholate |  |  |  |  |  | 86x86x92 | 30 | 80 | 10656 | 54824 |  |
| LCA-16 |  |  | 2-Hexyldecyl  litocholate |  |  |  |  |  | 86x86x91 | 30 | 80 | 10538 | 57054 |  |
| DCA-16 |  |  | 2-Hexyldecyl  deoxycholate |  |  |  |  |  | 87x87x91 | 30 | 80 | 10837 | 58027 |  |
| CA-16 |  |  | 2-Hexyldecyl  cholate |  |  |  |  |  | 86x86x90 | 28 | 78 | 10176 | 56116 |  |
| LCA-20 |  |  | 2-Octyldodecyl  litocholate |  |  |  |  |  | 85x85x91 | 28 | 78 | 10183 | 56897 |  |
| DCA-20 |  |  | 2-Octyldodecyl  deoxycholate |  |  |  |  |  | 86x86x91 | 30 | 80 | 10598 | 58222 |  |
| CA-20 |  |  | 2-Octyldodecyl  Cholate |  |  |  |  |  | 85x85x91 | 28 | 78 | 10250 | 57250 |  |
| *White rows represent the protonated condition, and gray rows represent the neutral condition. | | | | | | | | | | | | | | |

**Supplementary Figures**

**Figure S1. Flow cytometric analysis of spleen-associated mRNA expression following I.V. administration of Chol LNP and bile acid-derived sterol LNPs**

(A) Experimental scheme for flow cytometric analysis. Female BALB/c mice (6–7 weeks old, n = 4 biologically independent animals per group) were intravenously administered DPBS, Chol LNP, or bile acid-derived sterol LNPs encapsulating eGFP-encoding mRNA at a dose of 0.5 mg kg⁻¹. Spleens were harvested 4 h post-injection for splenocyte preparation and flow cytometric analysis. (B) Quantification of eGFP-positive cells among total splenocytes following administration of DPBS, Chol LNP, and bile acid-derived sterol LNPs. (C) Scatter plot comparing spleen FLuc expression measured by IVIS imaging and eGFP-positive cells among total splenocytes (parent %) measured by flow cytometry. The solid line represents a simple linear regression fit, and the dashed lines indicate the 95% confidence bands (R² = 0.69). Each point represents an LNP formulation. Point colors denote tail length series (native (unmodified), C4, C16, and C20), and point shapes denote sterol scaffolds (Chol, LCA, DCA, and CA), as indicated in the legend. (D–G) Quantification of eGFP-positive cells (parent %) in major splenic immune cell populations, including macrophages (CD45⁺CD11b⁺F4/80⁺) (D), dendritic cells (CD45⁺CD11b⁻CD11c⁺) (E), B cells (CD45⁺CD19⁺) (F), and T cells (CD45⁺CD3⁺) (G). Data are presented as individual data points with the mean ± SD. Statistical analysis was performed using one-way ANOVA with Dunnett’s multiple comparisons test (*p < 0.05, **p < 0.01, ***p < 0.001, ****p < 0.0001; ns, not significant), with all comparisons made relative to the Chol LNP group.

**Figure S2. In vivo local expression and organ-level biodistribution of SM-102-based LNPs following I.M. administration**

(A) Representative IVIS images acquired 6 h after intramuscular (I.M.) injection of FLuc mRNA-encoding LNPs (0.5 mg kg⁻¹) in female BALB/c mice (6-7 weeks old; n = 3 biologically independent animals) treated with DPBS, Chol LNPs, or bile acid-derived sterol LNPs. (B–D) Total flux (photons/s) in liver (B), spleen (C), and muscle (D) following I.M. administration. (E) Fractional distribution of expression (%) across the liver, spleen, heart, lung, kidney, lymph nodes, and muscle, calculated using average radiance (photons/s/cm²/sr). (F) Spleen-to-liver ratio representing relative splenic tropism, calculated using average radiance (photons/s/cm²/sr). Data are presented as individual data points with mean ± SD. Statistical analysis was performed using one-way ANOVA with Dunnett’s multiple comparisons test (* p < 0.05, ** p < 0.01, *** p < 0.001, **** p < 0.0001; ns, not significant), with all comparisons performed relative to the cholesterol LNP group.

**Figure S3. In vivo organ-level biodistribution of DLin-MC3-DMA-based LNPs incorporating bile acid-derived sterols**

(A) Representative IVIS images of excised major organs collected 4 h after intravenous administration of firefly luciferase (FLuc)-encoding mRNA-loaded LNPs (0.5 mg kg⁻¹) formulated with DLin-MC3-DMA as the ionizable lipid. Major organs (liver, spleen, heart, lung, kidney, and lymph nodes) were harvested from female BALB/c mice (6-7 weeks old, n = 3 biologically independent animals) treated with DPBS, Chol LNPs, or bile acid-derived sterol LNPs. (B–C) Quantification of total flux (photons/s) in the liver (B) and spleen (C) measured by IVIS imaging. (D) Fractional distribution of luciferase expression (%) across the major organs, calculated using average radiance (photons/s/cm²/sr). (E) Spleen-to-liver ratio of luciferase expression, representing relative splenic tropism, calculated using average radiance (photons/s/cm²/sr). Data are presented as individual data points with mean ± SD. Statistical analysis was performed using one-way ANOVA with Dunnett’s multiple comparisons test (*p < 0.05, ***p < 0.001, ****p < 0.0001; ns, not significant), with all comparisons performed relative to the Chol LNP group.

**Figure S4. Physicochemical characterization of cholesterol and unmodified bile acid LNPs**

Physicochemical profiles of LNPs formulated with unmodified bile acids under identical conditions. LNPs were prepared using cholesterol (Chol) or unmodified bile acids (LCA, DCA, CA) as the sterol component under the same formulation and mixing conditions. (A) Encapsulation efficiency (n = 3) and (B) Z-average (n = 4), (C) Zeta potential (n = 4), and (D) PDI (n = 4) were measured to compare formulation performance and particle properties across sterol types. Data are shown as individual data points with mean ± SD.

**Figure S5. Structural comparison of cholesterol and bile acids**

Red highlights indicate the A/B ring fusion type (cis or trans), and blue highlights indicate the terminal group type (alkyl group or carboxyl group).

**Figure S6. Zeta potential profiles of bile acid and bile acid-derived sterol LNPs**

3D bar plot summarizing the Zeta potential (mV) values of LNPs across the bile acid and bile acid-derived sterol LNPs.


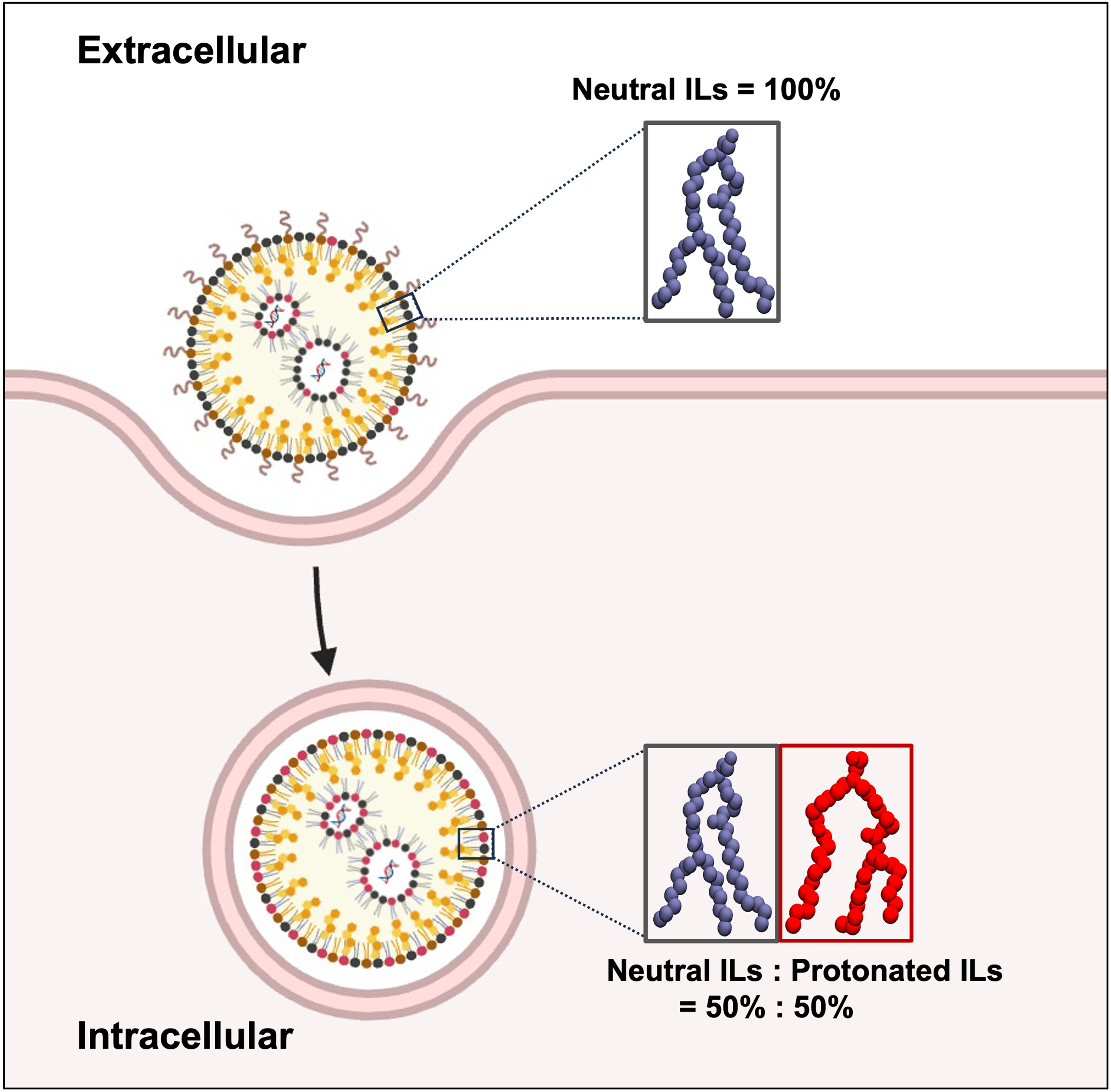


**Figure S7. Schematic of Ionizable-Lipid Protonation States in Extracellular vs Endosomal Environments**

Schematic of an extracellular environment with neutral ionizable lipids and an endosomal environment with a 50:50 mixture of neutral and protonated ionizable lipids.

| **LCA** | **DCA** | **CA** |
| --- | --- | --- |
| 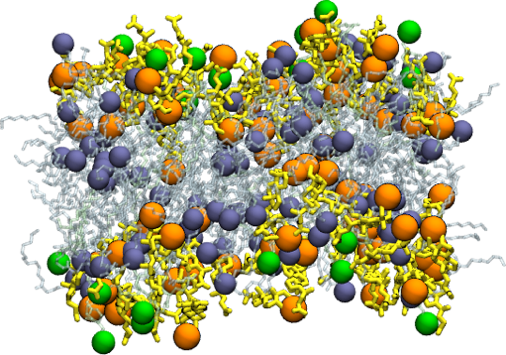 | 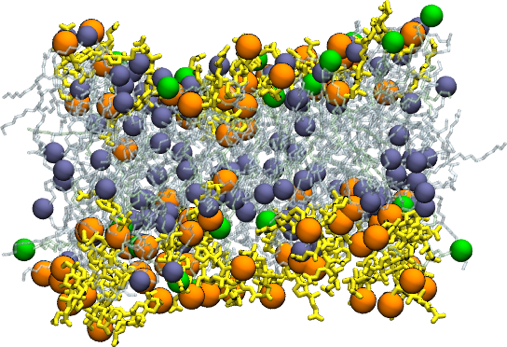 | 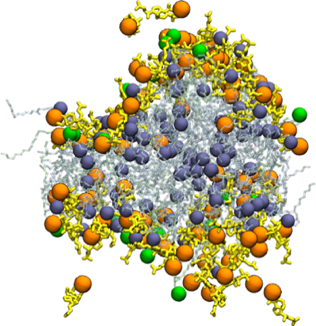 |
| **LCA-4** | **DCA-4** | **CA-4** |
| 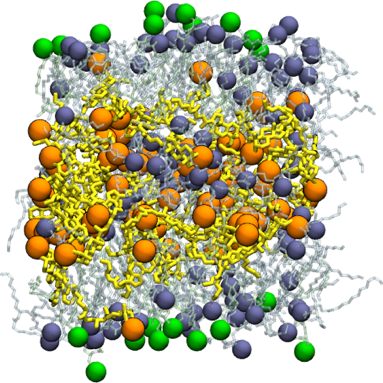 | 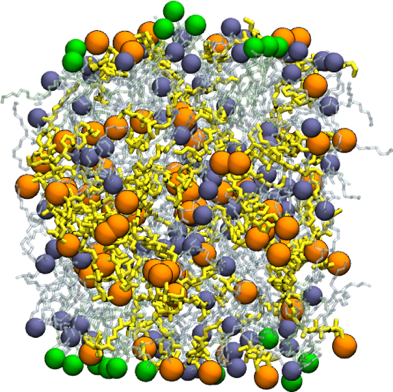 | 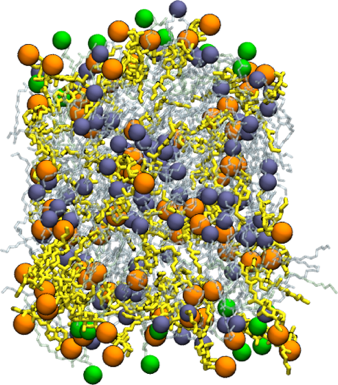 |
| **LCA-16** | **DCA-16** | **CA-16** |
| 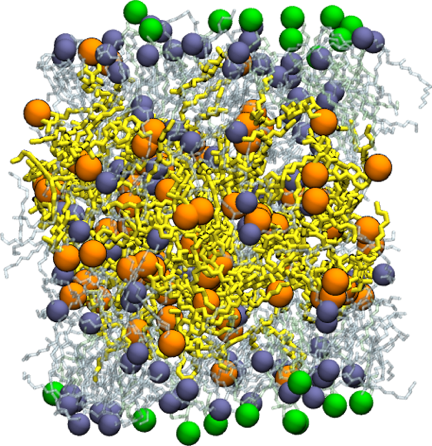 | 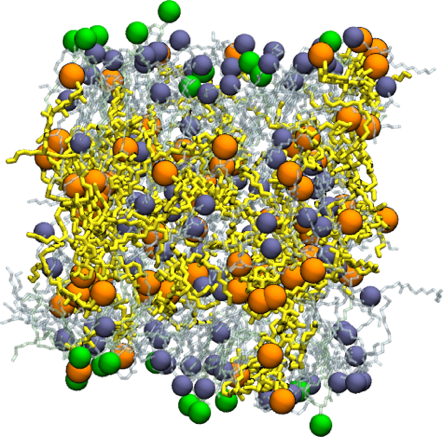 | 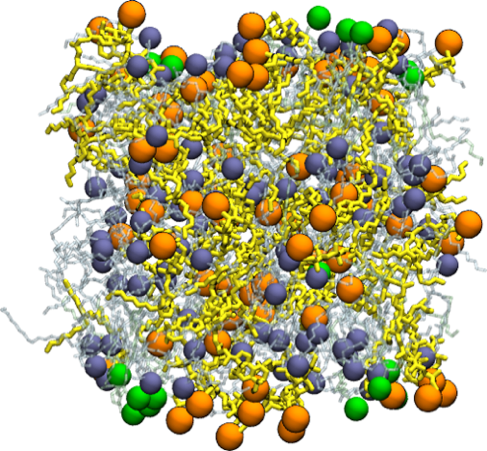 |
| **LCA-20** | **DCA-20** | **CA-20** |
| 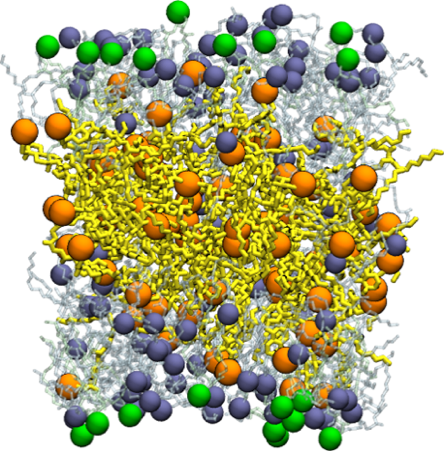 | 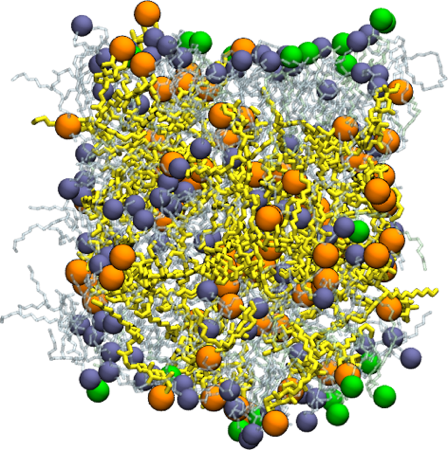 | 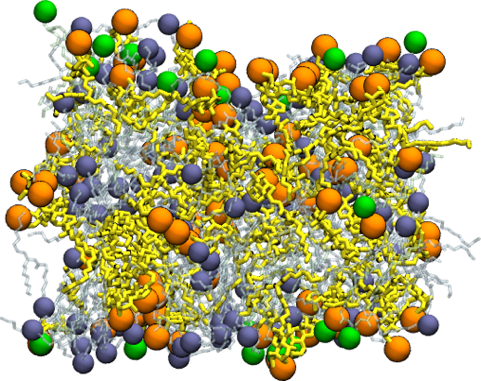 |

**Figure S8. Neutral-condition MD snapshots of bile acid and bile acid-derived sterol LNP bilayers**

MD snapshots of all bile acid and bile acid-derived sterol LNP bilayer systems obtained after 1.5 µs simulations under neutral conditions. Headgroups were highlighted by rendering them as enlarged beads: sterol heads in orange, ionizable lipid heads in ice-blue, and DSPC heads in green. Non-head atoms were de-emphasized (sterol non-head atoms in yellow; all other lipid non-head atoms in gray) to facilitate inspection of the relative headgroup positions of sterols and other lipids.

| **LCA** | **DCA** | **CA** |
| --- | --- | --- |
| **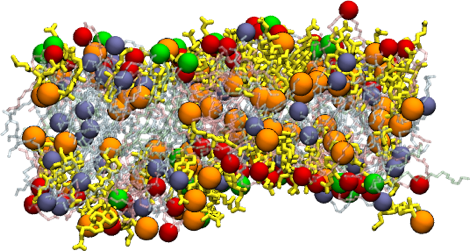** | **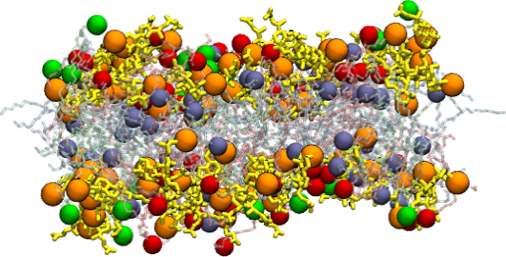** | **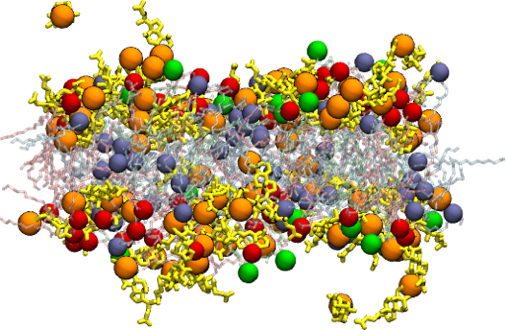** |
| **LCA-4** | **DCA-4** | **CA-4** |
| **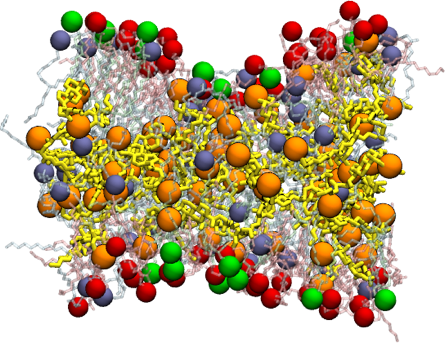** | **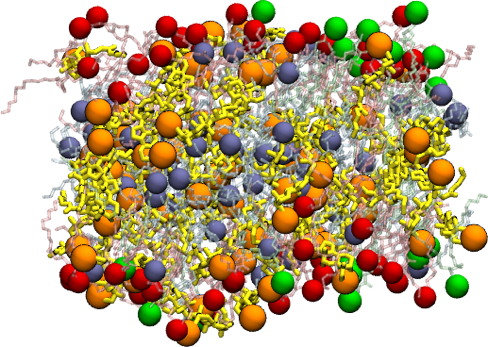** | **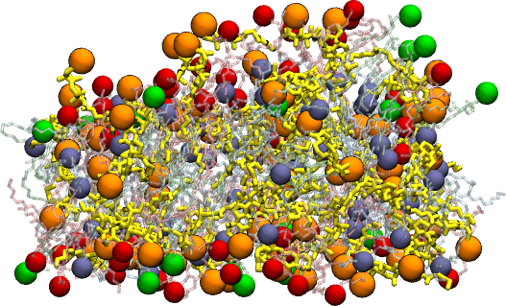** |
| **LCA-16** | **DCA-16** | **CA-16** |
| **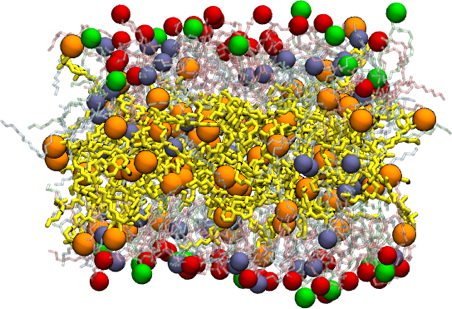** | **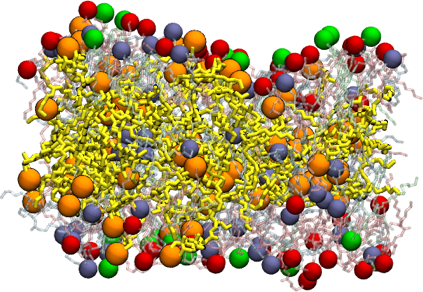** | **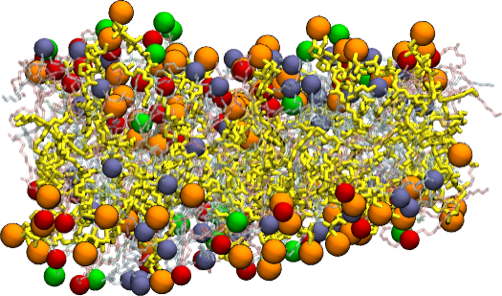** |
| **LCA-20** | **DCA-20** | **CA-20** |
| **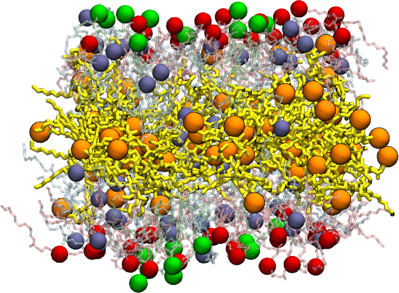** | **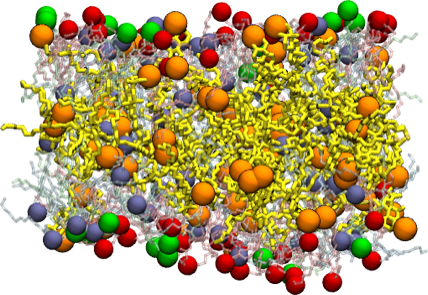** | **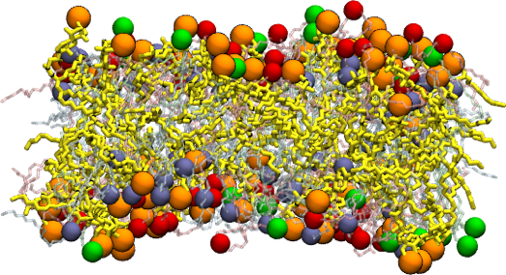** |

**Figure S9. Protonated-condition MD snapshots of bile acid and bile acid-derived sterol LNP bilayers**

MD snapshots of bile acid and bile acid-derived sterol LNP bilayer systems obtained after 1.5 µs simulations under protonated conditions. Headgroup representations follow Figure S8; additionally, protonated ionizable lipid headgroups are rendered in red.

| **LCA** | **DCA** | **CA** |
| --- | --- | --- |
| **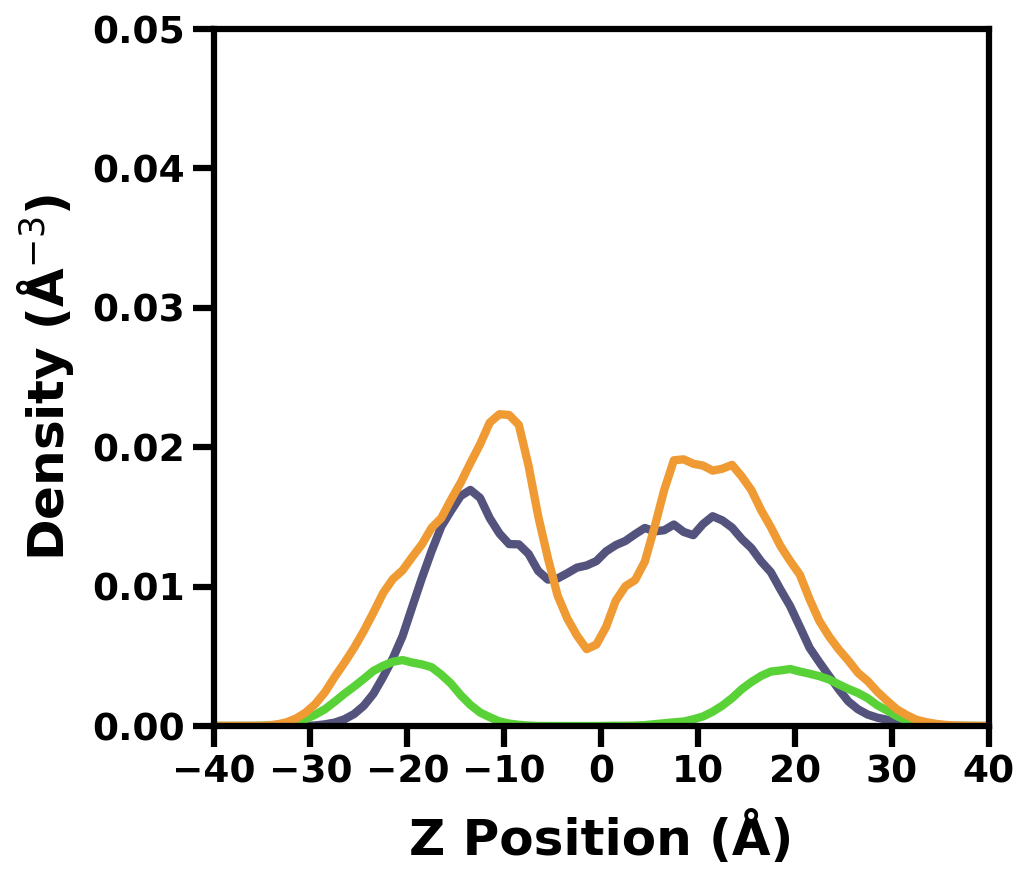** | **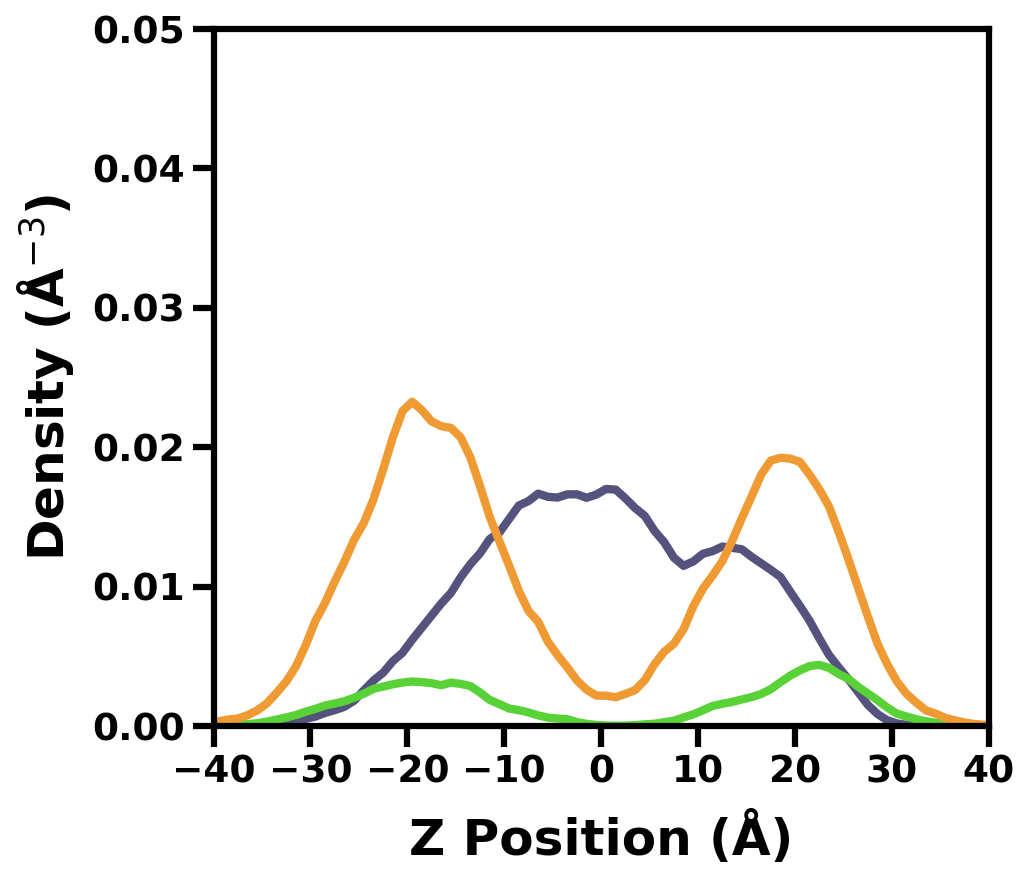** | **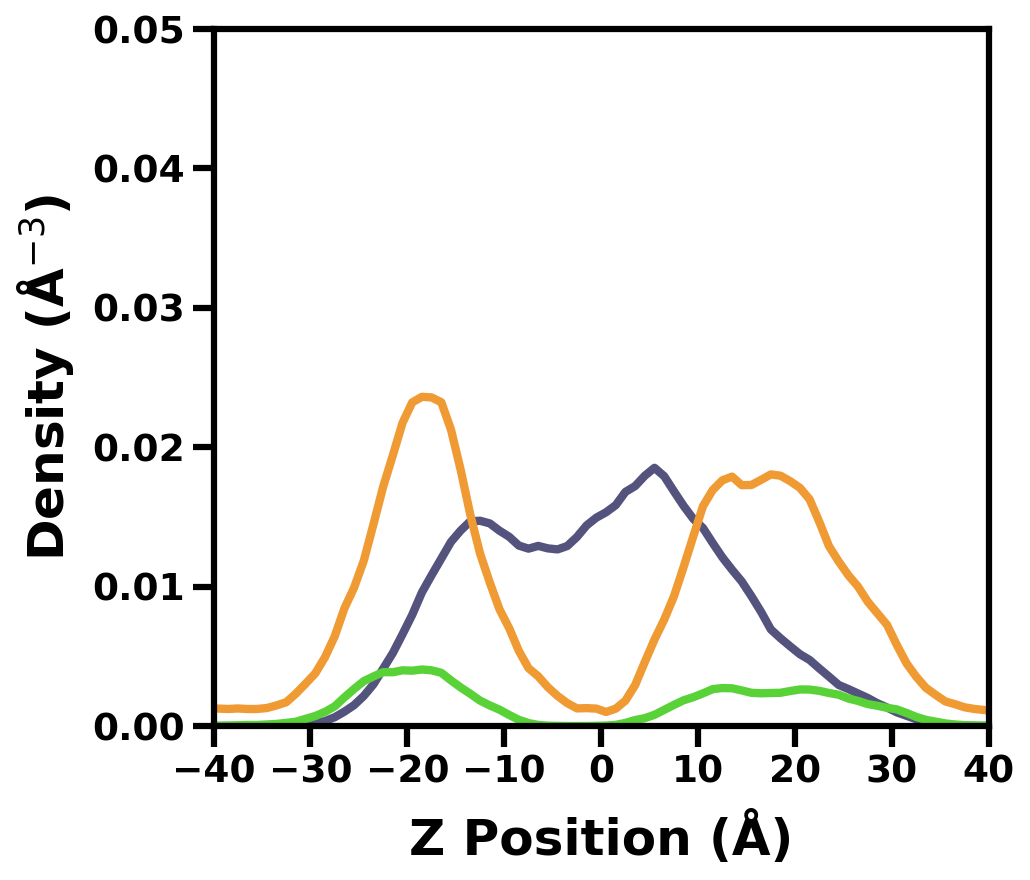** |
| **LCA-4** | **DCA-4** | **CA-4** |
| **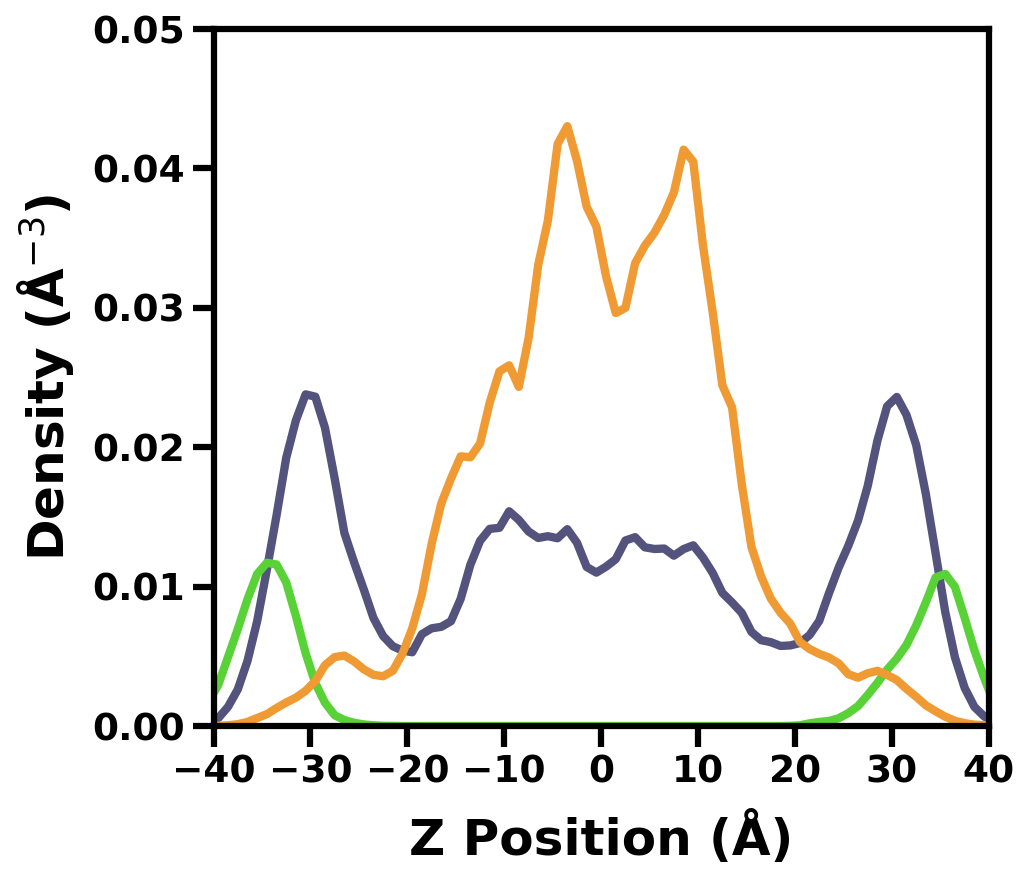** | **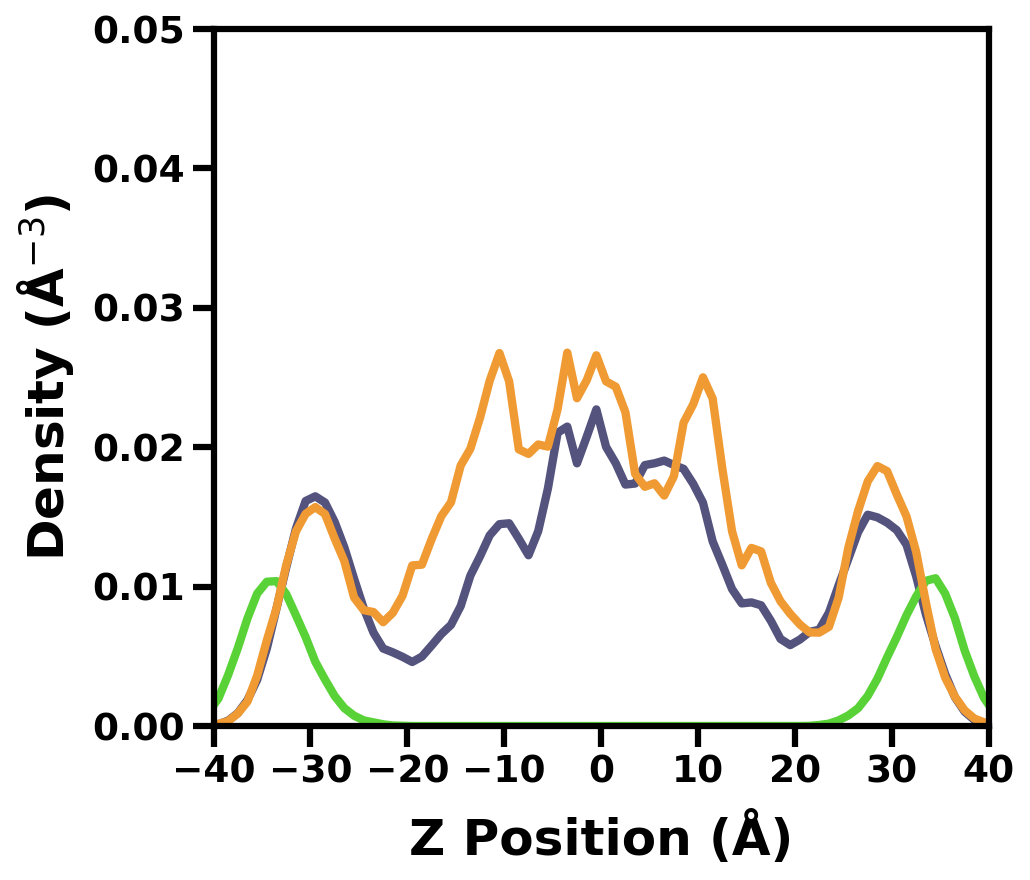** | **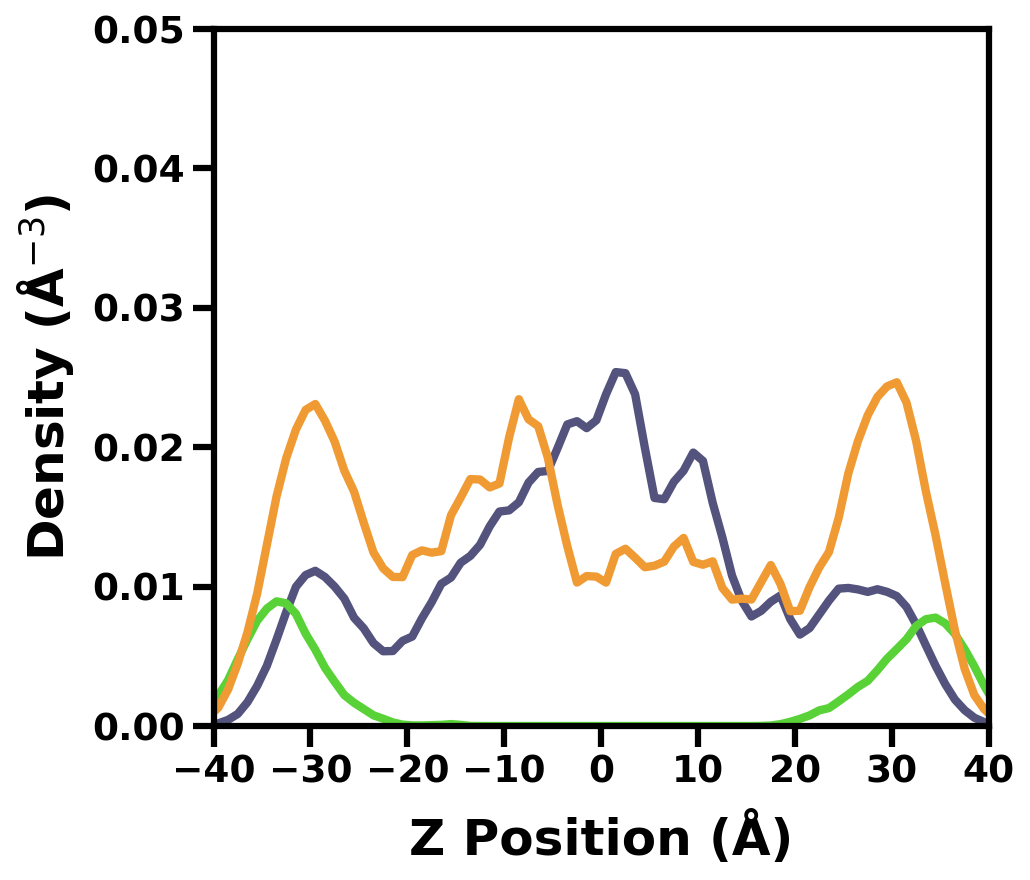** |
| **LCA-16** | **DCA-16** | **CA-16** |
| **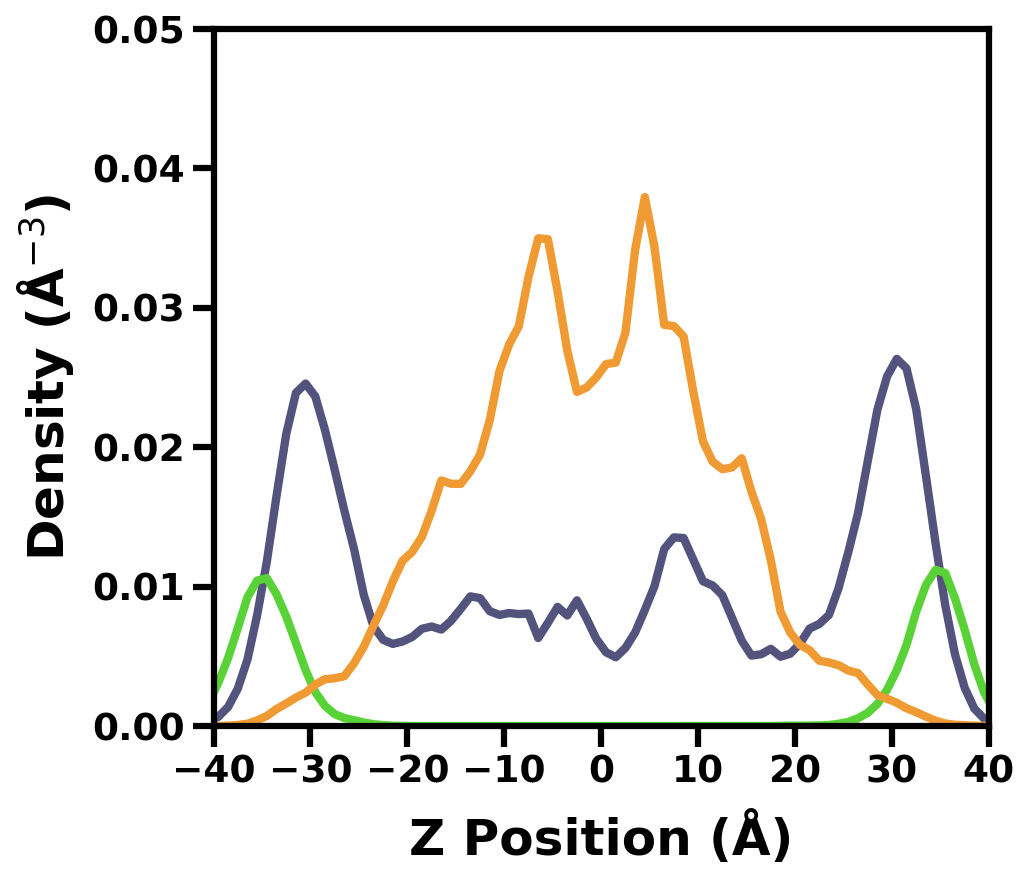** | **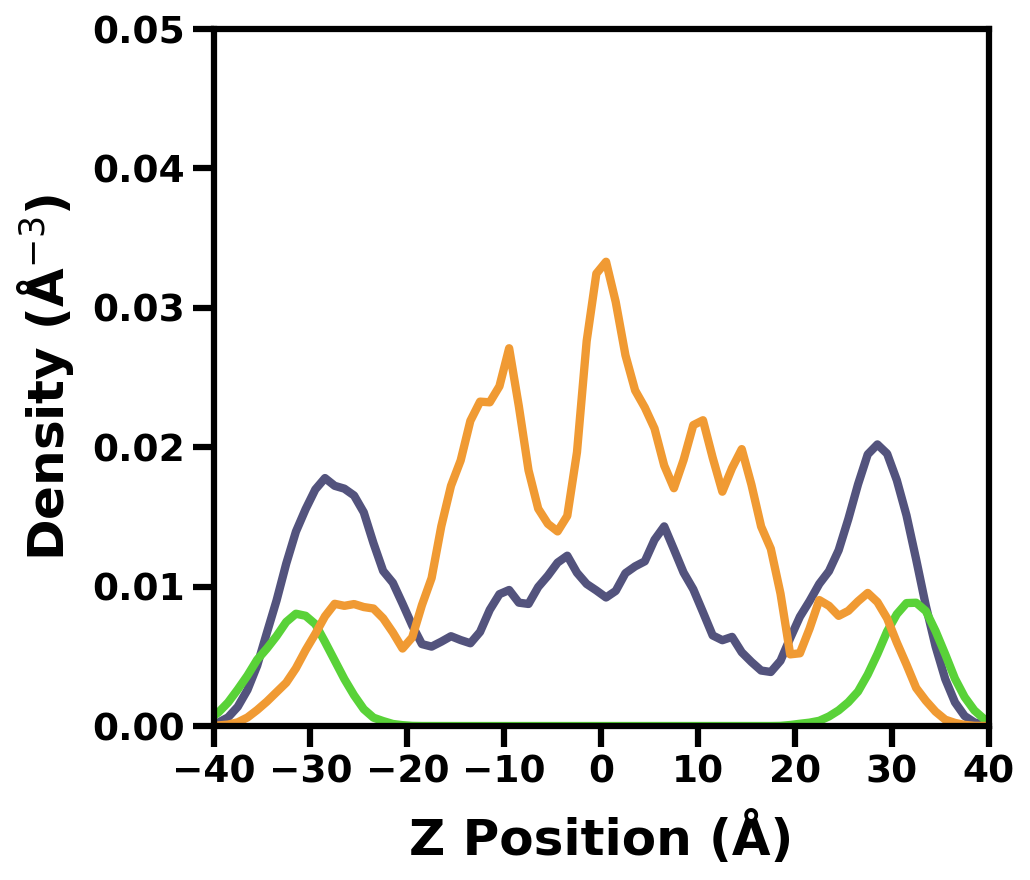** | **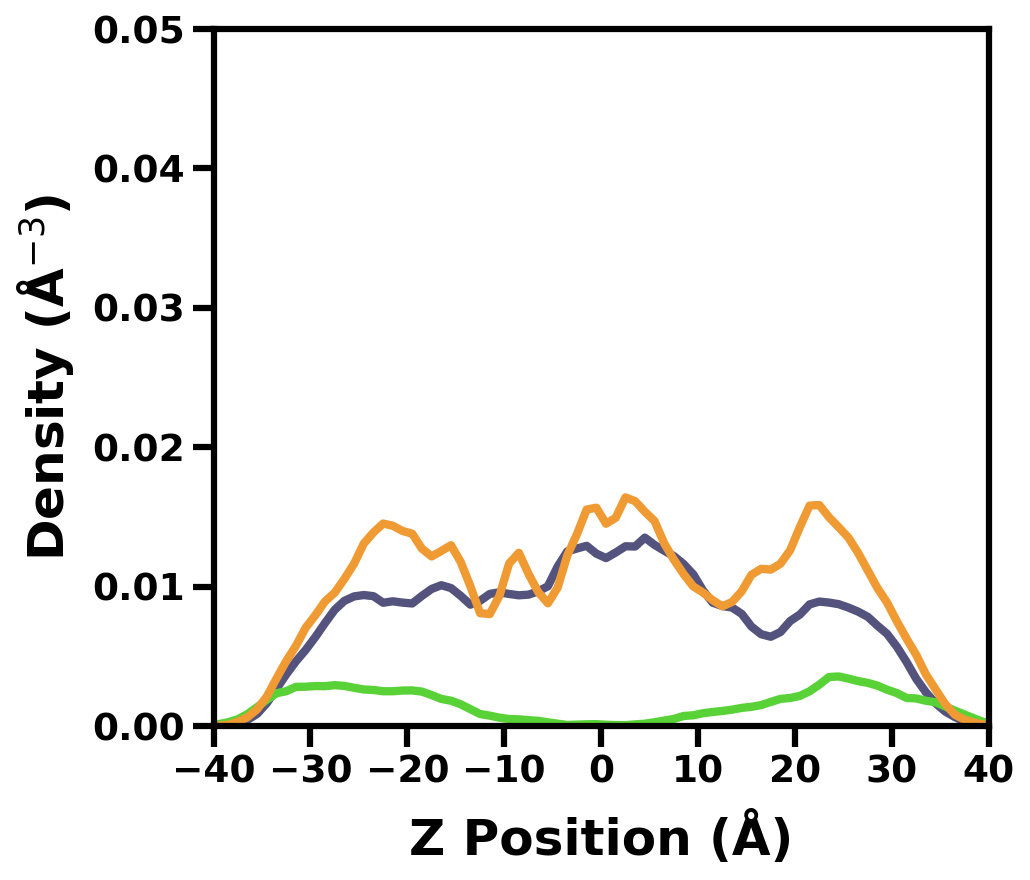** |
| **LCA-20** | **DCA-20** | **CA-20** |
| **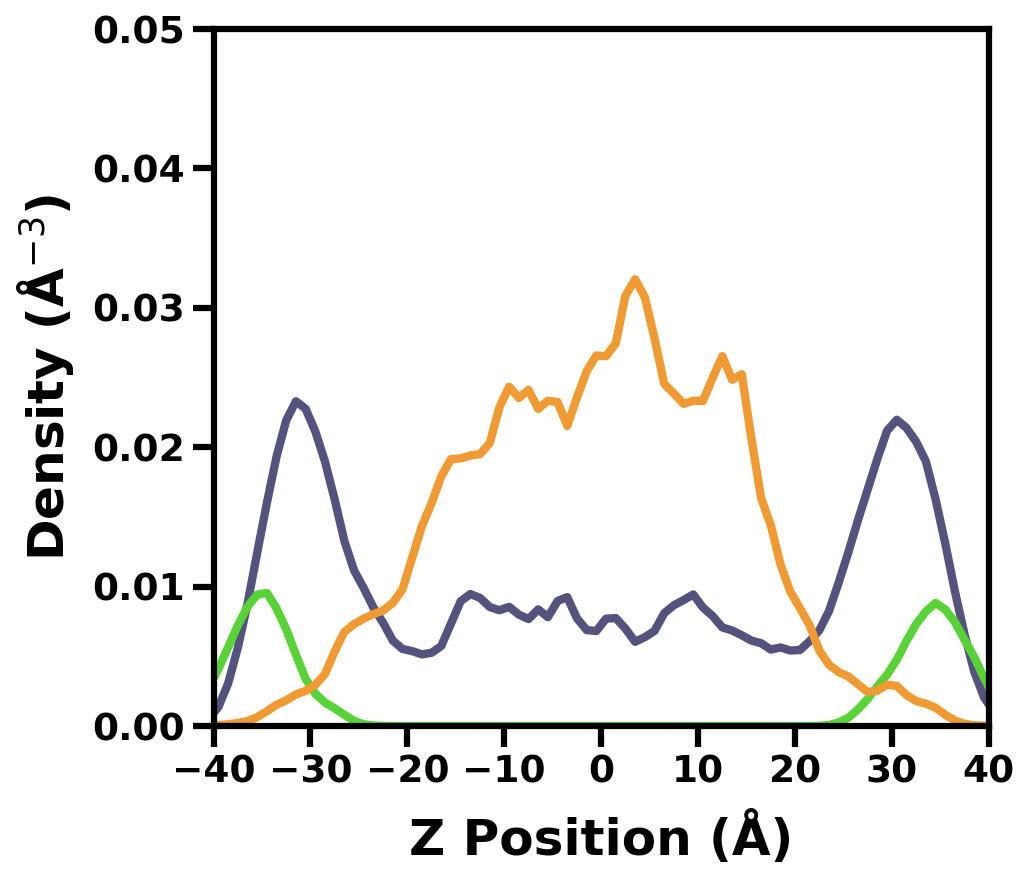** | **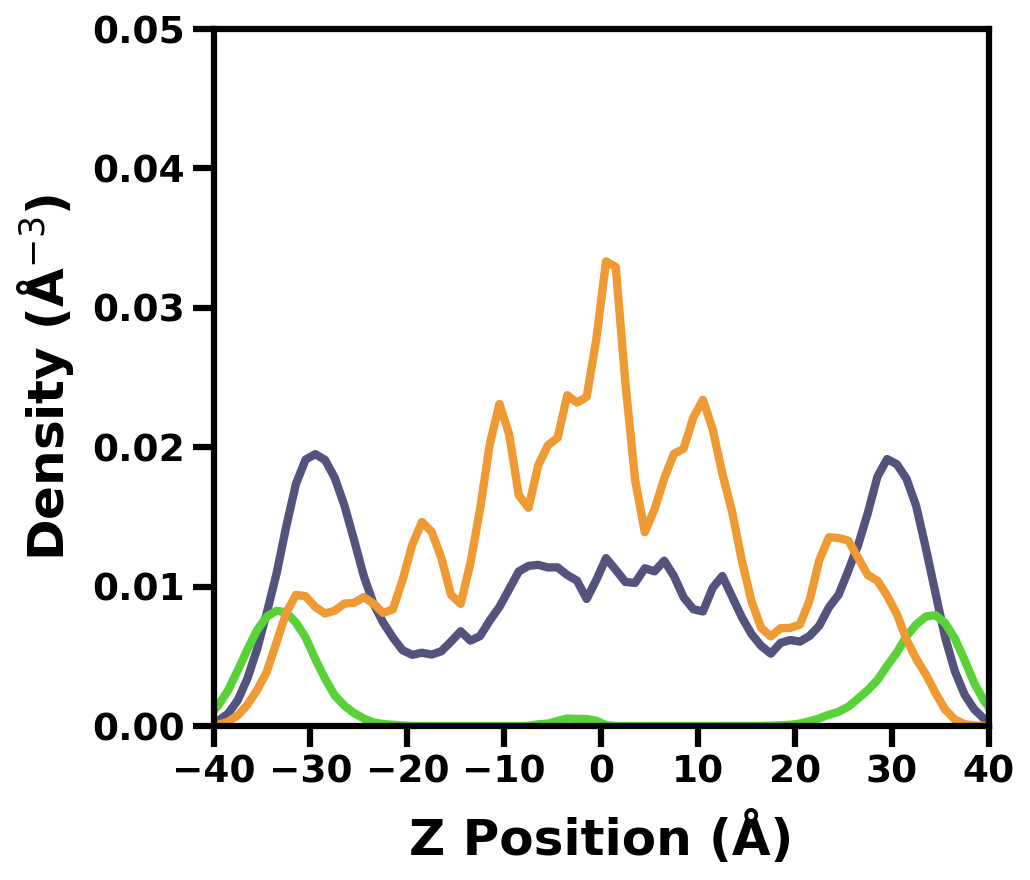** | **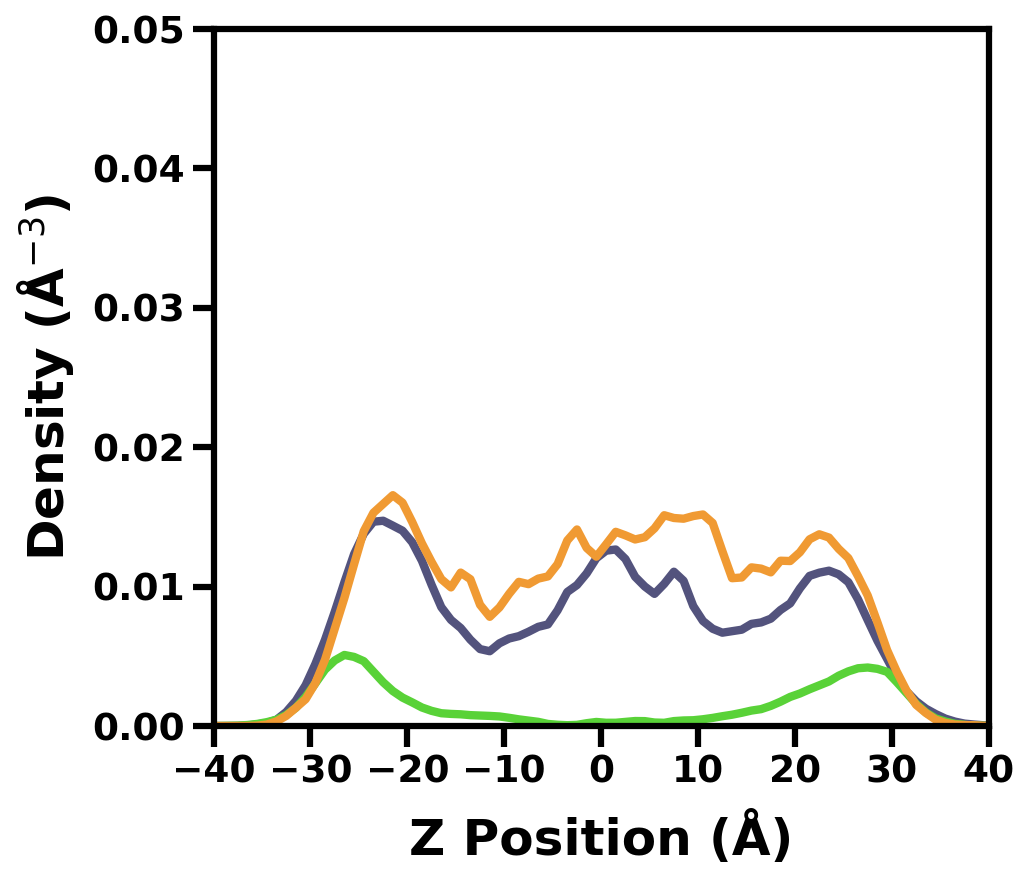** |

**Figure S10. Neutral-condition Z-density profiles of bile acid and bile acid-derived sterol LNP bilayer components**

Z-density profiles of the ionizable lipid (SM-102), DSPC, and sterol along Z-axis under neutral conditions for all bile acid and bile acid-derived sterol LNP bilayer systems: orange for sterols, green for DSPC, and ice-blue for neutral ionizable lipids.

| **LCA** | **DCA** | **CA** |
| --- | --- | --- |
| **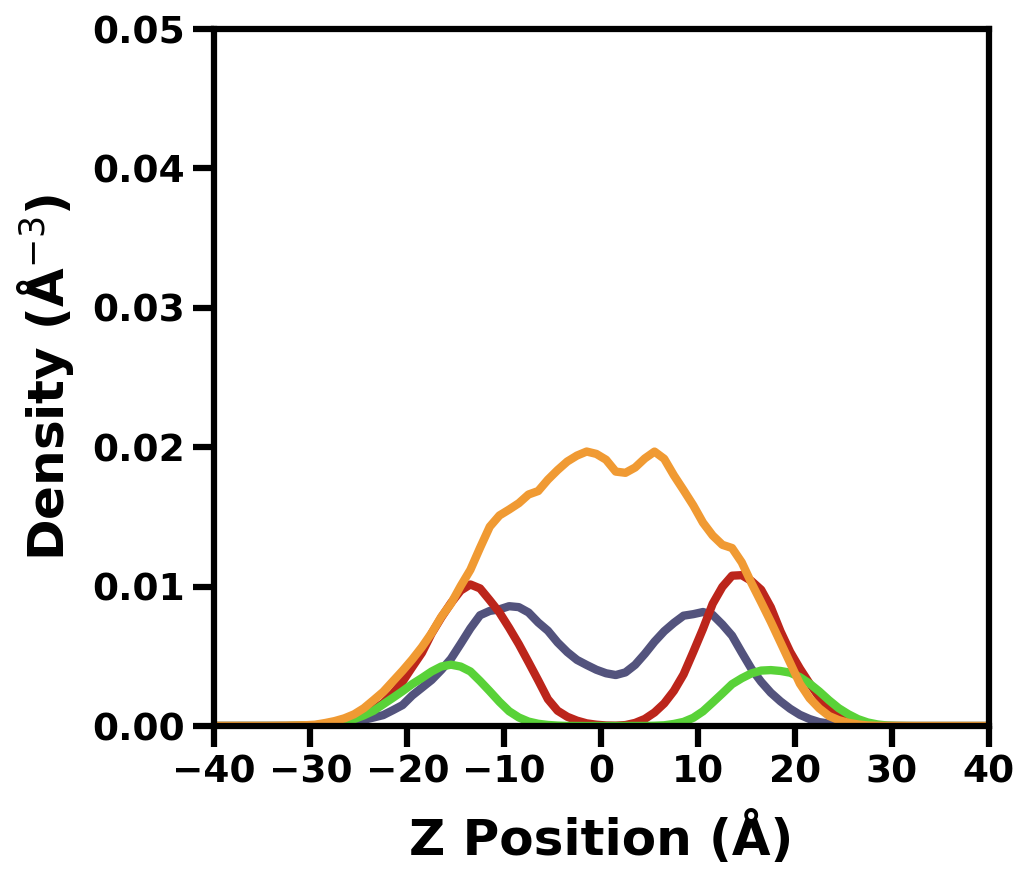** | **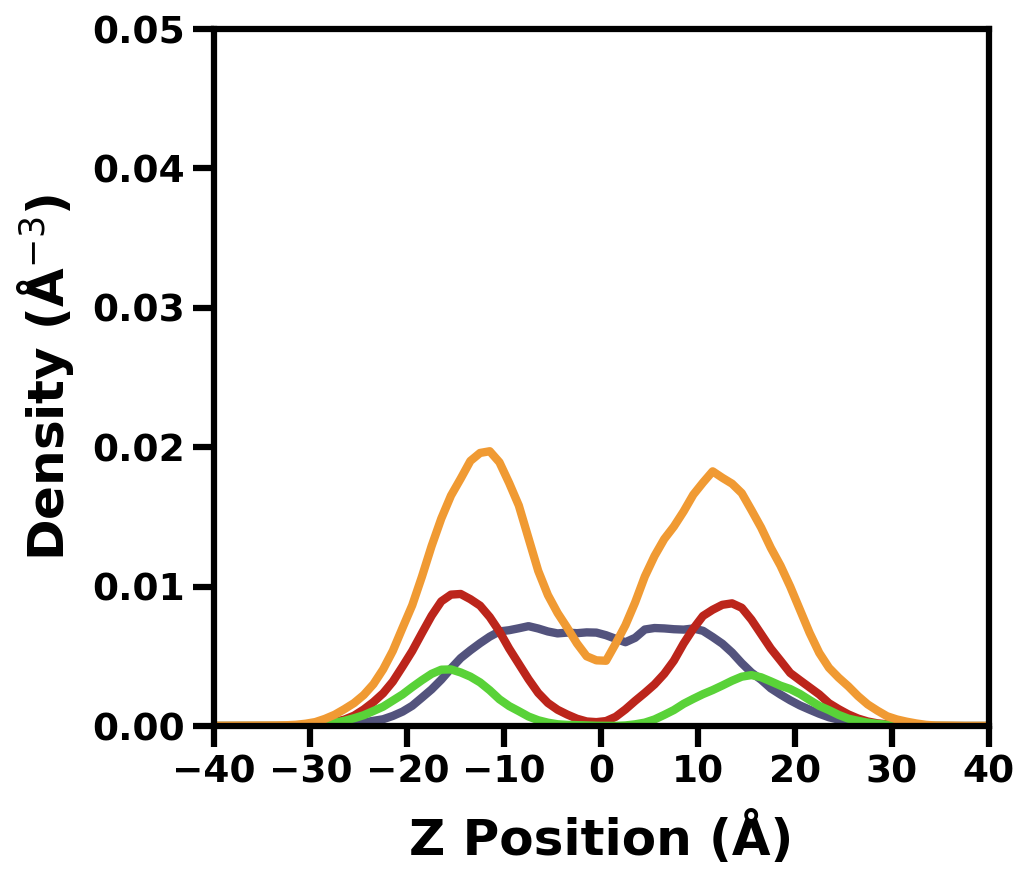** | **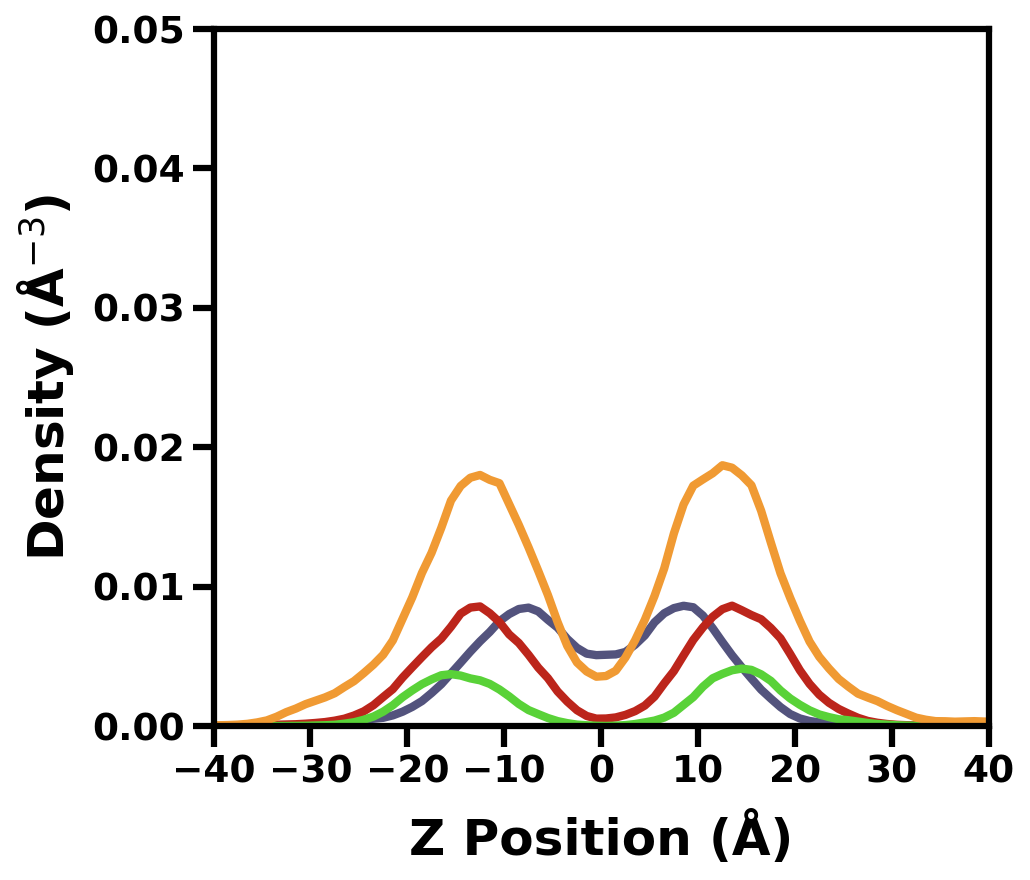** |
| **LCA-4** | **DCA-4** | **CA-4** |
| **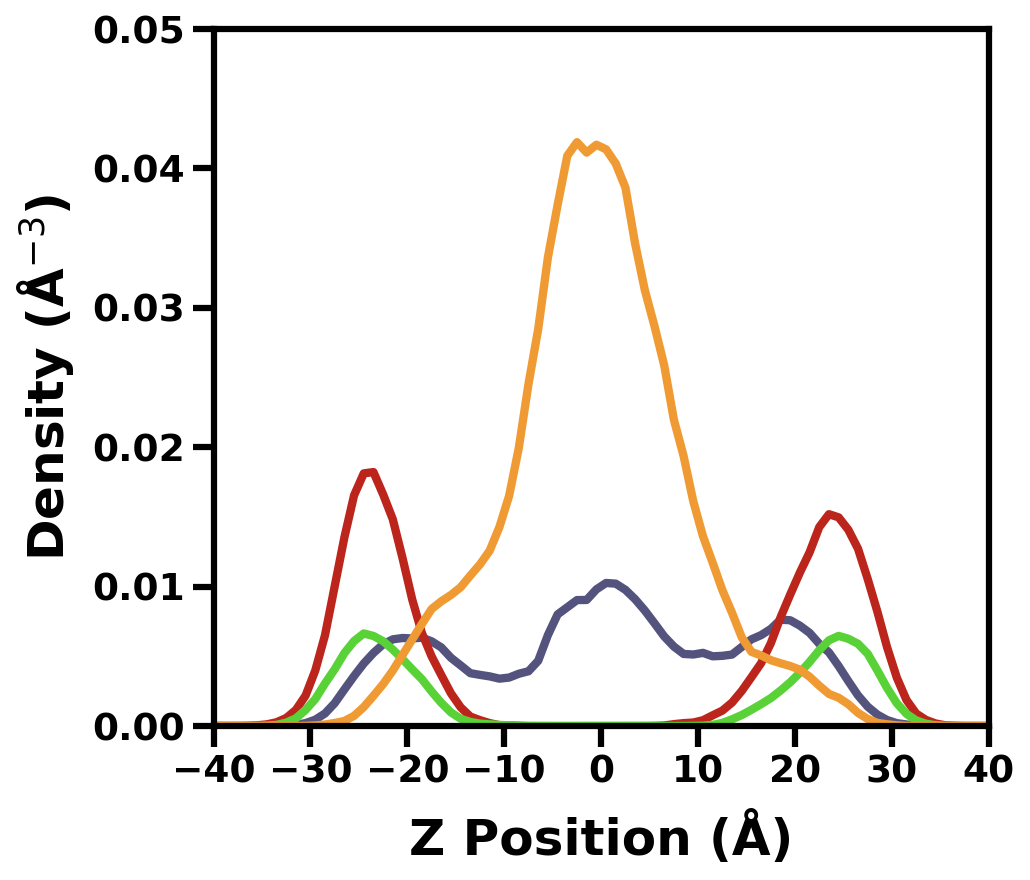** | **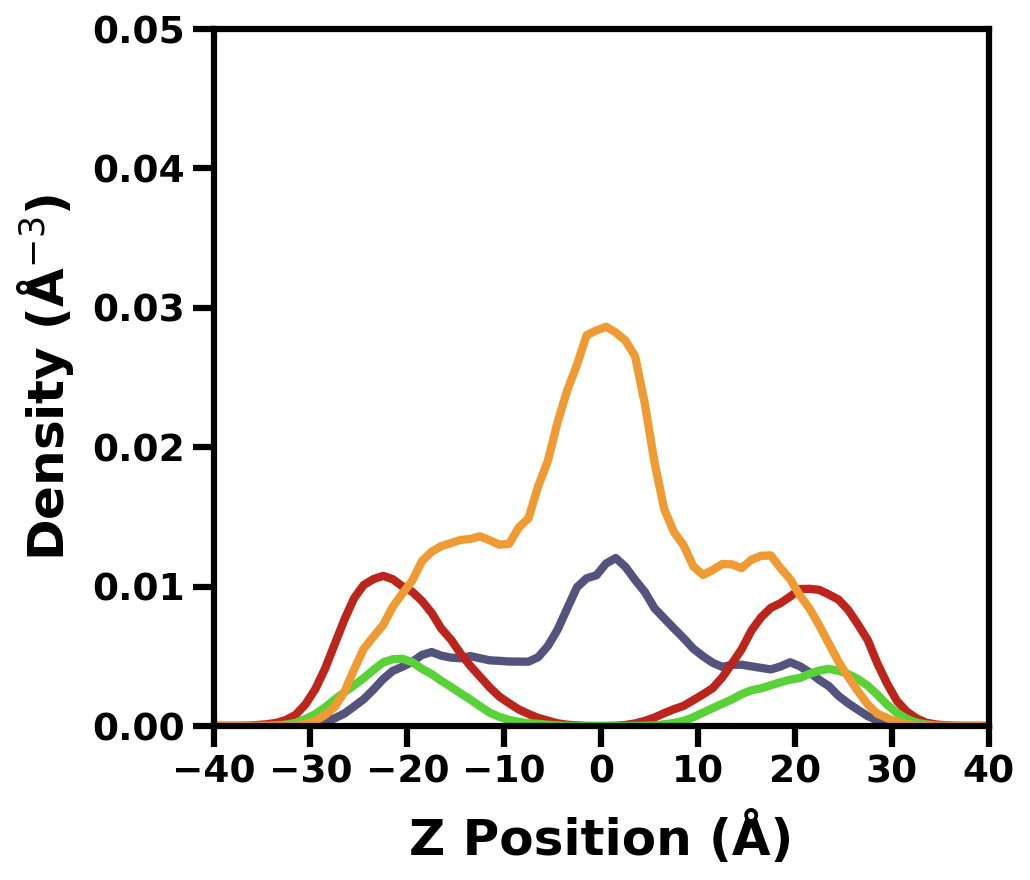** | **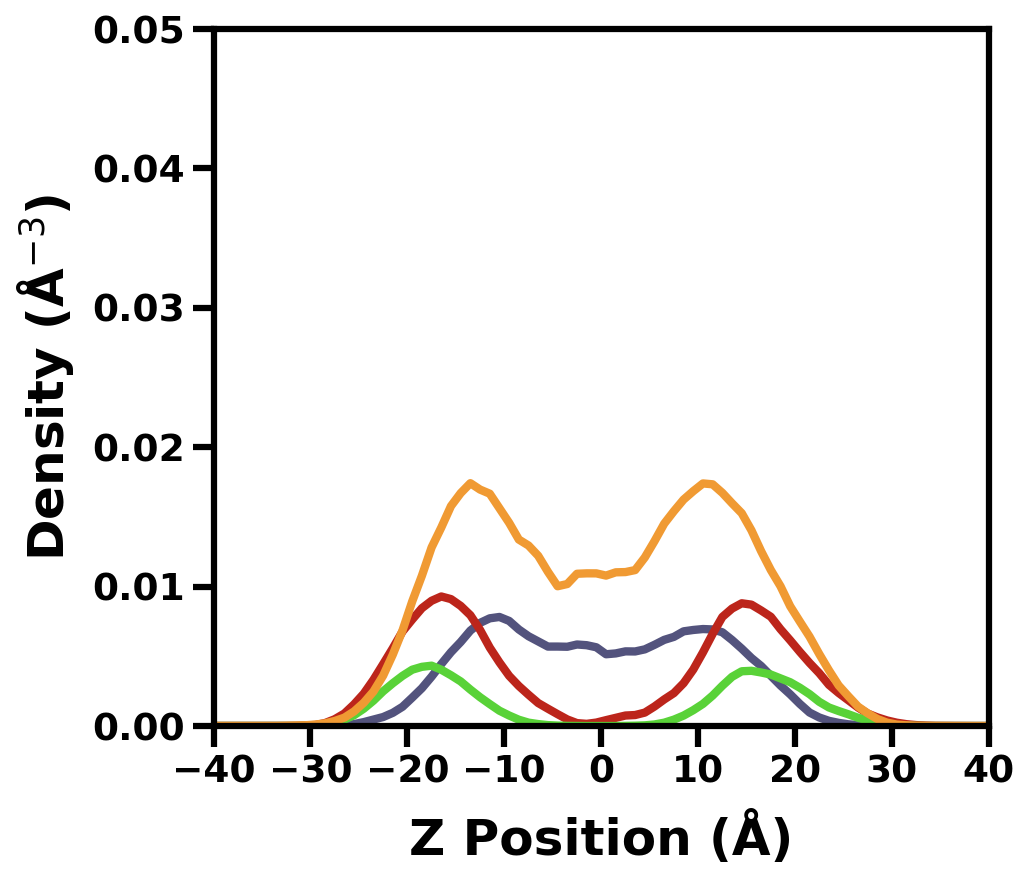** |
| **LCA-16** | **DCA-16** | **CA-16** |
| **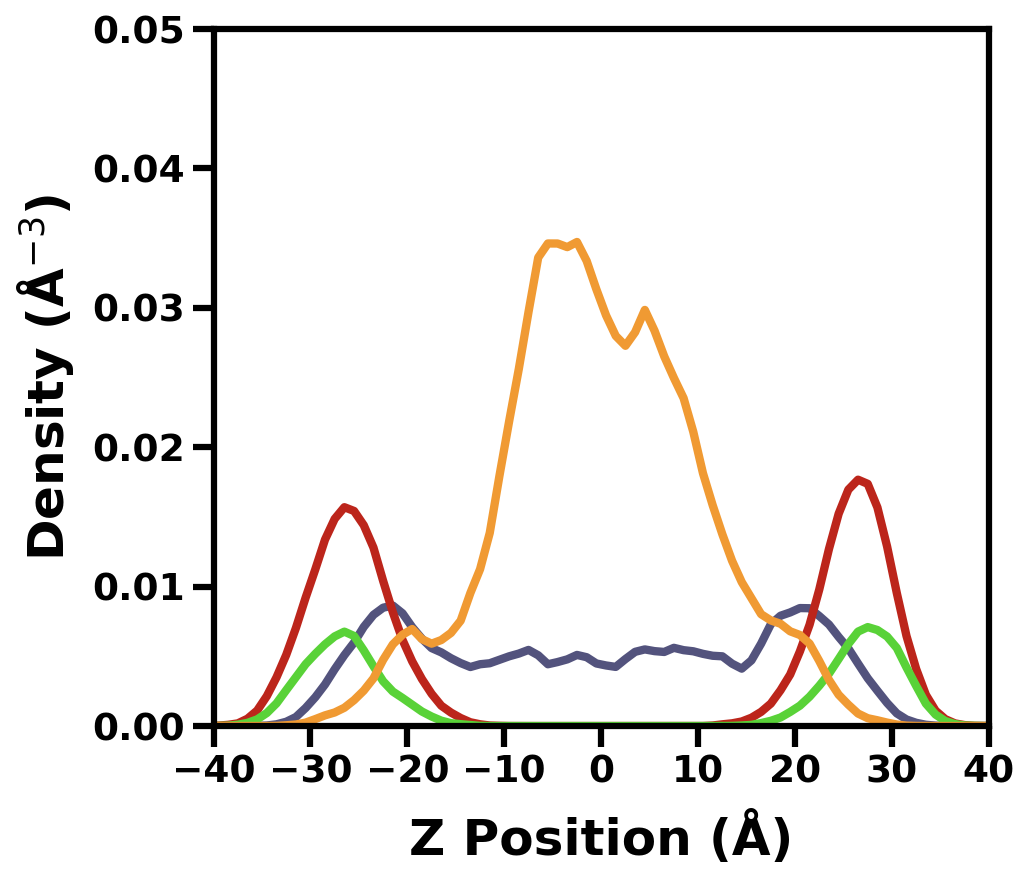** | **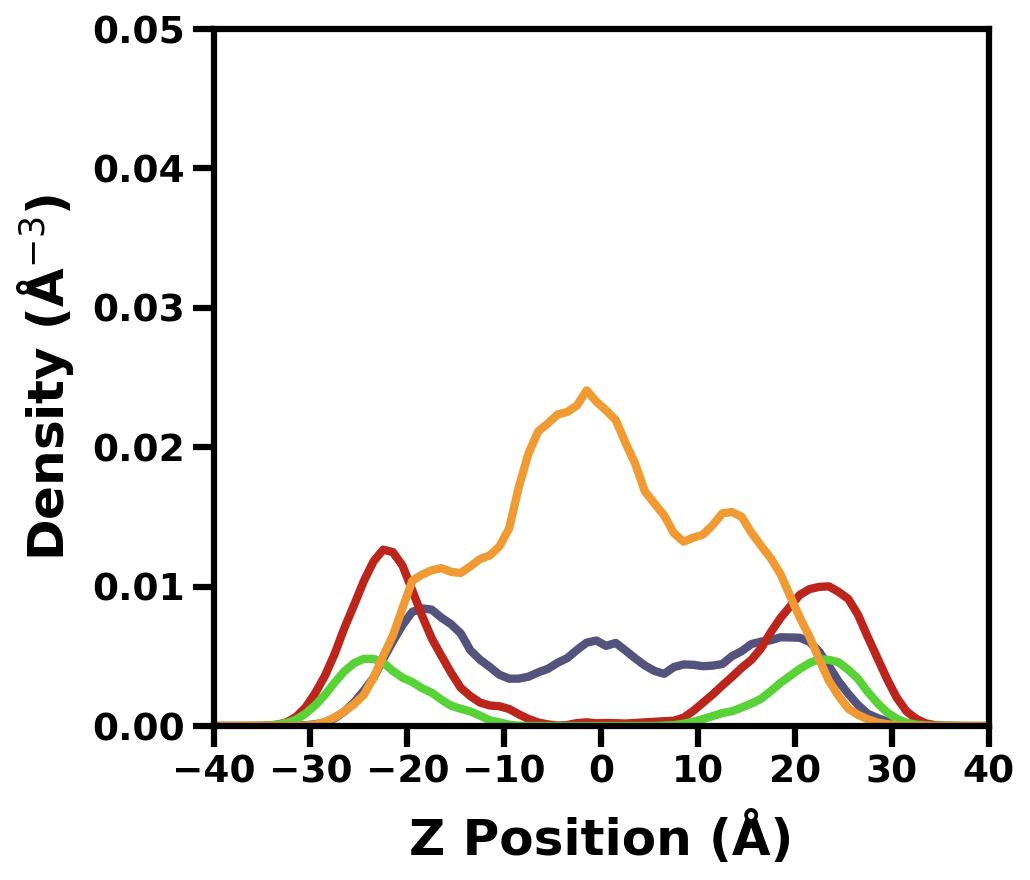** | **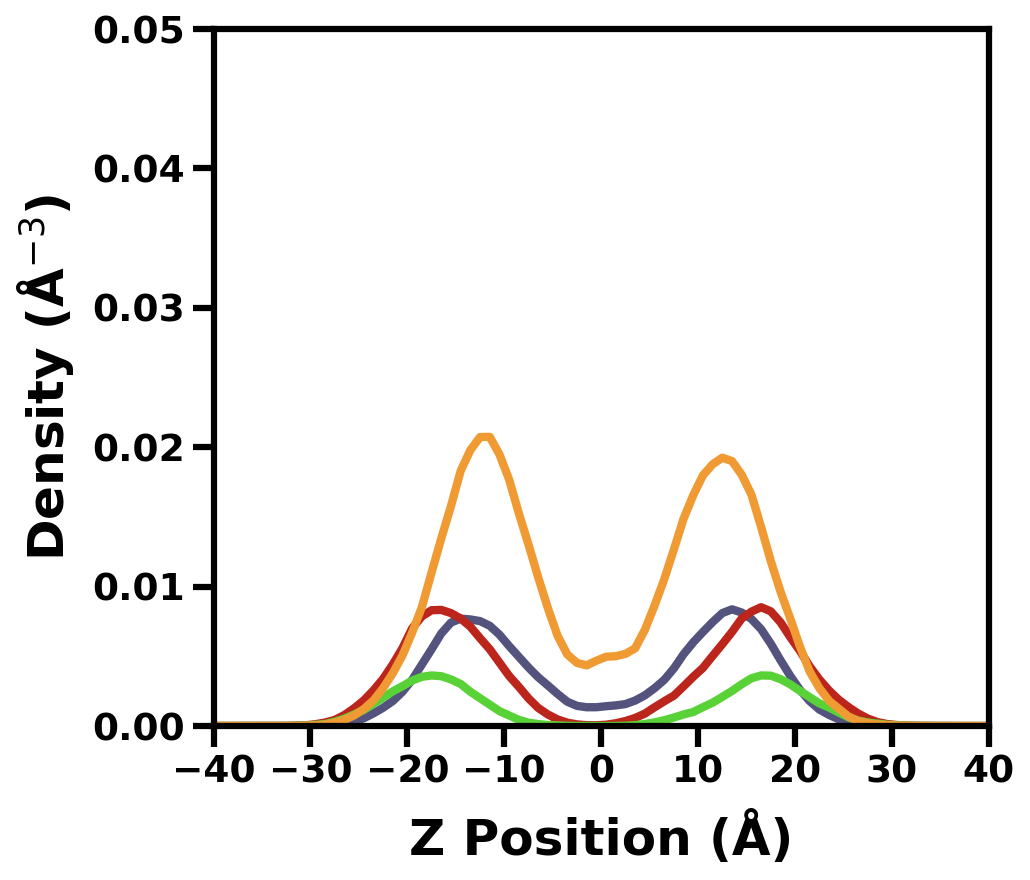** |
| **LCA-20** | **DCA-20** | **CA-20** |
| **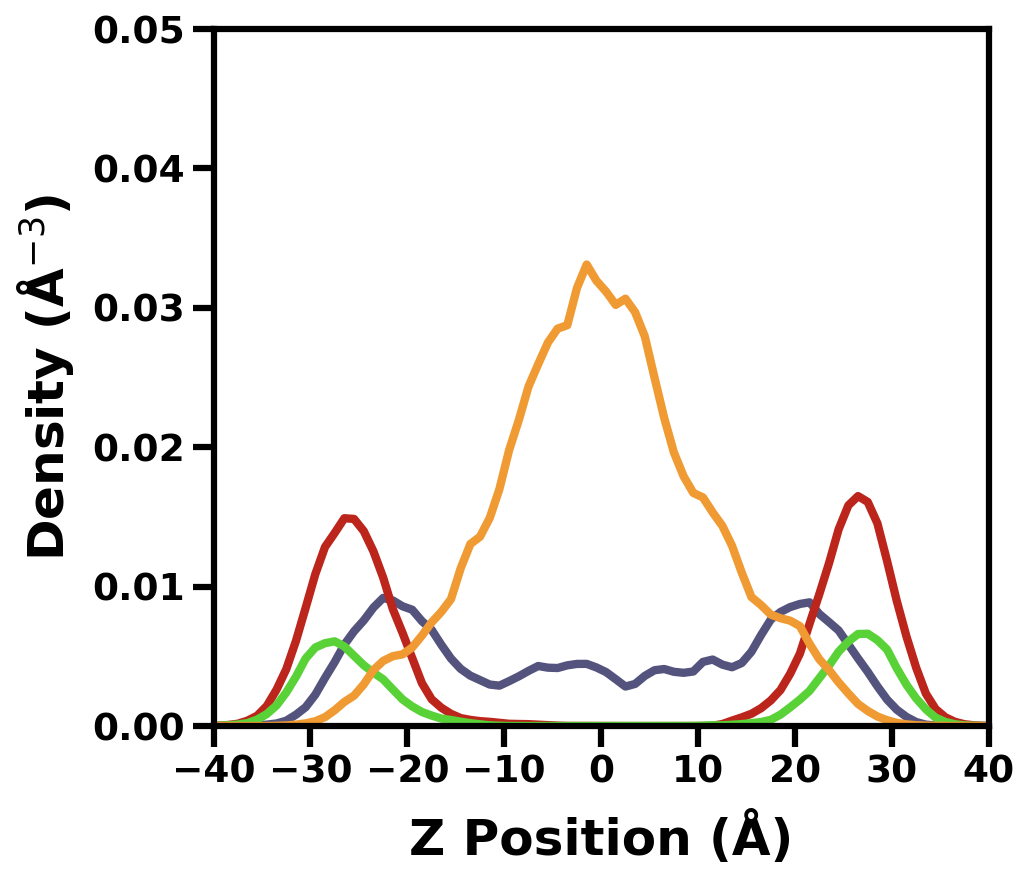** | **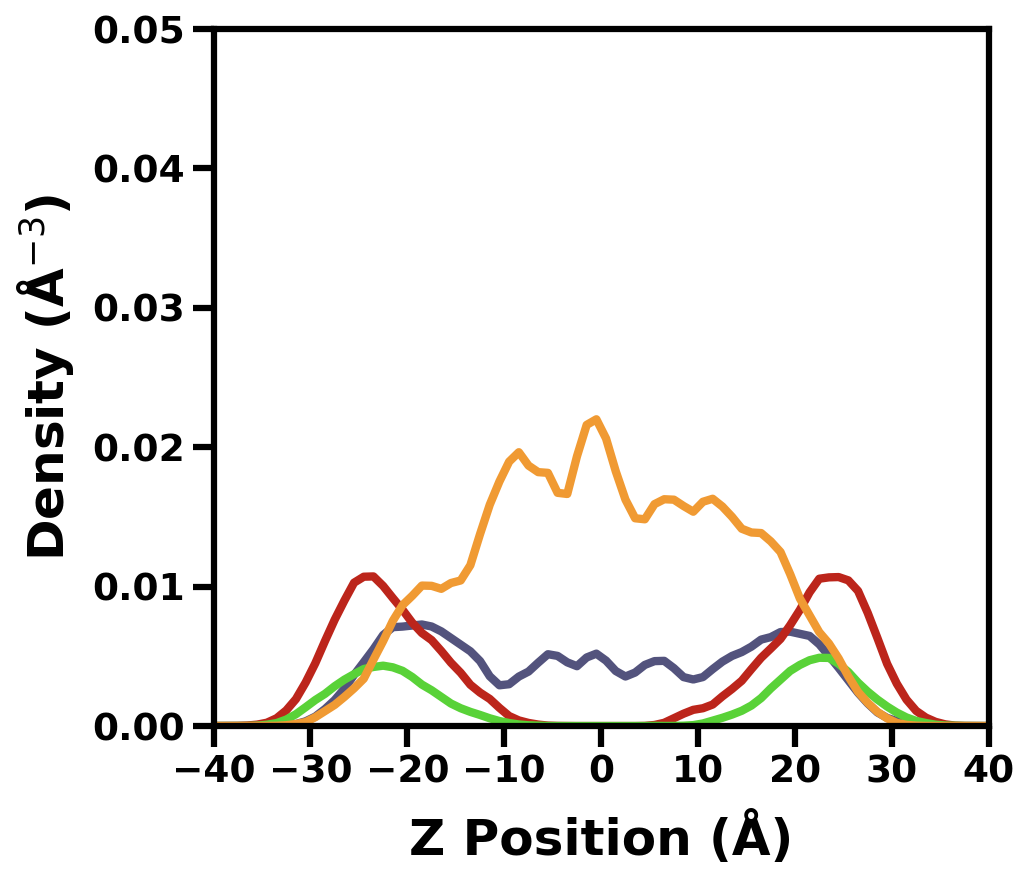** | **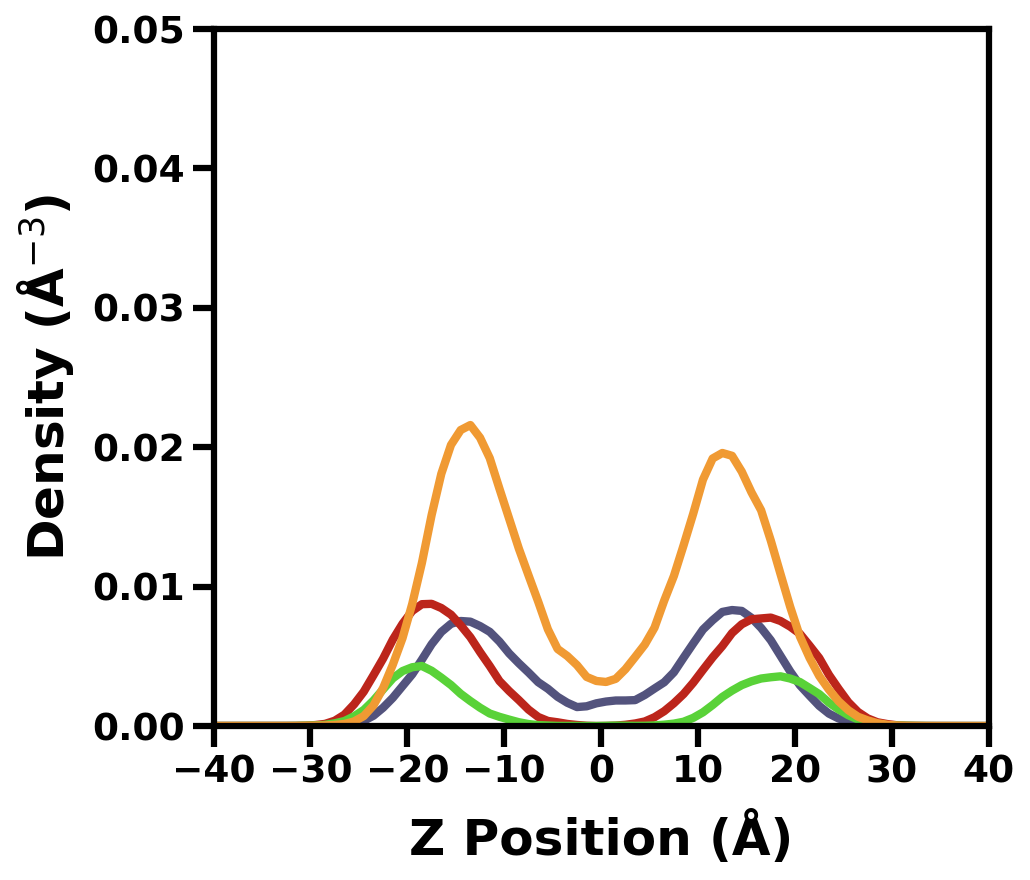** |

**Figure S11. Protonated-condition Z-density profiles of bile acid and bile acid-derived sterol LNP bilayer components**

Z-density profiles of the ionizable lipid (SM-102), DSPC, and sterol along Z-axis under protonated conditions for all bile acid and bile acid-derived sterol LNP bilayer systems: orange for sterols, green for DSPC, ice-blue for neutral ionizable lipids, and red for protonated ionizable lipids.

**A B**

**Neutral condition Protonated condition**

**Chol Chol**

**Figure S12.** **Chol LNP bilayers: snapshots and Z-density profiles (neutral vs protonated)**

Representative snapshots (top) and Z-density profiles (bottom) of the ionizable lipid (SM-102), DSPC, and cholesterol along the Z-axis for Chol LNP bilayer systems under neutral (A) and protonated (B) conditions: orange for cholesterol, green for DSPC, ice-blue for neutral ionizable lipids, and red for protonated ionizable lipids.

**Figure S13. Cryo-TEM characterization of particle size distribution and morphological irregularity in bile acid-derived sterol LNPs**

Representative cryo-TEM images of 13 distinct LNP formulations prepared with Chol, three unmodified bile acids, and nine bile acid-derived sterols, highlighting differences in particle size distribution and vesicular morphology. White arrows denote morphological irregularities, including surface protrusions, non-spherical morphology, emulsion-like structures, and bleb-like structures. Scale bar = 100 nm.

**Figure S14. High-magnification cryo-TEM analysis of membrane morphology and bilayer proportion in LNPs**

Representative high-magnification cryo-TEM images of 13 distinct LNP formulations highlighting membrane morphology and bilayer structures. Scale bar = 50 nm.

| **A Neutral Condition** | **B Protonated Condition** |
| --- | --- |
| **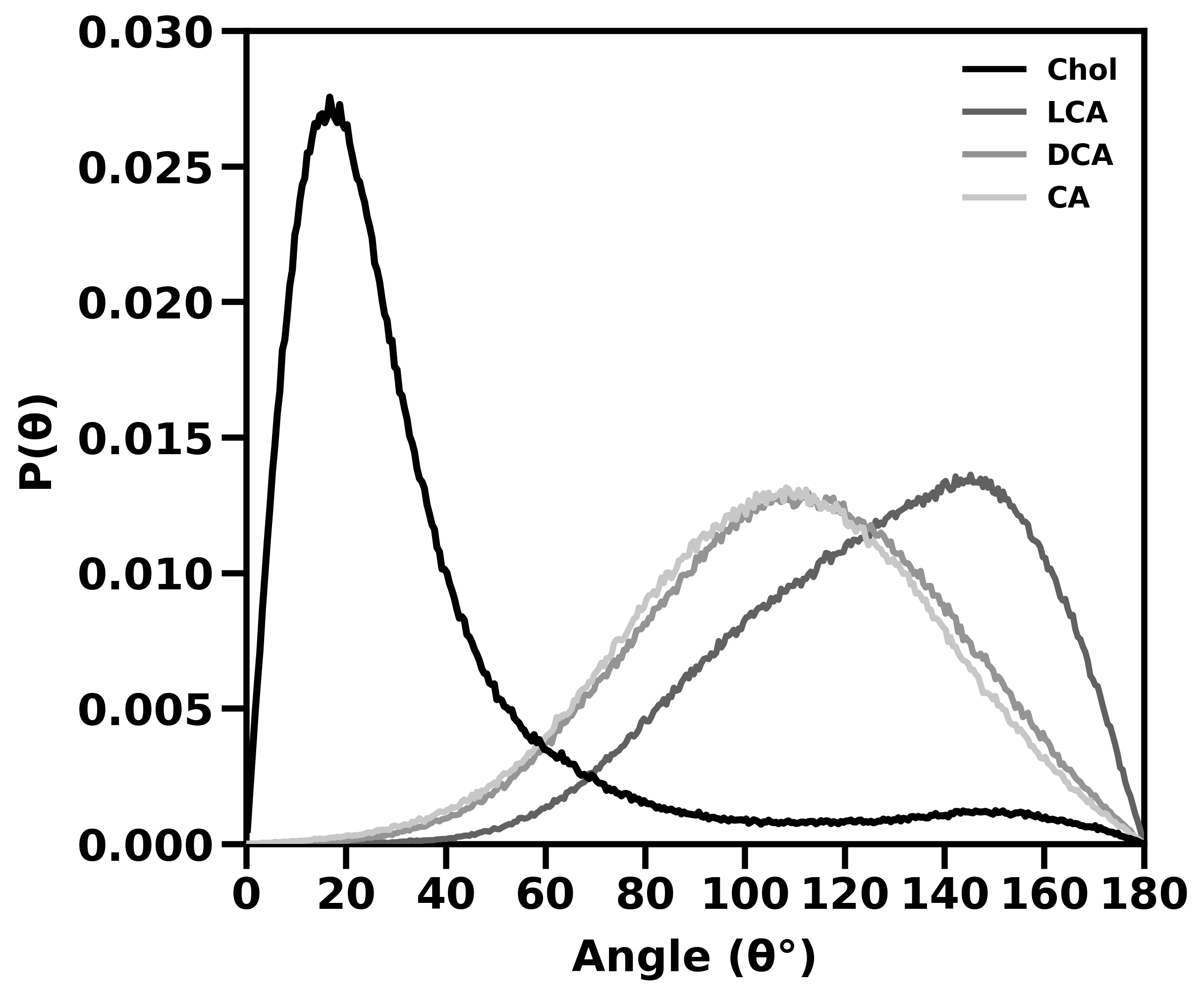** | **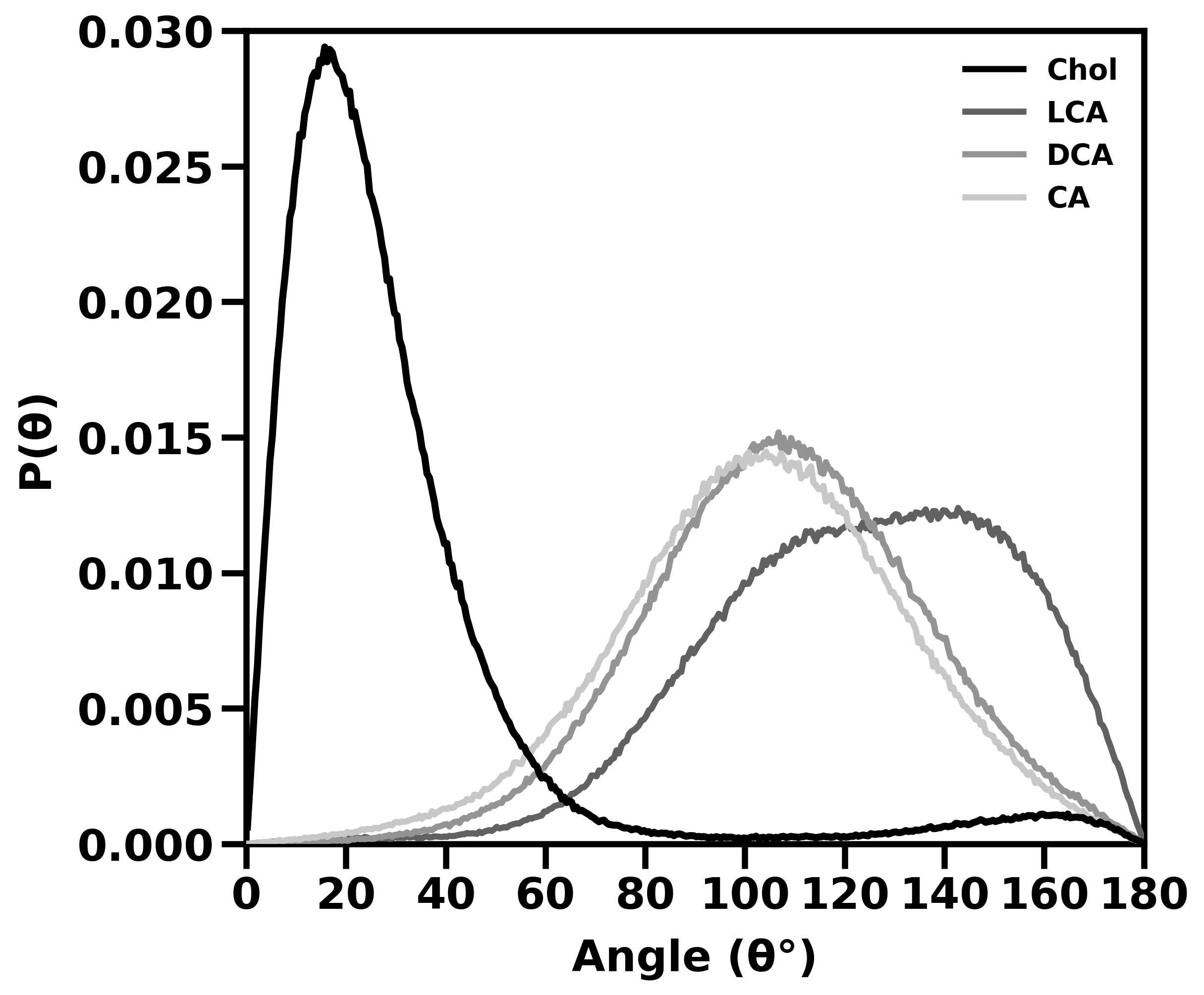** |
| **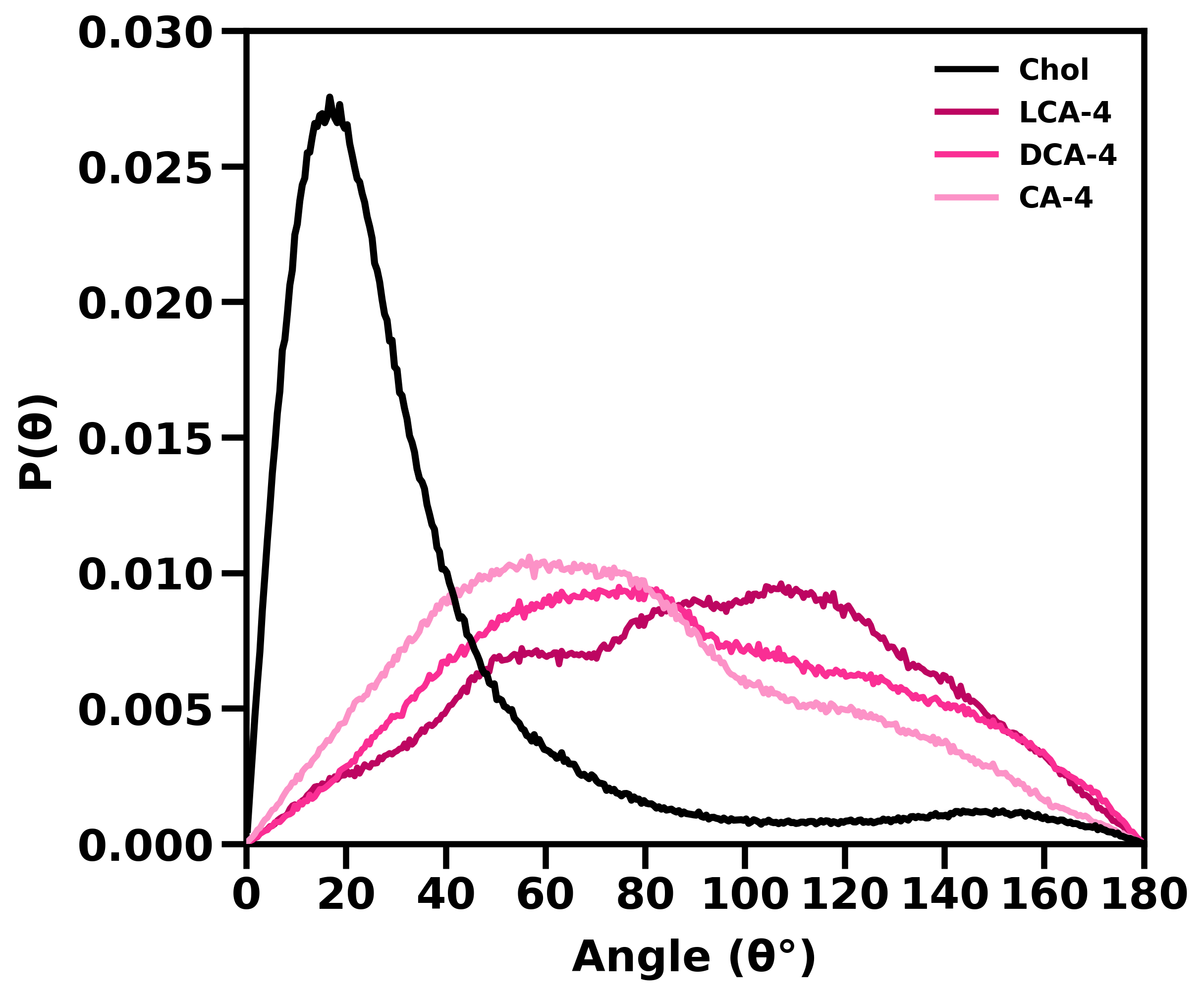** | **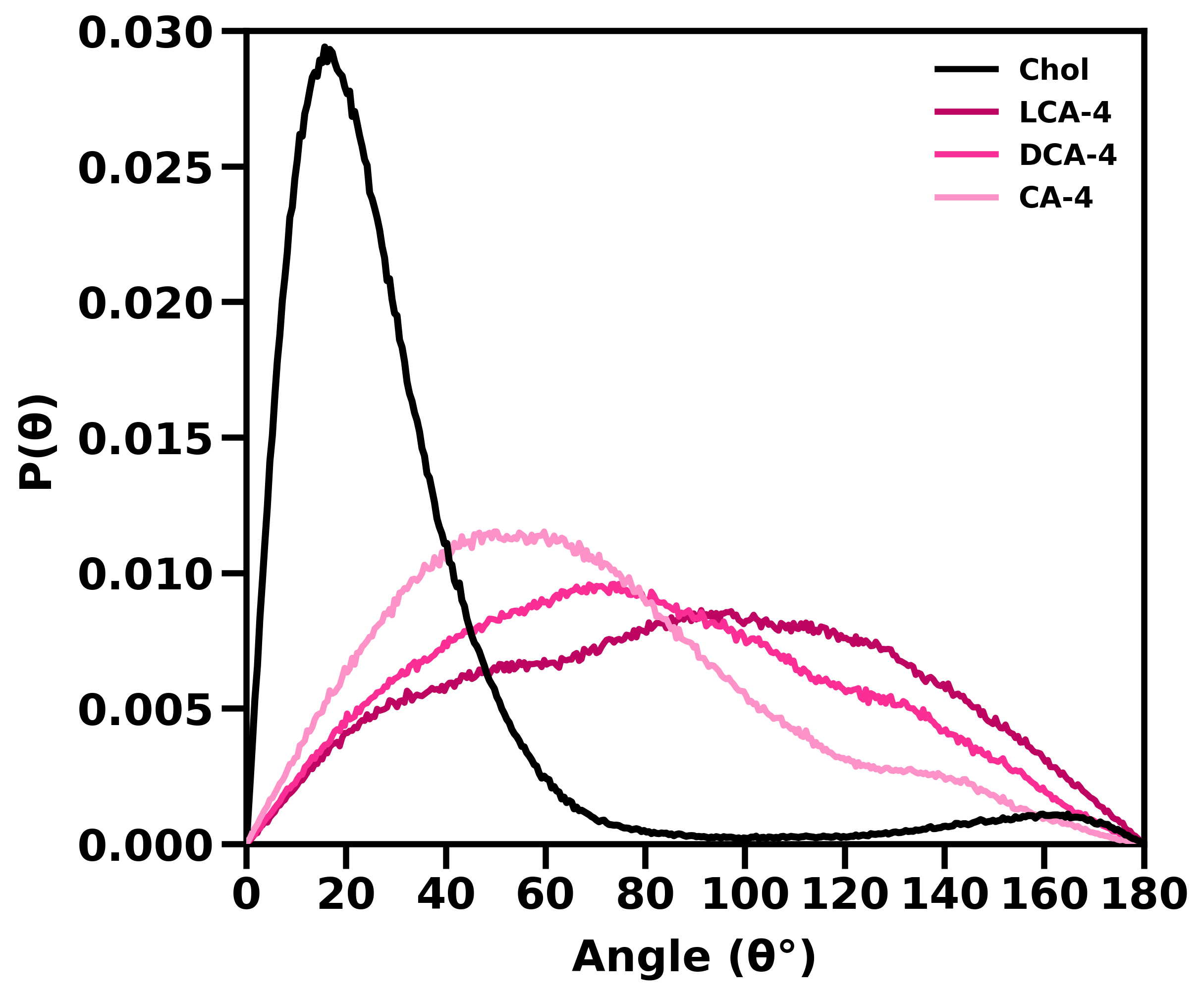** |
| **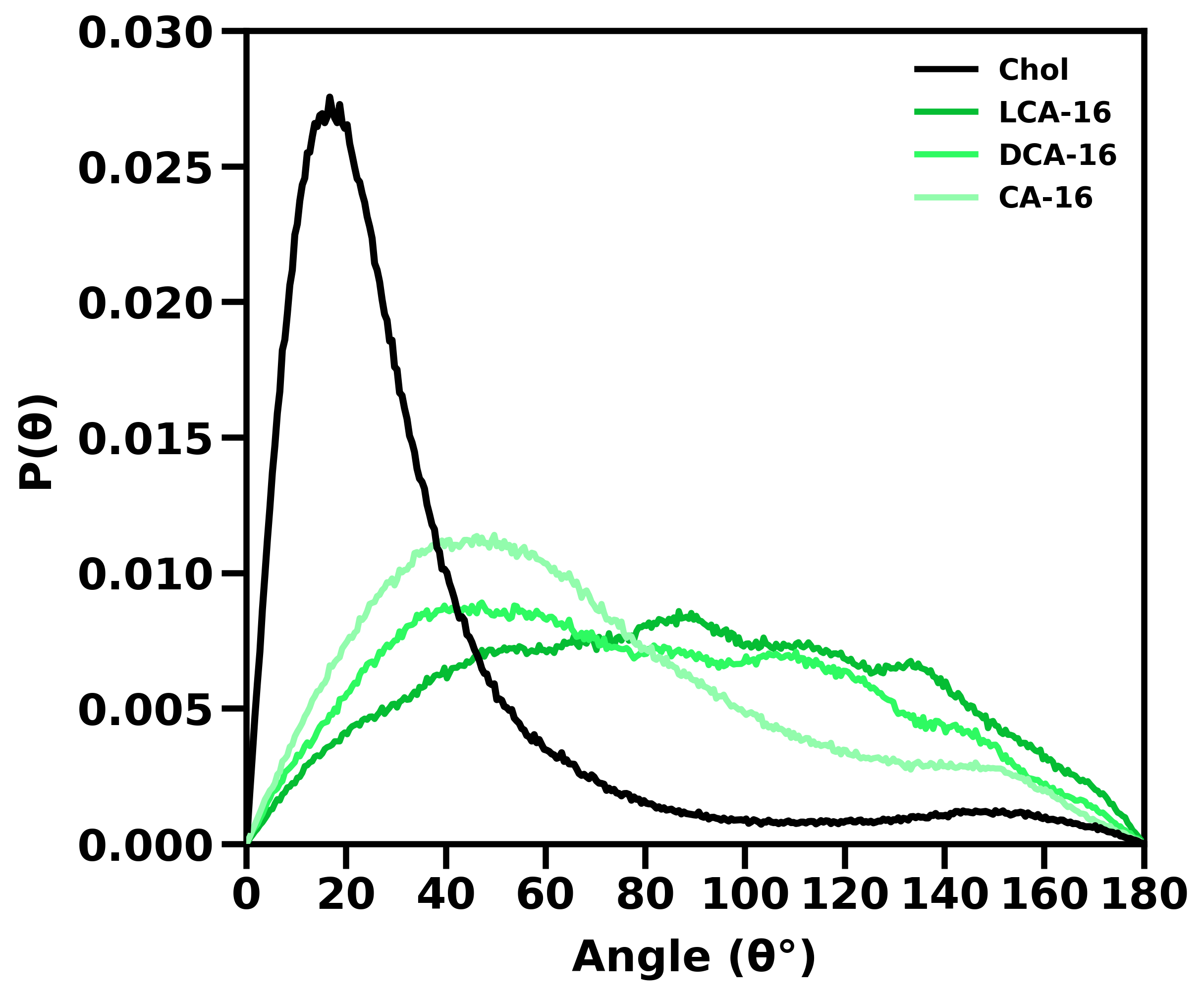** | **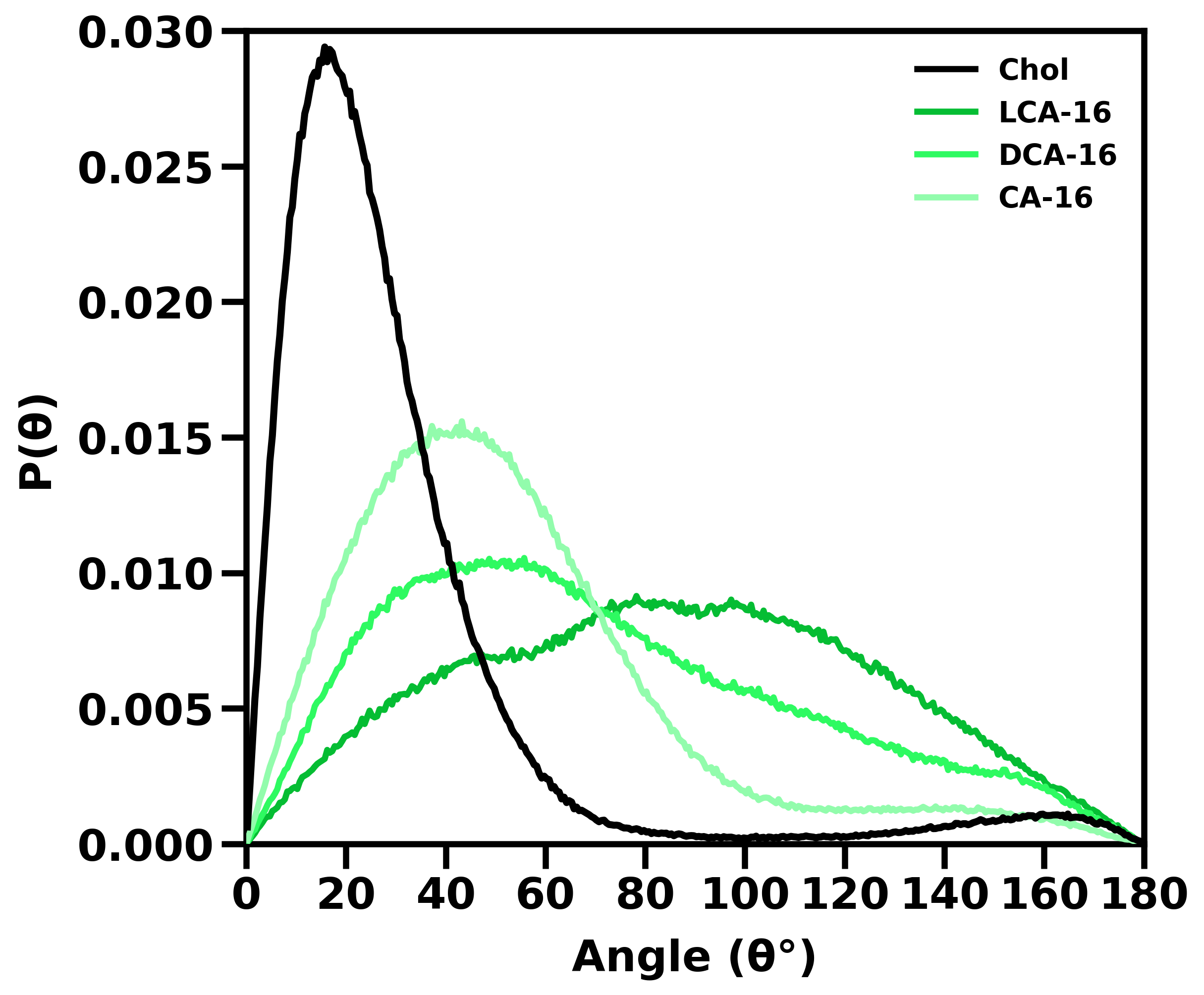** |
| **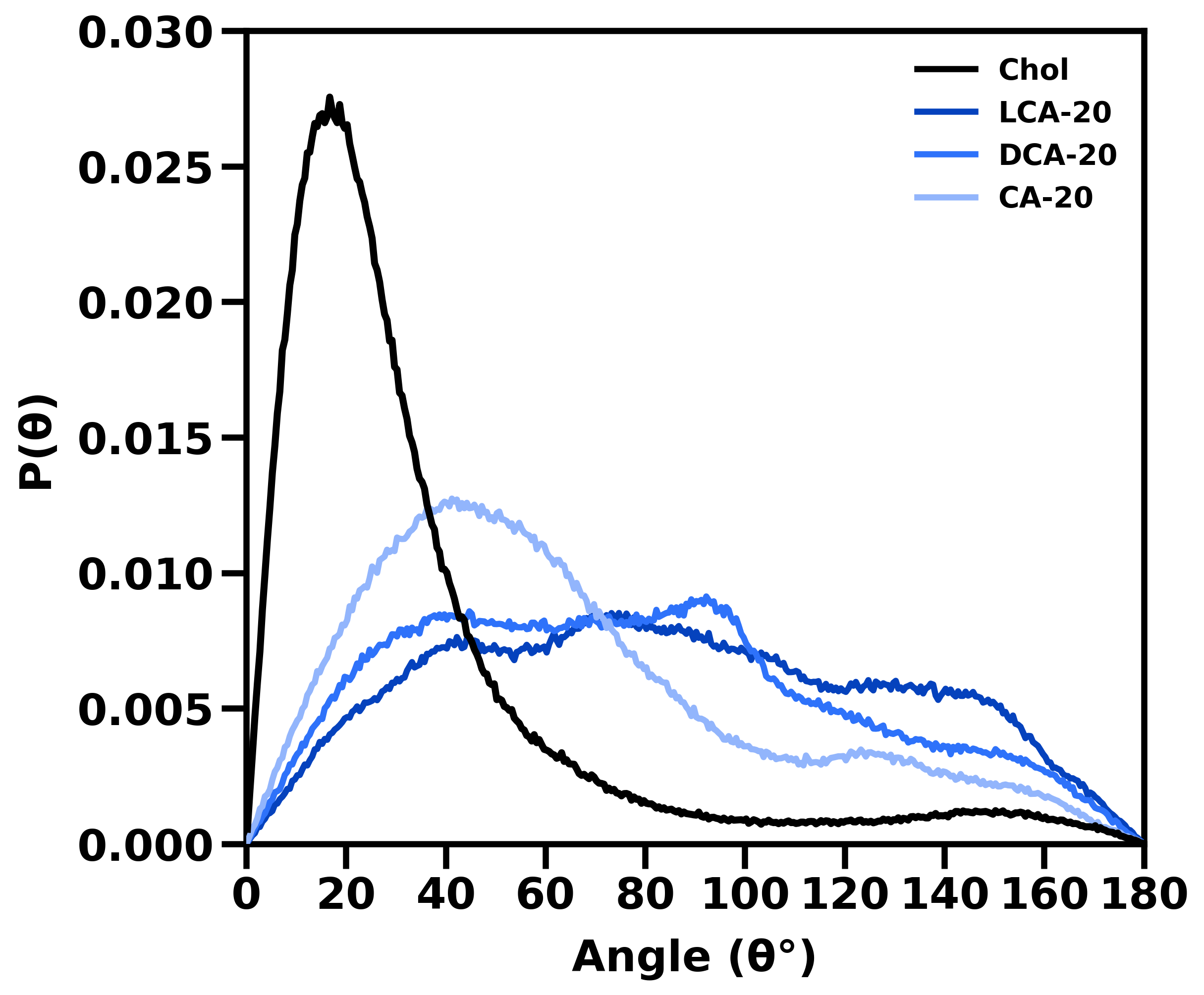** | **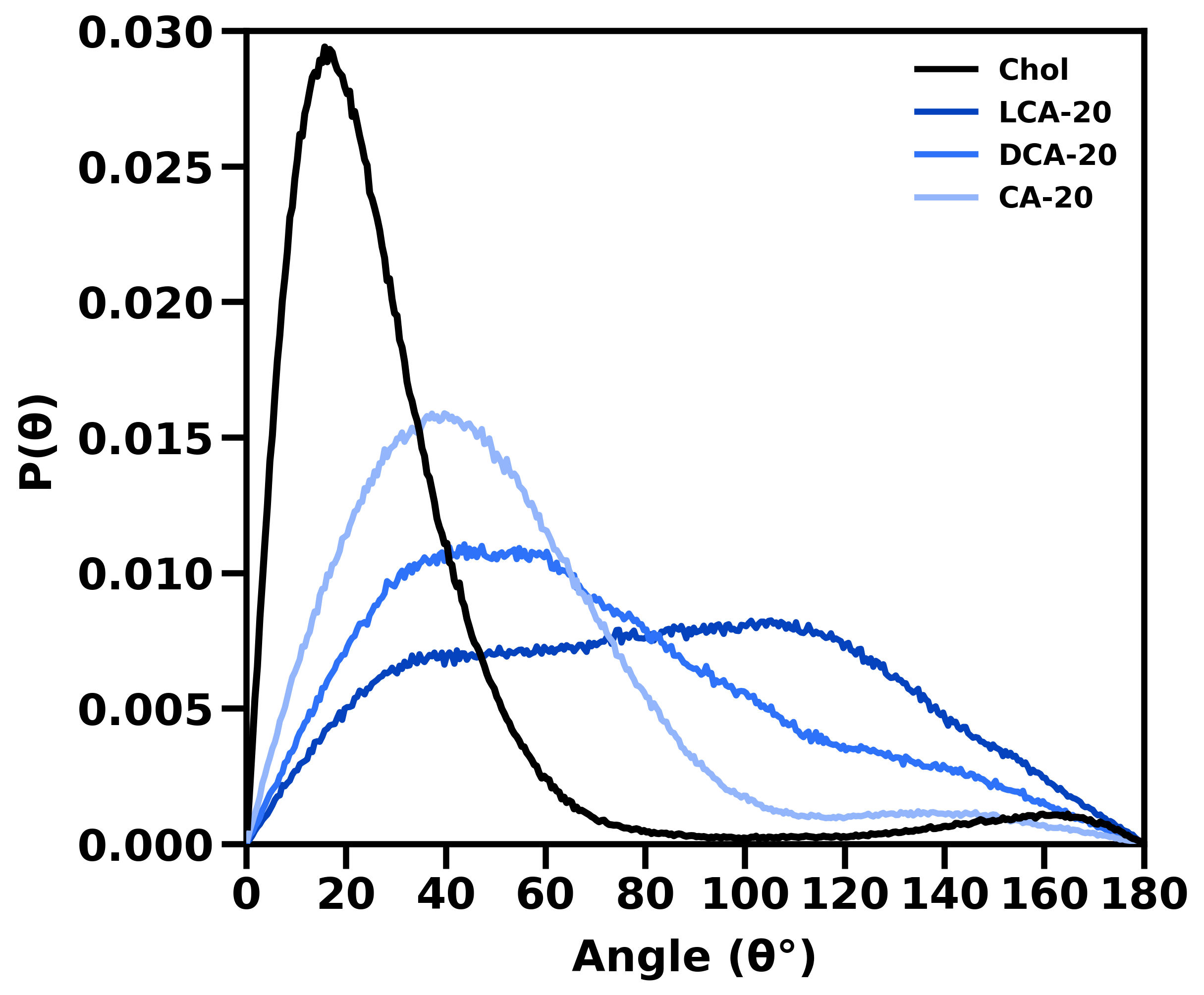** |

**Figure S15.** **Sterol tilt-angle distributions under neutral and protonated conditions**

Distributions of tilt angles (θ) for bile acid-derived sterol LNPs in neutral (A) and protonated (B) conditions. Each curve represents a distinct bile acid-derived sterol.

**Figure S16. Correlation of cryo-TEM-derived structural parameters with sterol tilt angle**

(A) Correlation between morphological irregularity quantified from cryo-TEM images and the mean sterol tilt angle obtained from MD simulations. (B) Correlation between membrane bilayer proportion quantified from cryo-TEM images and the mean sterol tilt angle. For each panel, a simple linear regression was performed and the best-fit line (solid) is shown with 95% confidence bands (dashed). R² values are indicated on each plot. Each point represents one LNP formulation. Point colors denote tail length series (Native (unmodified), C4, C16, and C20), and point shapes denote sterol scaffold (Chol, LCA, DCA, and CA), as indicated in the legend.


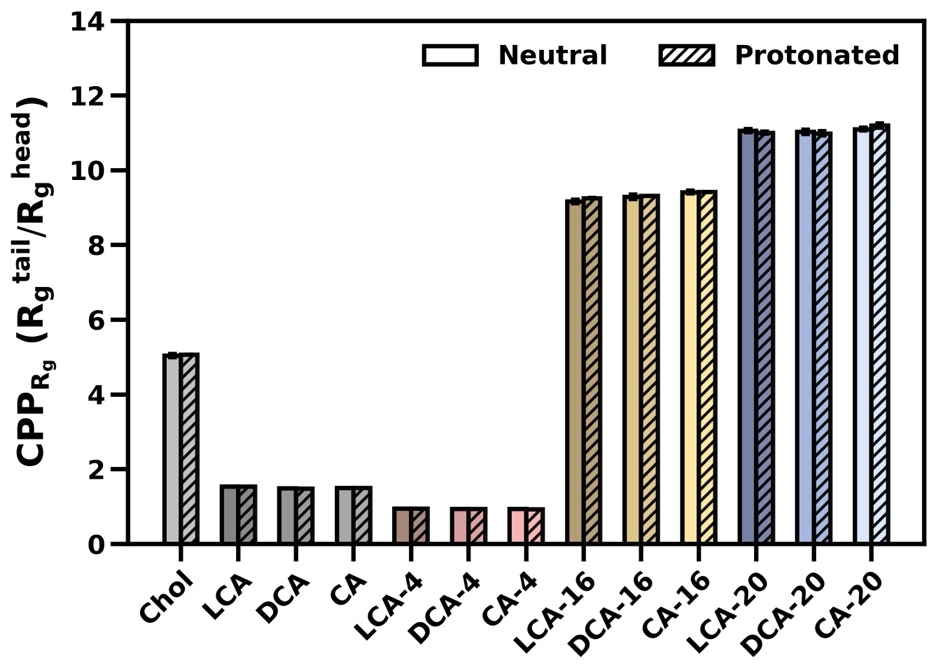


**Figure S17. CPP_Rg_ of sterols in LNP bilayers**

CPP_Rg_ of bile acid-derived sterols in the LNP bilayer systems under neutral and protonated conditions. Bars denote means across three replicates and error bars indicate SD.

**Figure S18. Comparison between the expanded linear predictor η and experimental physicochemical readouts**

Scatter plots comparing the expanded linear predictor η to the experimentally measured Z-average (A), polydispersity index (PDI) (B), and Zeta potential (C). Here, η is a standardized linear combination of CPP_Rg_, tilt angle, and N_OH_, with higher CPP_Rg_, lower tilt angle, and sterol-core hydroxylation contributing to the predictor. N_OH_ denotes the number of hydroxyl groups on the sterol core and was assigned as 1 for cholesterol and LCA-derived sterols, 2 for DCA-derived sterols, and 3 for CA-derived sterols. Each point represents one LNP formulation. Point colors denote tail length series (Native (unmodified), C4, C16, C20), and point shapes denote sterol scaffold (Chol, LCA, DCA, CA), as indicated in the legend.

Figure S19. Correlation of physicochemical properties with organ-level FLuc expression.

(A) Correlation plots between splenic FLuc expression (p/s, log10) and LNP physicochemical parameters (Z-average, zeta potential, and PDI). (B) Correlation plots between hepatic FLuc expression (p/s, log10) and the same physicochemical parameters (Z-average, zeta potential, and PDI). For each panel, a simple linear regression was performed and the best-fit line (solid) is shown with 95% confidence bands (dashed). R² values are indicated on each plot. Each point represents one LNP formulation. Point colors denote tail length series (Native (unmodified), C4, C16, C20) and point shapes denote sterol scaffold (Chol, LCA, DCA, CA), as indicated in the legend.

Figure S20. Serum hEPO levels and correlation with organ-level FLuc expression in the liver and spleen.

(A) Serum hEPO concentration measured by ELISA at 4 h after intravenous administration of hEPO-encoding mRNA-loaded LNPs (0.5 mg kg⁻¹) formulated with Chol, LCA-20, DCA-20, or CA-20 (female BALB/c mice, 6–7 weeks old, n = 3 biologically independent animals per group). (B–C) Correlation plots between serum hEPO concentration (mIU/mL, log₂) and organ-level FLuc expression (p/s, log₁₀) in the liver (B) and spleen (C). Simple linear regression is shown with the best-fit line (solid) and 95% confidence bands (dashed); R² values are indicated on each plot. Data are presented as individual data points with mean ± SD (A). Statistical analysis in (A) was performed using one-way ANOVA with Dunnett’s multiple comparisons test (****p < 0.0001), with all comparisons performed relative to the Chol LNP group.

**Figure S21. Antigen-specific immune responses induced by I.M. administration of Chol and CA-20 LNPs**

(A) Immunization schedule. BALB/c female mice (6–7 weeks old, n = 4 biologically independent animals per group) were intramuscularly administered DPBS, Chol LNPs, or CA-20 LNPs encapsulating HA-encoding mRNA at a dose of 0.5 mg kg⁻¹. Mice receiving mRNA-LNPs were primed and boosted 2 weeks later. Serum was collected on day 13 (post-prime) and day 28 (post-boost), and spleens were harvested on day 28. (B) Serum antibody titers (total IgG, IgG1, and IgG2a) measured by endpoint ELISA. Light-colored dots correspond to titers obtained after the prime immunization (day 13), whereas dark-colored dots correspond to titers obtained after the boost immunization (day 28). (C) IgG1/IgG2a ratio indicating the Th2/Th1 balance. (D) Hemagglutination inhibition (HI) titers against influenza A/Puerto Rico/8/1934 (H1N1), as a measure of antigen-specific neutralizing antibody responses. (E) Flow cytometry quantification of splenic plasma cells (CD138⁺ IgD⁻), GC B cells (GL7⁺ IgD⁻), and memory B cells (CD73⁺ IgD⁻) gated within CD3⁻ CD19⁺ B cells. (F–G) Antigen-specific cellular immune responses: IFN-γ-secreting splenocytes measured by ELISPOT following antigenic peptide stimulation (Sti) or non-stimulation (Non-Sti) (F), and cytokine levels (IFN-γ, IL-4, TNF-α) in splenocyte culture supernatants measured by ELISA under the same conditions (G). Data are presented as individual data points with mean ± SD. Statistical analyses were performed using one-way ANOVA followed by Fisher’s least significant difference (LSD) test (B–D), or one-way ANOVA with Tukey’s multiple comparisons test (E–G), as indicated (*p < 0.05, **p < 0.01, ***p < 0.001, ****p < 0.0001; ns, not significant).

Figure S22. Physicochemical stability of mRNA-loaded lipid nanoparticles (LNPs) formulated with cholesterol or CA-20 during storage at 4 °C.

To assess refrigerated storage stability, key physicochemical parameters were monitored at designated time points (0, 2, 4, 6, 9, and 16 days). (A) Encapsulation efficiency (EE%) (n = 3 independent measurements per time point). (B) Z-average (nm) (n = 5 independent measurements per time point). (C) Polydispersity index (PDI) (n = 5 independent measurements per time point). Data are presented as mean ± SD, with error bars indicating SD.

**Figure S23. Liver and spleen weight evaluation following I.V. of Chol and CA-20 LNPs**

(A) Experimental timeline for organ weight evaluation. Female ICR mice (6–7 weeks old, n = 5 biologically independent animals per group) were intravenously administered DPBS, Chol LNPs, or CA-20 LNPs encapsulating HA-encoding mRNA at a dose of 0.5 mg kg⁻¹. At 24 h post-injection, mice were euthanized, and liver and spleen tissues were collected for organ weight analysis. Representative images and quantitative analysis of (B) liver weight and (C) spleen weight normalized to body weight. Data are presented with individual data points and shown as box-and-whisker plots. Statistical significance was assessed using the Kruskal–Wallis test followed by Dunn’s multiple-comparisons test (*p < 0.05; ns, not significant).

**Figure S24. Acute safety evaluation of Chol and CA-20 LNPs following I.M. administration**

(A) Experimental timeline for toxicity evaluation. ICR female mice (6–7 weeks old, n = 5 biologically independent animals per group) were intramuscularly administered DPBS, Chol LNPs, or CA-20 LNPs encapsulating HA-encoding mRNA at a dose of 0.5 mg kg⁻¹. Body weight was measured 1 h before administration and 24 h post-administration. At 24 h post-injection, mice were euthanized, and blood and tissues were collected for serum biochemistry and histopathological analyses. (B) Body weight loss. Serum biochemical parameters including (C) alanine aminotransferase (ALT), (D) aspartate aminotransferase (AST), (E) lactate dehydrogenase (LDH), (F) creatine kinase (CK), (G) blood urea nitrogen (BUN), and (H) creatinine (Crea). (I) Representative H&E-stained liver sections (100× and 400× magnification). CV, central vein; PT, portal triad. Arrows indicate inflammatory cell infiltration, arrowheads indicate hepatocellular degeneration, and asterisks indicate sinusoidal dilatation. Semi-quantitative histopathological evaluation of liver sections including (J) inflammatory cell infiltration, (K) hepatocellular degeneration/vacuolation, (L) sinusoidal dilatation/congestion, and (M) necrosis. (N) Representative H&E-stained spleen sections (100× and 400× magnification). WP, white pulp; RP, red pulp; PALS, periarteriolar lymphoid sheath; GC, germinal center. Arrows indicate megakaryocytes, and arrowheads indicate vacuolation. Quantitative and semi-quantitative histopathological evaluation of spleen sections including (O) total tissue area, (P) white pulp area, (Q) megakaryocyte density, (R) red pulp expansion/disorganization, (S) foamy/vacuolated macrophage-like change, and (T) congestion. Data are presented with individual data points. Body weight and serum biochemistry data (B–H) are shown as box-and-whisker plots, whereas histopathological analyses (J–M, O–T) are presented as mean ± SD. Statistical significance was assessed using the Kruskal–Wallis test followed by Dunn’s multiple-comparisons test (*p < 0.05, **p < 0.01; ns, not significant).

**Figure S25. Liver and spleen weight evaluation following I.M. administration of Chol and CA-20 LNPs**

(A) Experimental timeline for organ weight evaluation. Female ICR mice (6–7 weeks old, n = 5 biologically independent animals per group) were intramuscularly administered DPBS, Chol LNPs, or CA-20 LNPs encapsulating HA-encoding mRNA at a dose of 0.5 mg kg⁻¹. At 24 h post-injection, mice were euthanized, and liver and spleen tissues were collected for organ weight analysis. Representative images and quantitative analysis of (B) liver weight and (C) spleen weight normalized to body weight. Data are presented with individual data points and shown as box-and-whisker plots. Statistical significance was assessed using the Kruskal–Wallis test followed by Dunn’s multiple-comparisons test (*p < 0.05; ns, not significant).

**Figure S26. Histopathological evaluation of skeletal muscle following I.M. administration of Chol and CA-20 LNPs**

(A) Representative H&E-stained skeletal muscle sections collected from the injection site at 24 h post-injection (100× and 400× magnification). MF, muscle fiber; Fas, fascicle; Epi, epimysium. Arrows indicate myofiber degeneration/necrosis, arrowheads indicate inflammatory cell infiltration, and asterisks indicate edema. Semi-quantitative histopathological evaluation of skeletal muscle sections including (B) myofiber degeneration, (C) myofiber necrosis, (D) interstitial edema, and (E) inflammatory cell infiltration. Data are presented as individual data points with mean ± SD. Statistical significance was assessed using the Kruskal–Wallis test followed by Dunn’s multiple-comparisons test (*p < 0.05, **p < 0.01; ns, not significant).


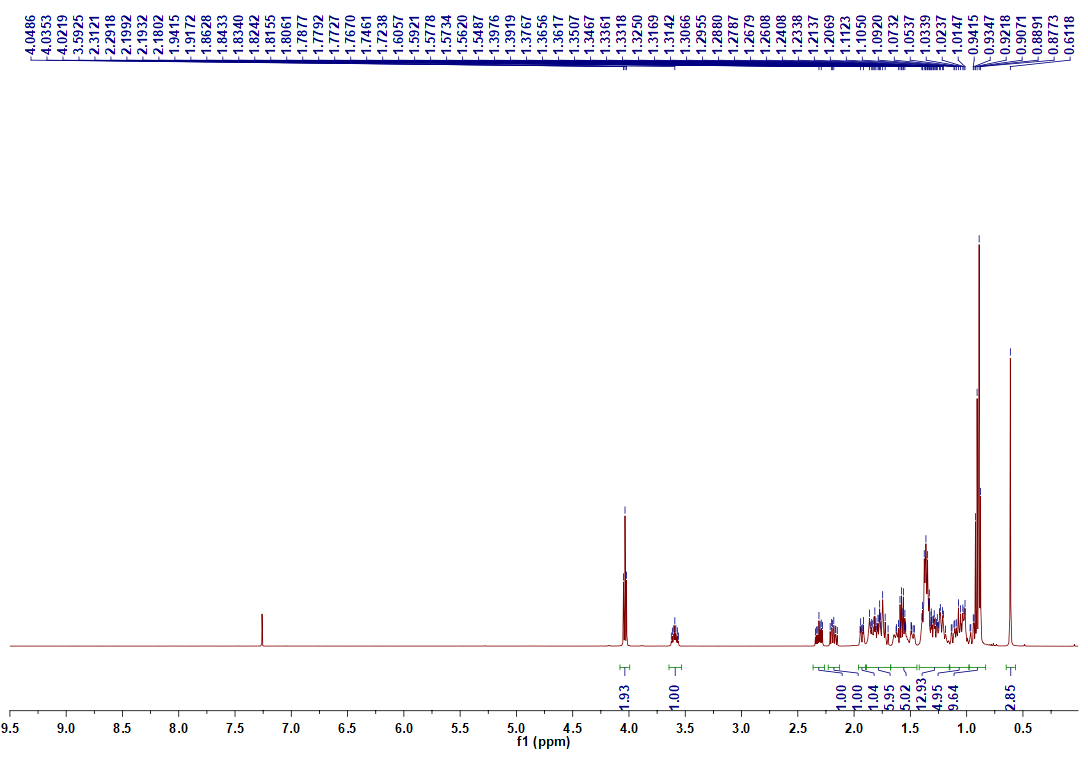


**Figure S27.** ^1^H NMR spectrum of LCA-4


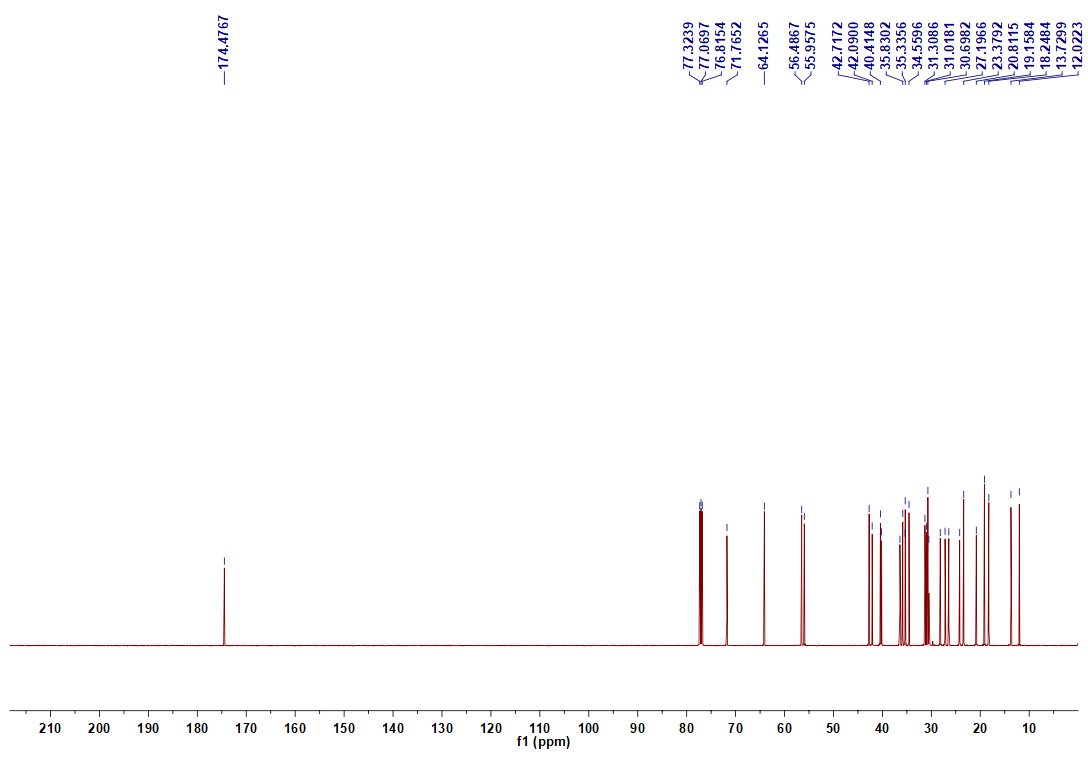


**Figure S28.** ^13^C NMR spectrum of LCA-4

**
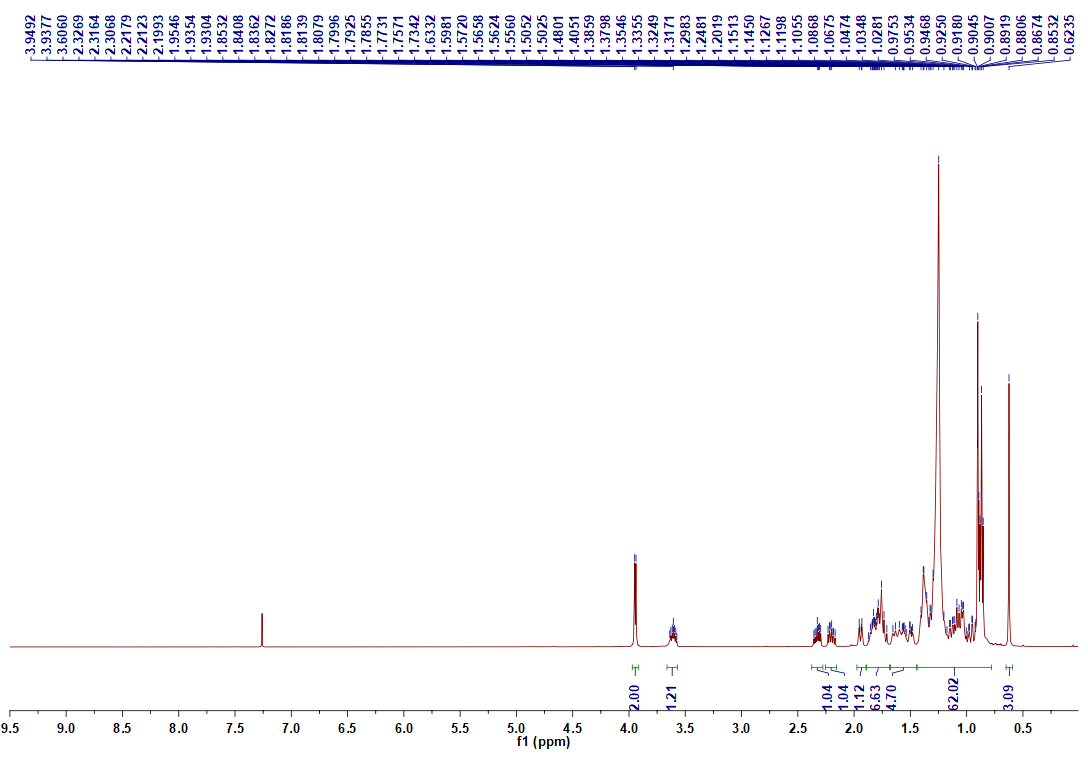
**

**Figure S29.** ^1^H NMR spectrum of LCA-16


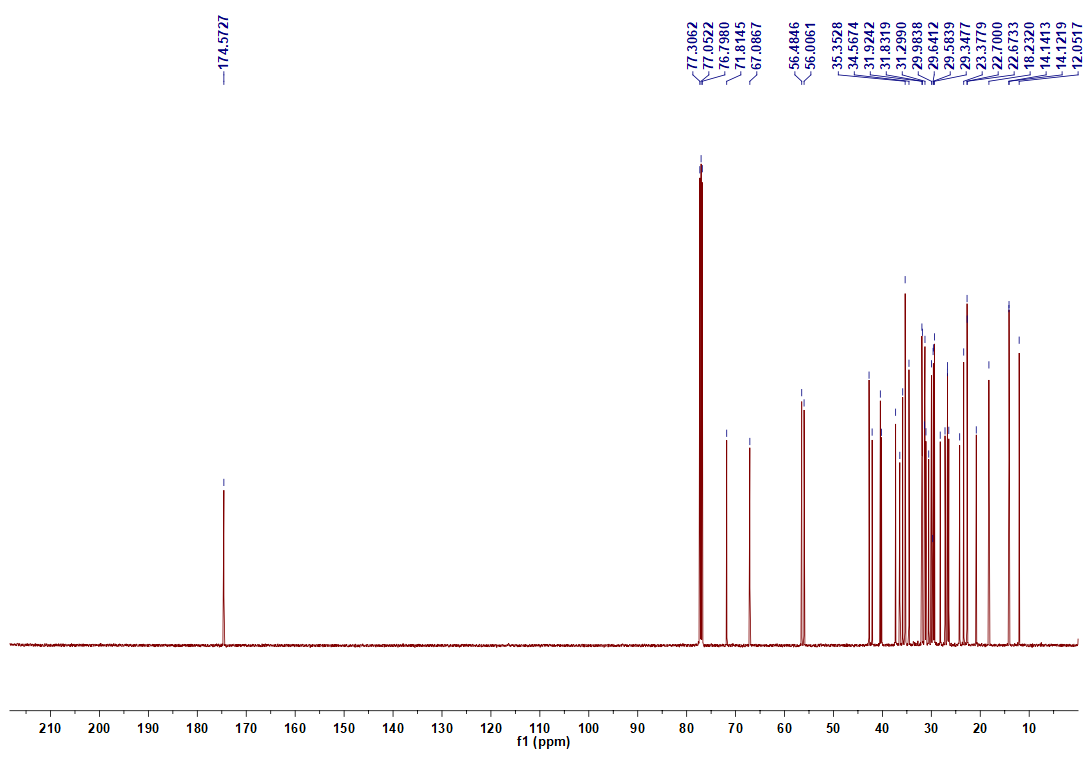


**Figure S30.** ^13^C NMR spectrum of LCA-16


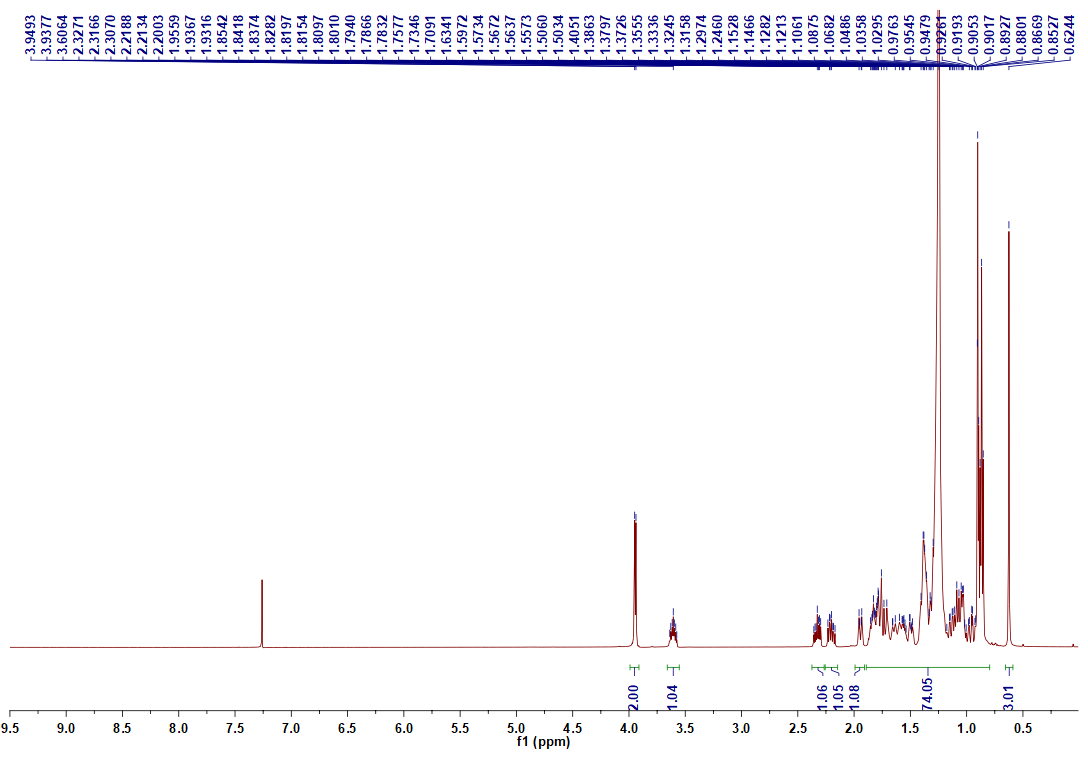


**Figure S31.** ^1^H NMR spectrum of LCA-20


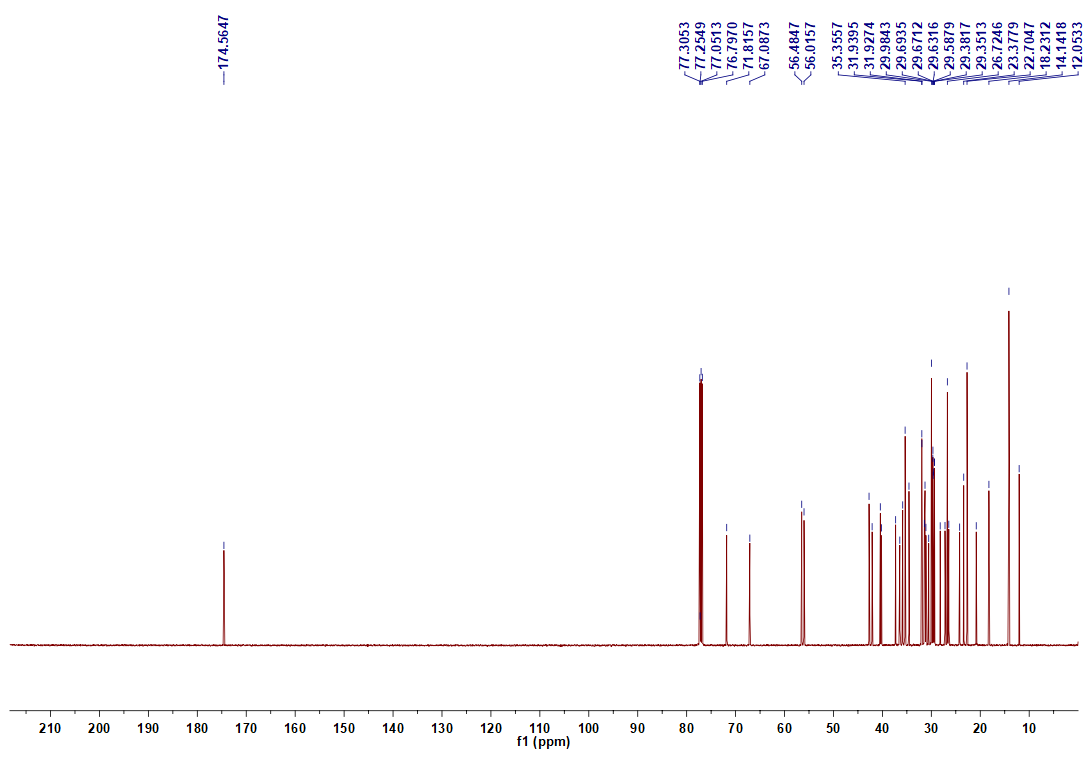


**Figure S32.** ^13^C NMR spectrum of LCA-20


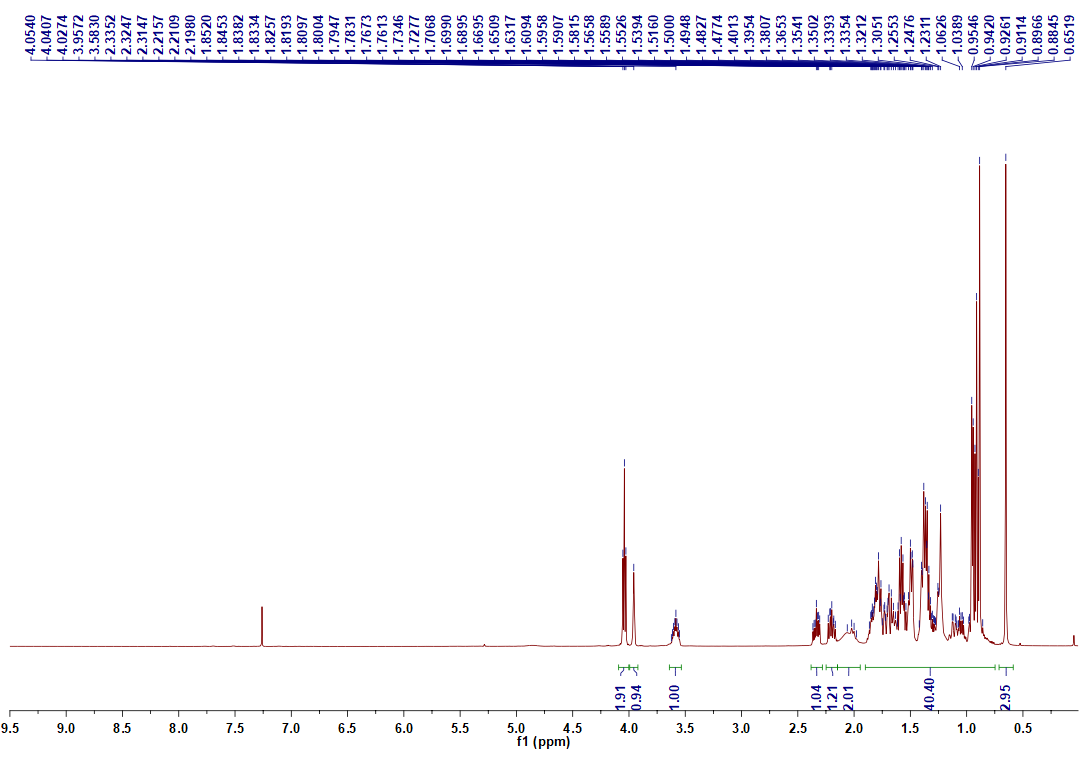


**Figure S33.** ^1^H NMR spectrum of DCA-4


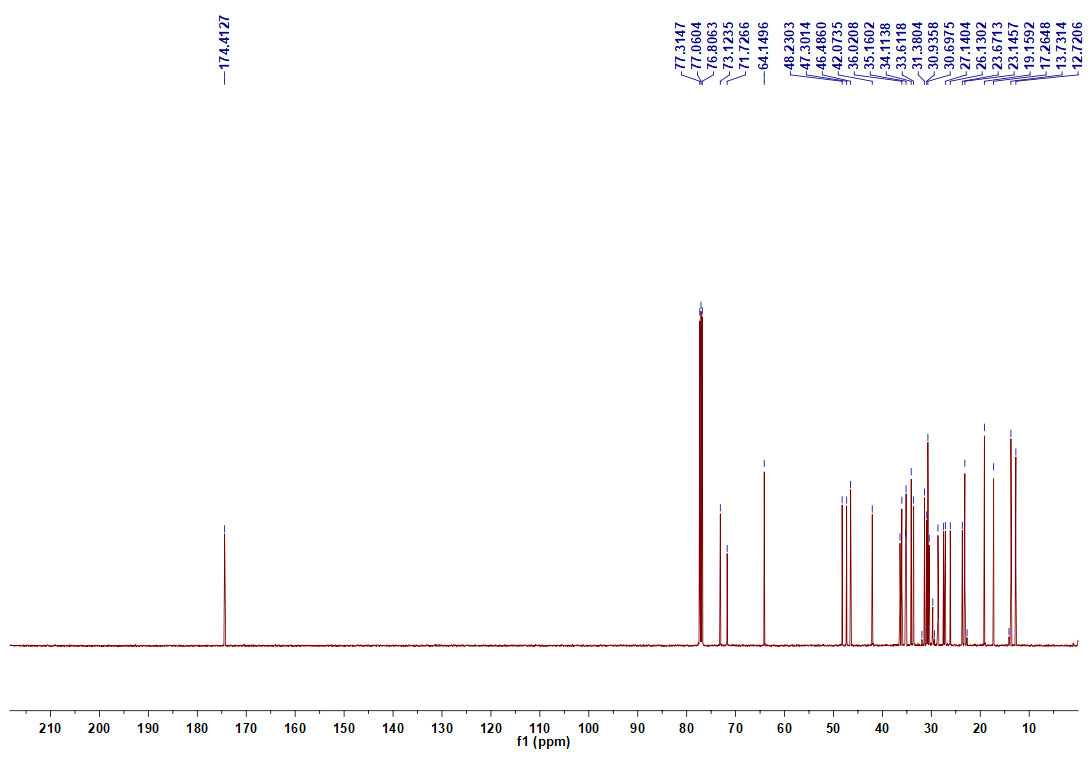


**Figure S34.** ^13^C NMR spectrum of DCA-4


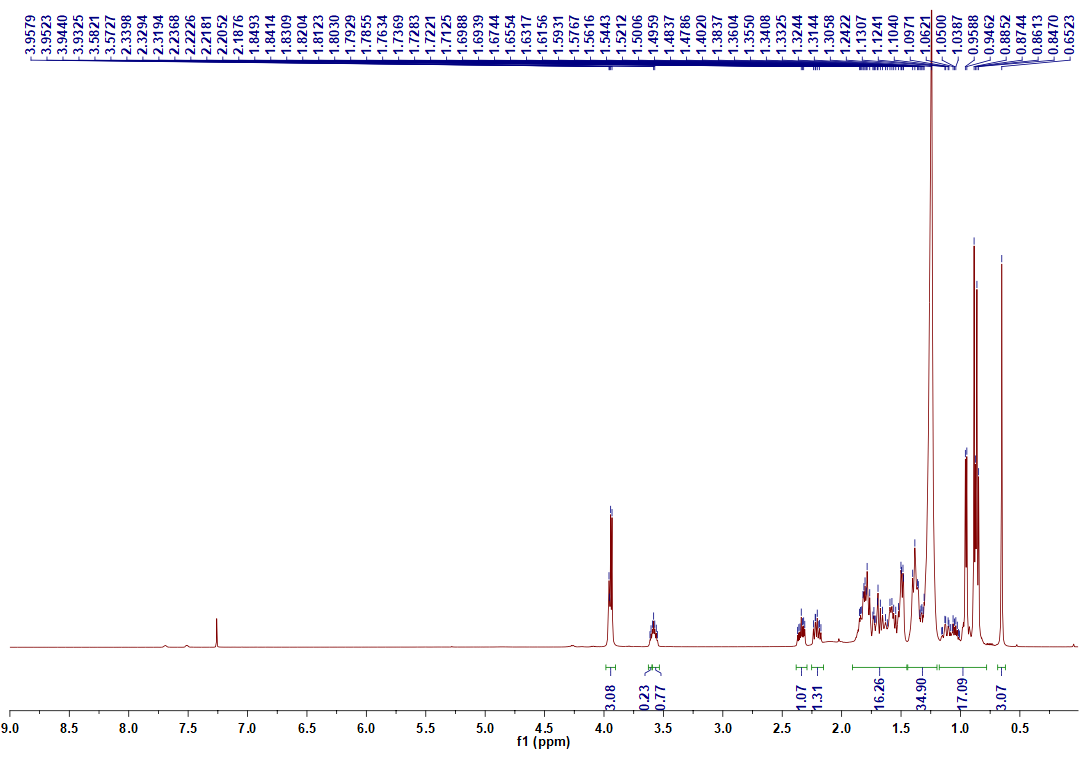


**Figure S35.** ^1^H NMR spectrum of DCA-16


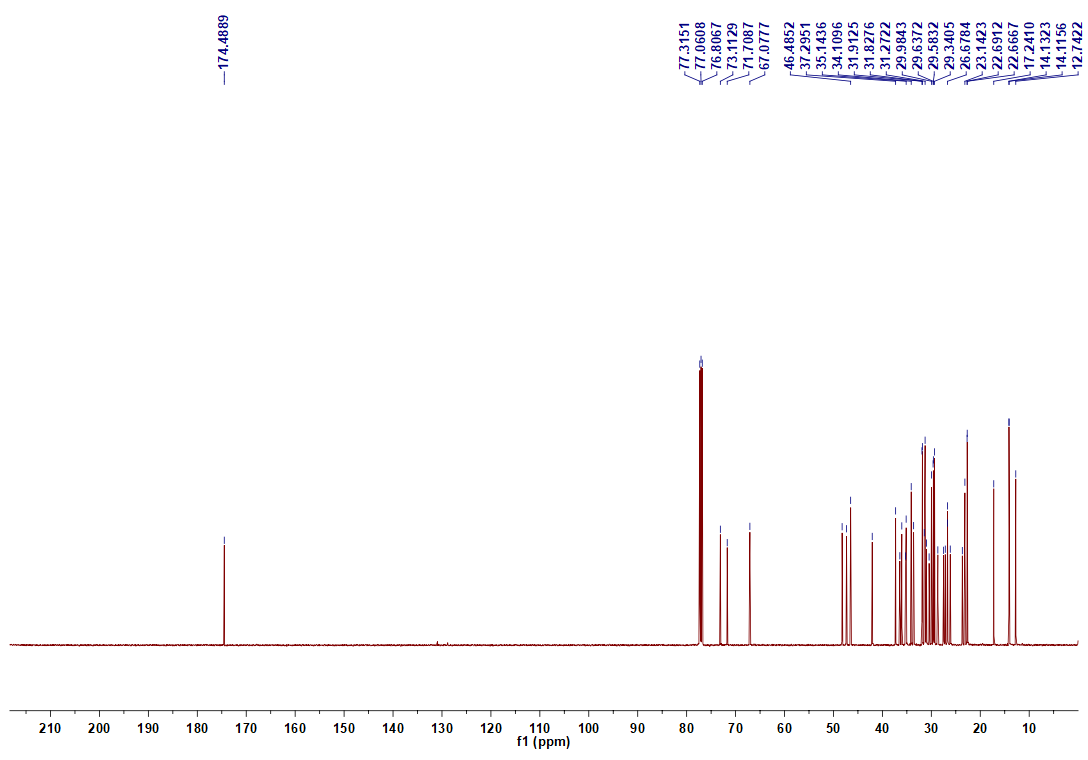


**Figure S36.** ^13^C NMR spectrum of DCA-16


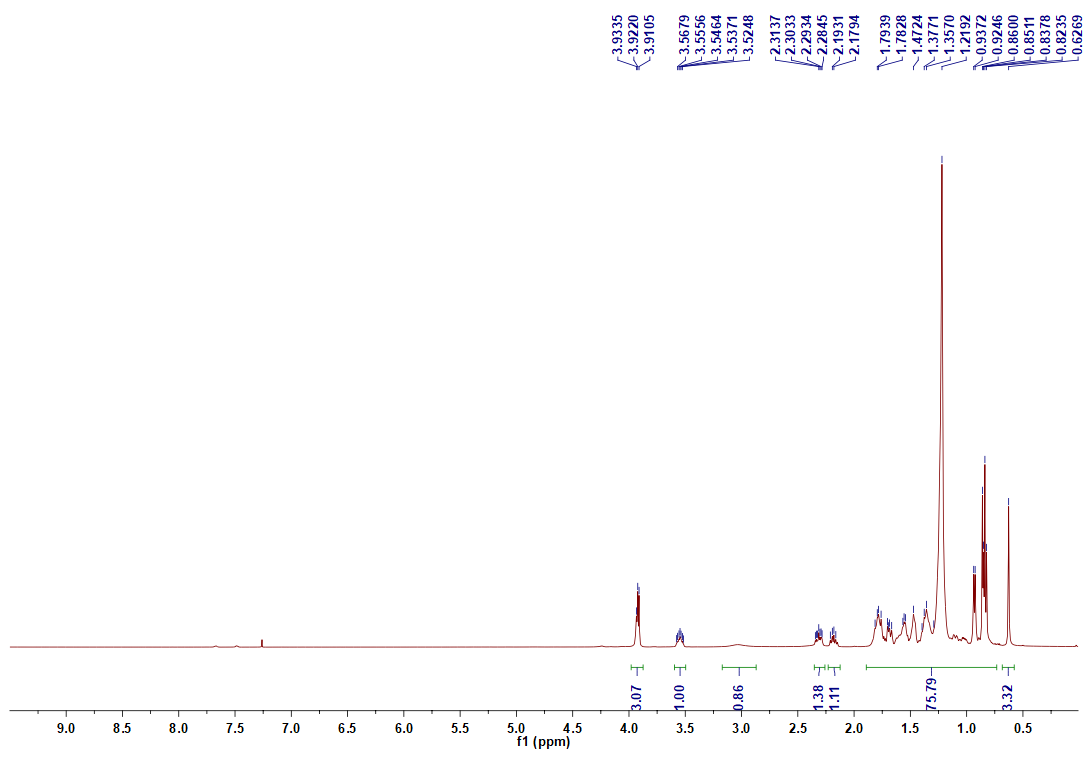


**Figure S37.** ^1^H NMR spectrum of DCA-20


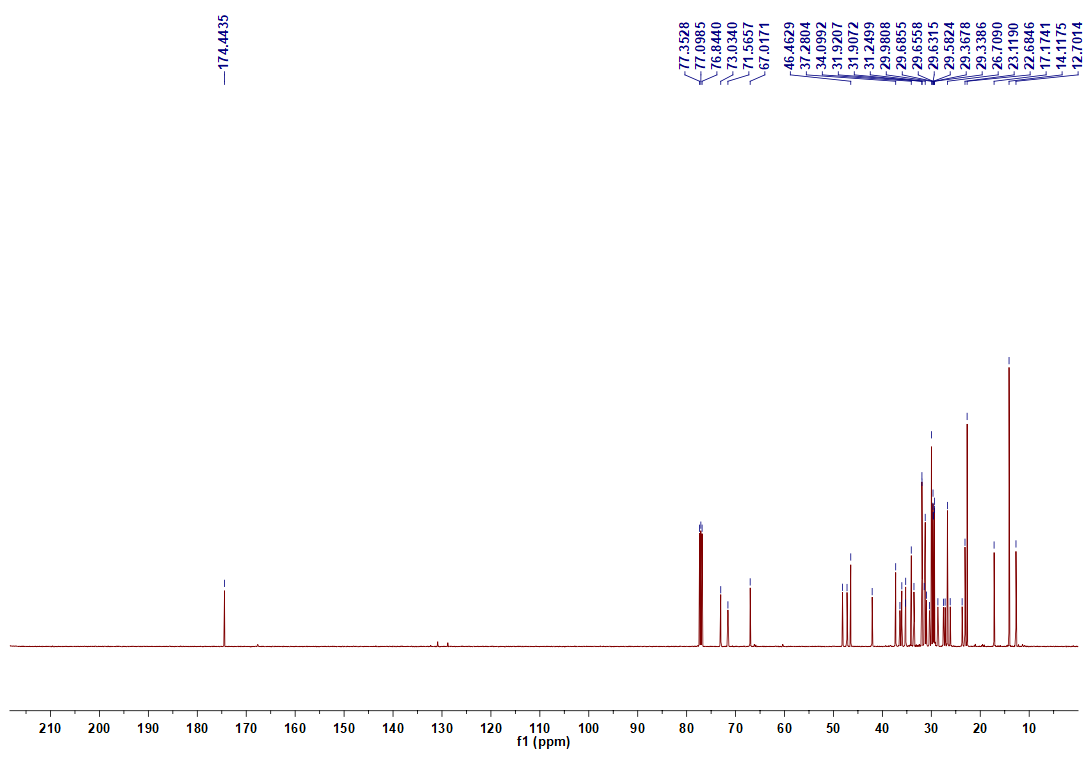


**Figure S38.** ^13^C NMR spectrum of DCA-20


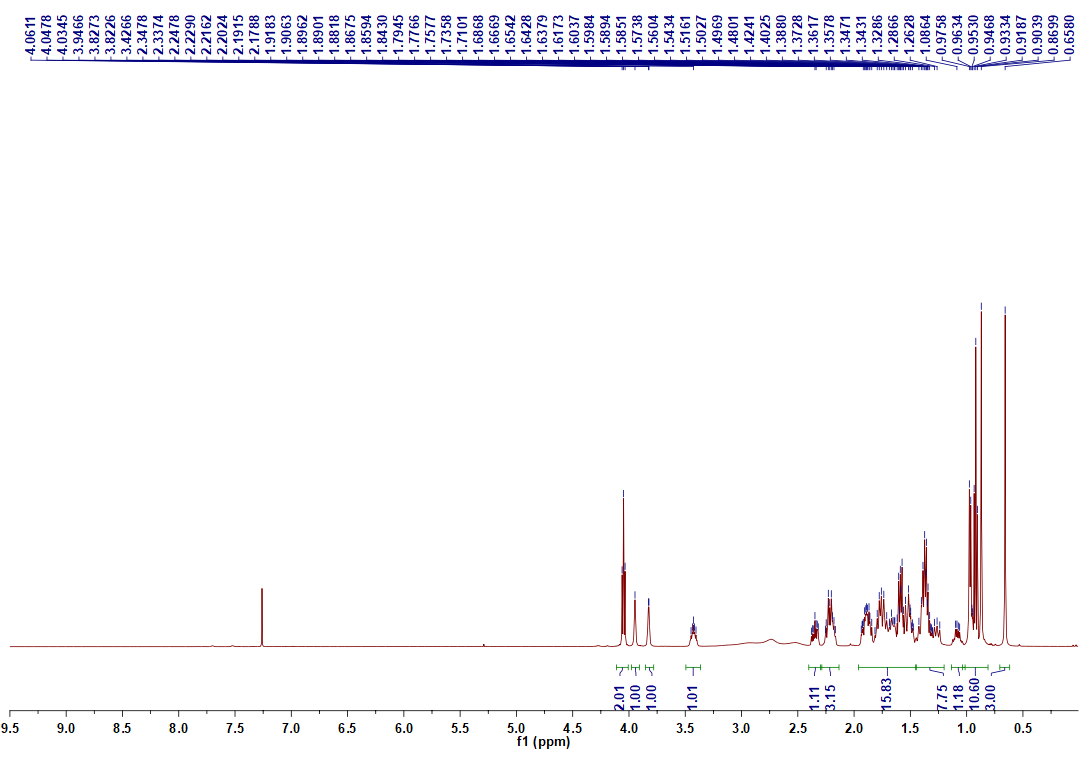


**Figure S39.** ^1^H NMR spectrum of CA-4


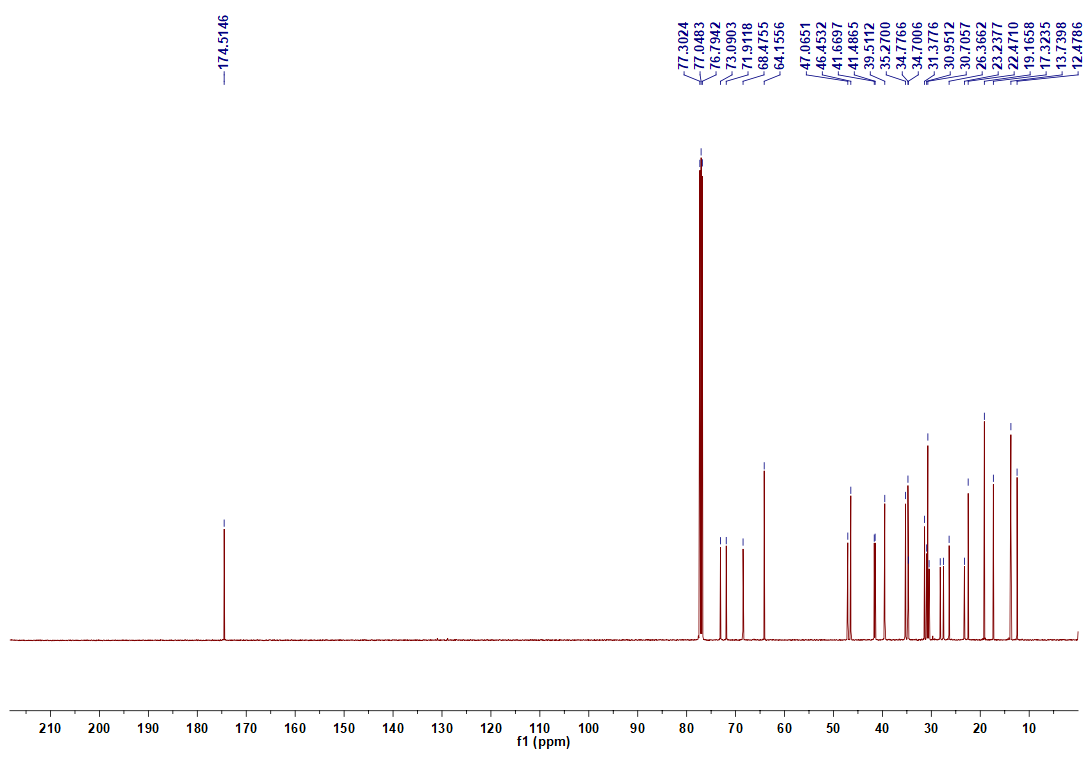


**Figure S40.** ^13^C NMR spectrum of CA-4


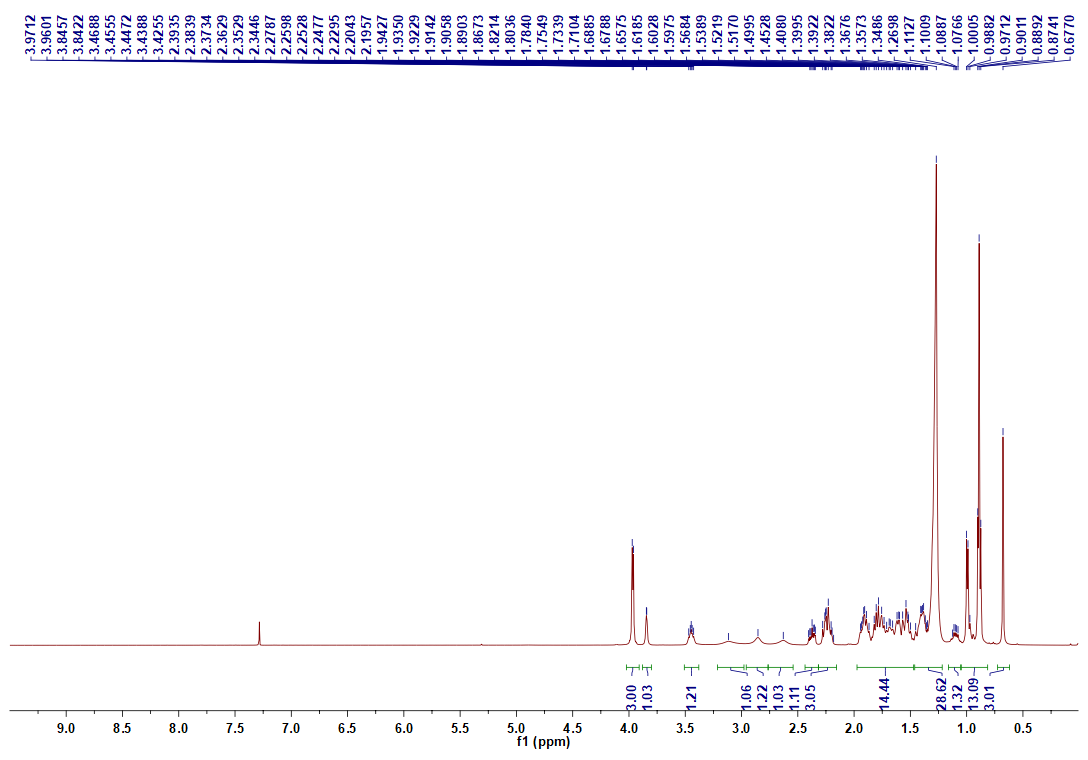


**Figure S41.** ^1^H NMR spectrum of CA-16


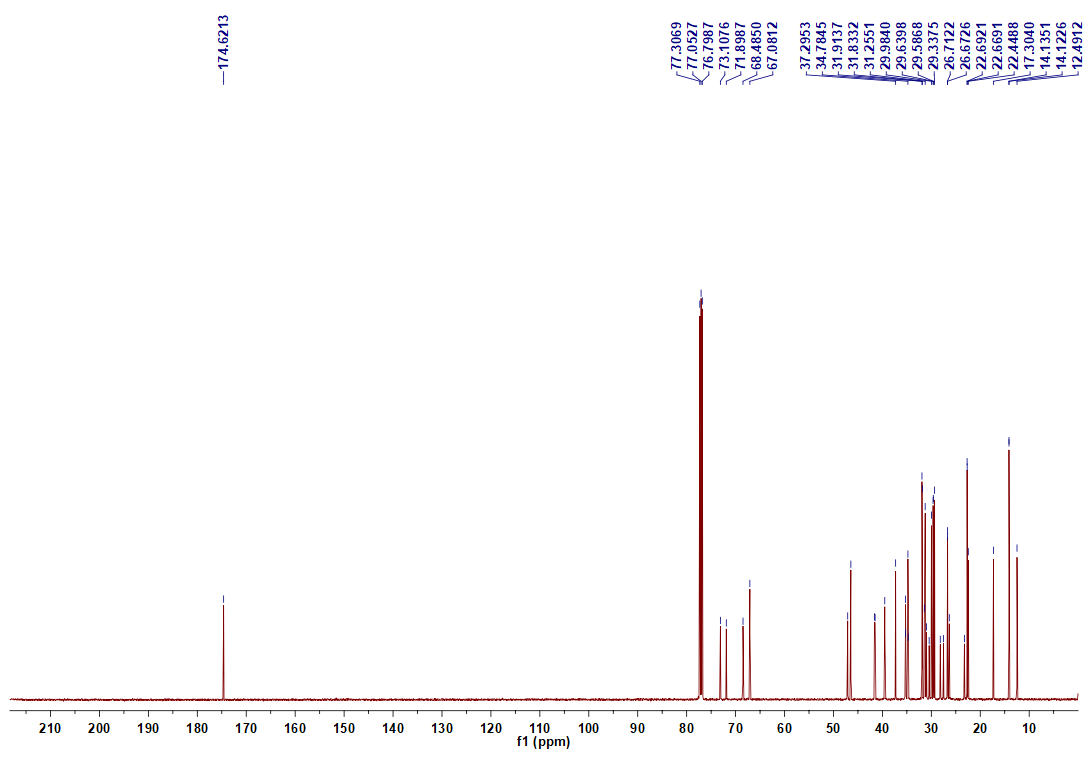


**Figure S42.** ^13^C NMR spectrum of CA-16


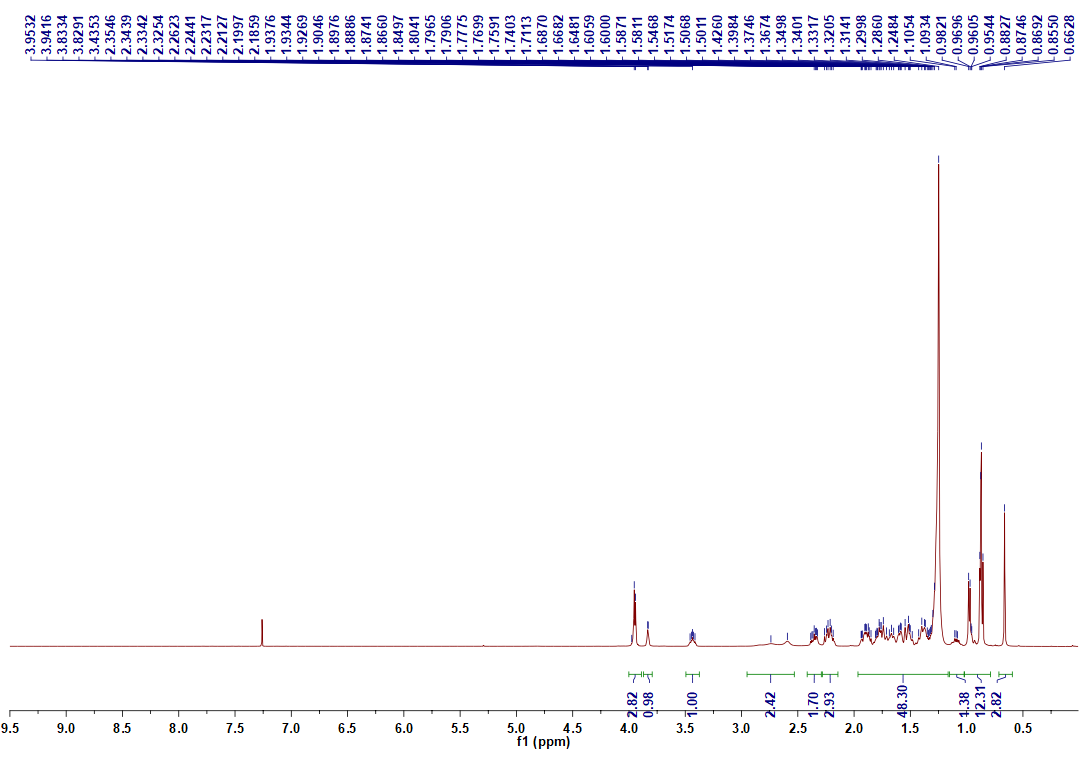


**Figure S43.** ^1^H NMR spectrum of CA-20


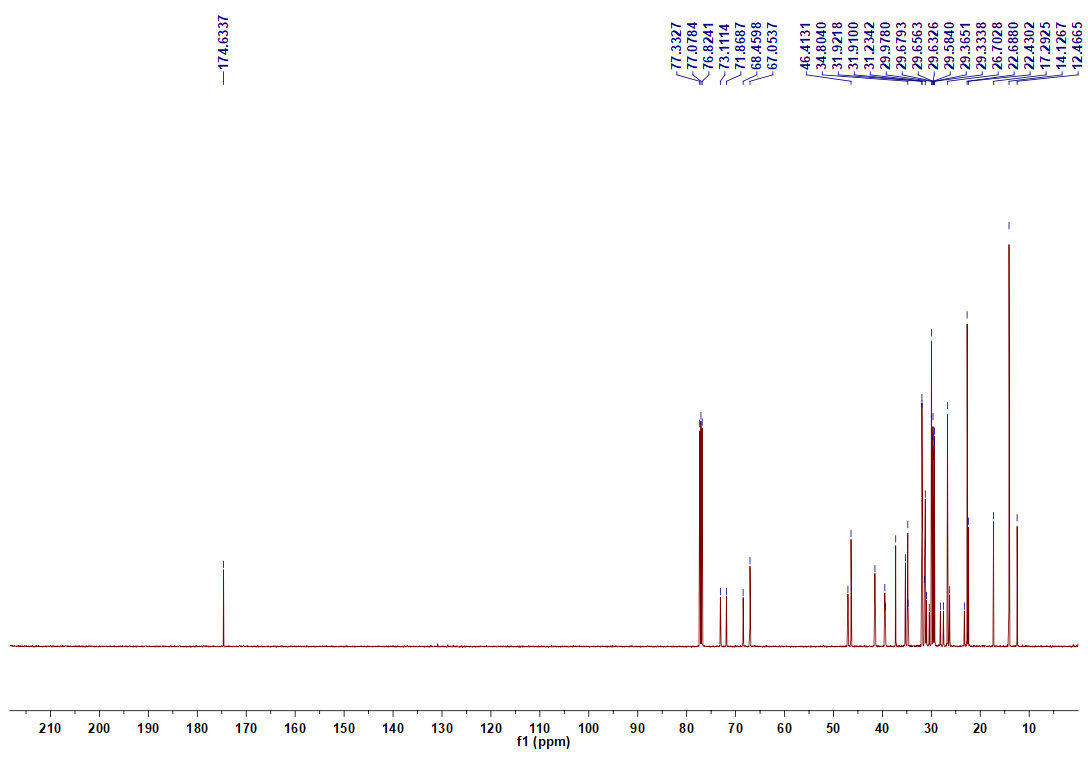


**Figure S44.** ^13^C NMR spectrum of CA-20


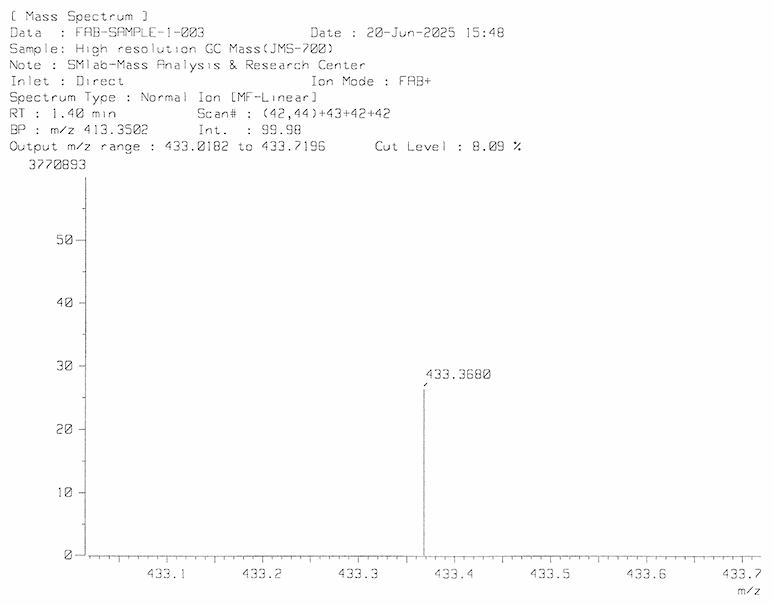


**Figure S45.** High resolution mass spectrum (HRMS) of LCA-4.


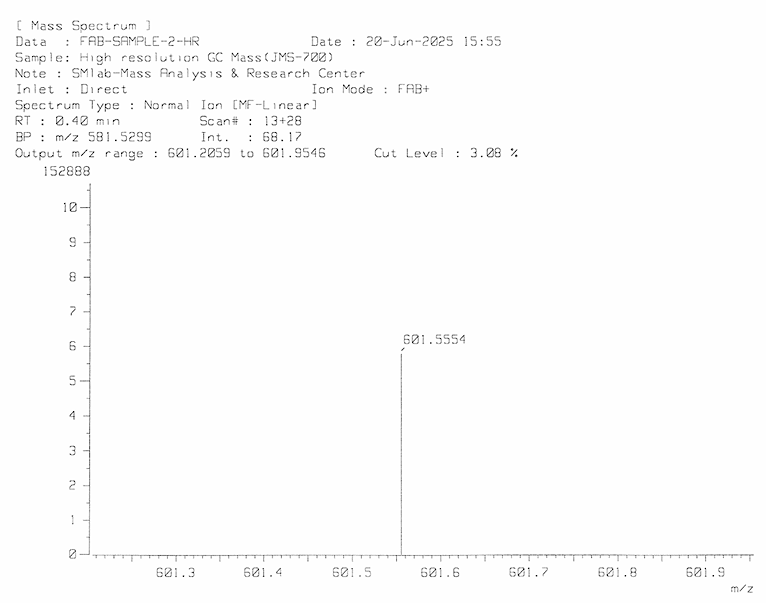


**Figure S46.** High resolution mass spectrum (HRMS) of LCA-16.

**
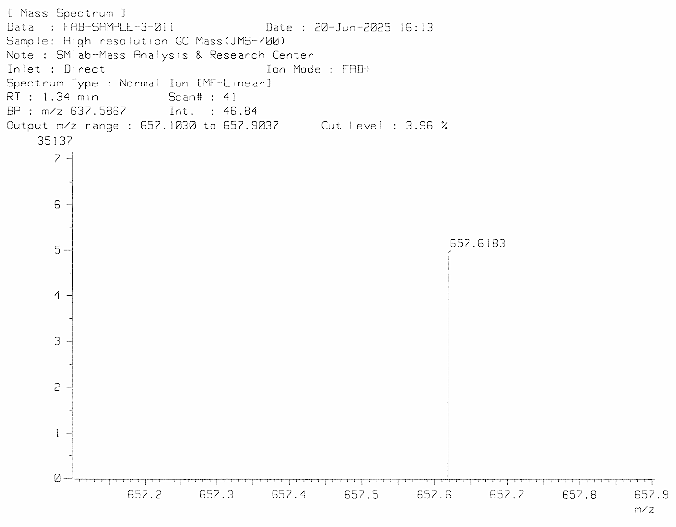
**

**Figure S47.** High resolution mass spectrum (HRMS) of LCA-20.

**
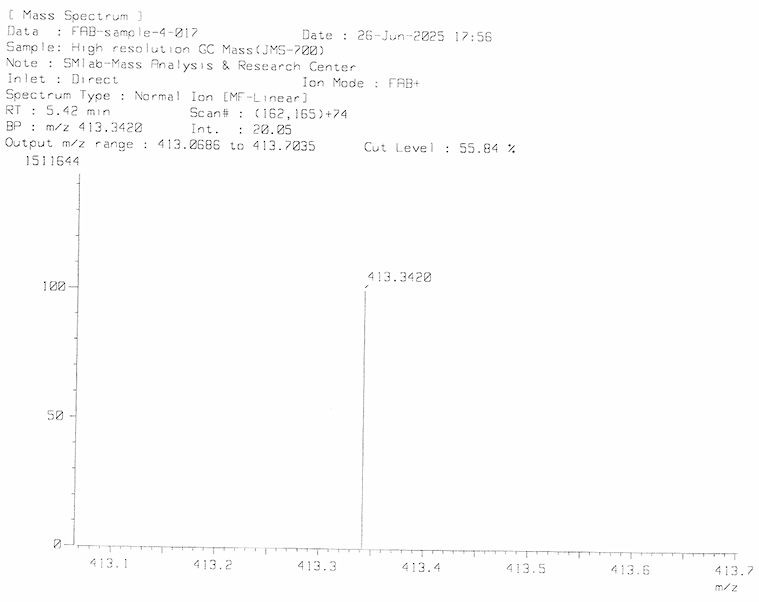
**

**Figure S48.** High resolution mass spectrum (HRMS) of DCA-4.


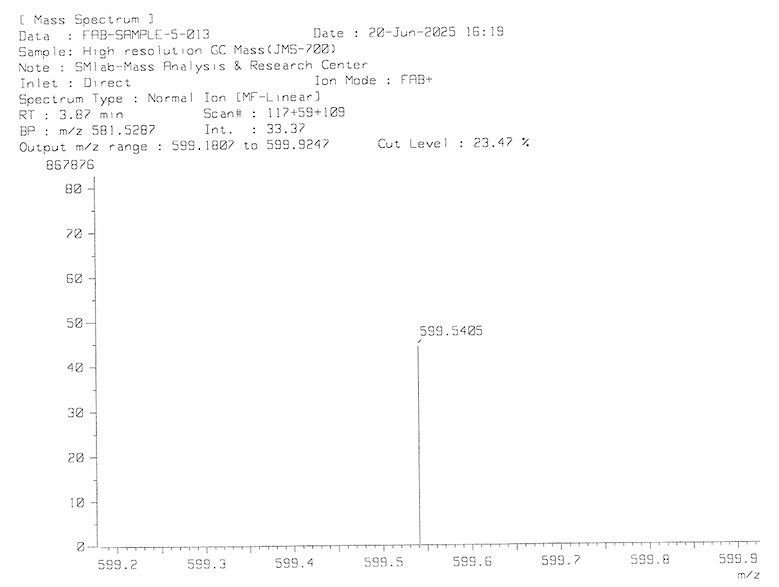


**Figure S49.** High resolution mass spectrum (HRMS) of DCA-16.


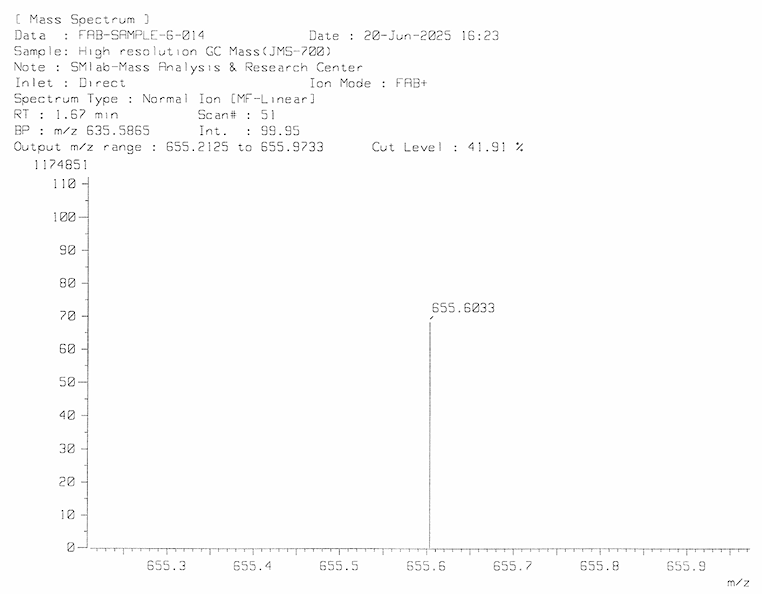


**Figure S50.** High resolution mass spectrum (HRMS) of DCA-20.


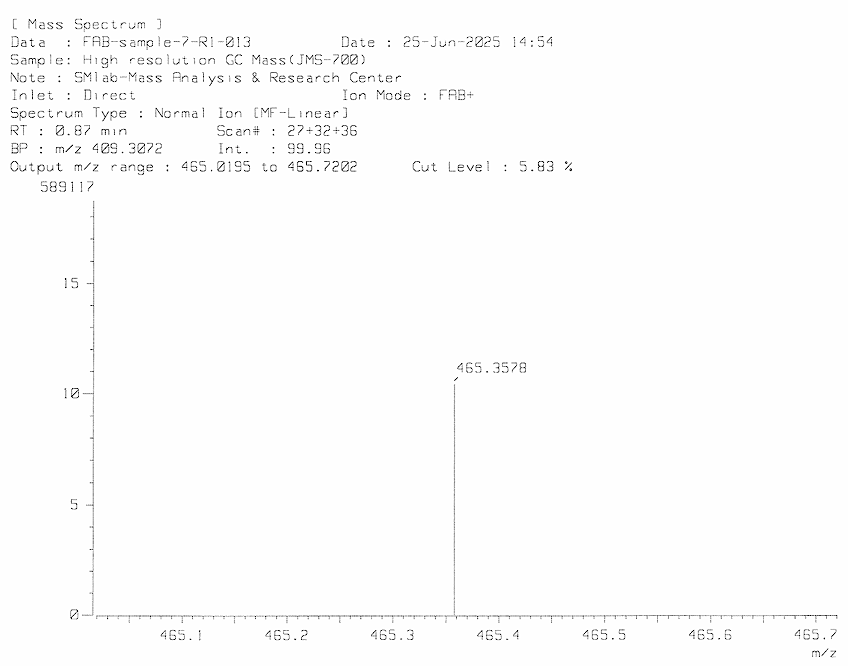


**Figure S51.** High resolution mass spectrum (HRMS) of CA-4.


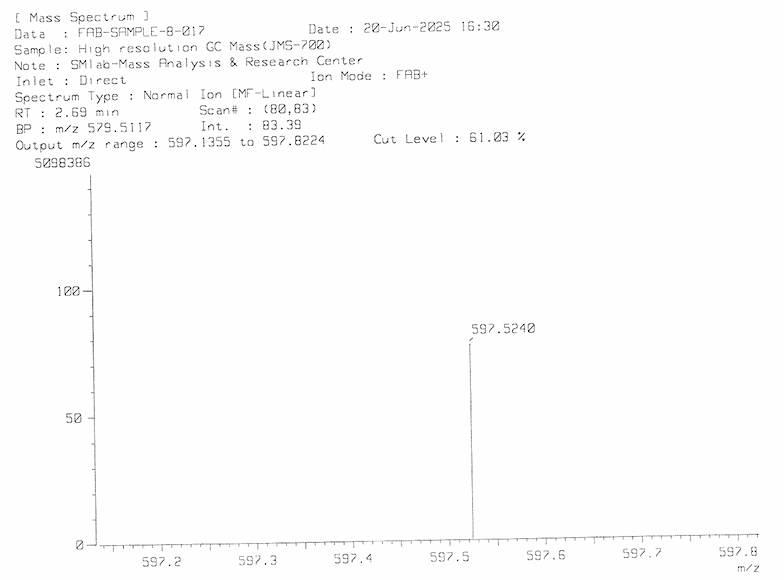


**Figure S52.** High resolution mass spectrum (HRMS) of CA-16.


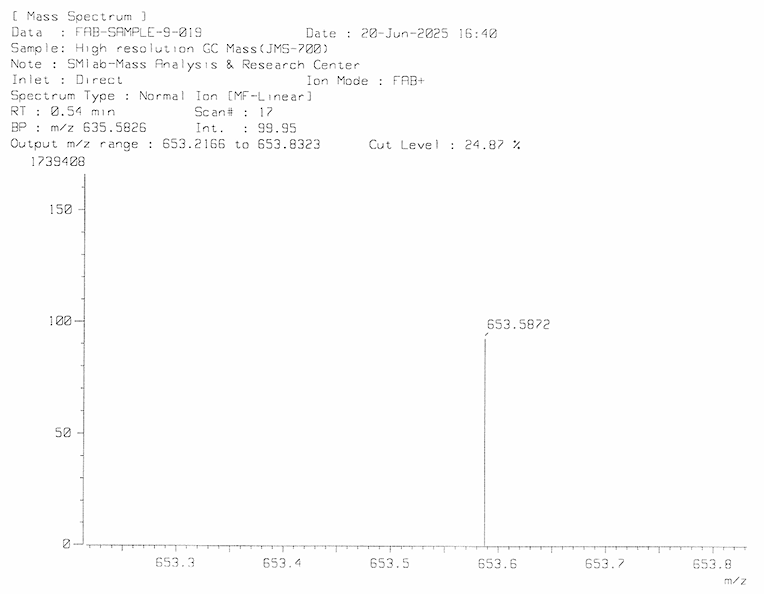


**Figure S53.** High resolution mass spectrum (HRMS) of CA-20.
